# Supplementary material for: Direct observation of electron transfer in solids through X-ray crystallography
Source: Nat Commun. 2024 May 23;15:4412. doi: 10.1038/s41467-024-48599-1 (PMC11116525; doi:10.1038/s41467-024-48599-1)
Supplement: Supplementary file 1 — Supplementary Information [file 41467_2024_48599_MOESM1_ESM.pdf]

Supplementary Information for

Direct observation of electron transfer in solids through X-ray crystallography

Authors: Daiji Ogata, Shota Koide, Hiroyuki Kishi, Junpei Yuasa*

Department of Applied Chemistry, Tokyo University of Science. 1-3 Kagurazaka, Shinjuku-ku, Tokyo 162-8601, Japan

*Corresponding author. Junpei Yuasa, Email: yuasaj@rs.tus.ac.jp.

Table of Contents

Supplementary Note 1. Supplementary Experimental Section

Supplementary Note 2. NMR-based determination of Zn_4-metallocycle $(Zn^{2+})_4(L_A)_8$
Supplementary Fig. 1 ^{1}H NMR spectra of L_A with $Zn(OTf)_2$.
Supplementary Fig. 2 DOSY NMR spectra of $(Zn^{2+})_4(L_A)_8$.
Supplementary Fig. 3 ^{1}H NMR spectra of $(Zn^{2+})_4(L_A)_8$ with $KSbF_6$.
Supplementary Fig. 4 Summary of ^{1}H NMR assignment of $(Zn^{2+})_4(L_A)_8$.
Supplementary Fig. 5 ROESY NMR spectrum of $(Zn^{2+})_4(L_A)_8$.
Supplementary Fig. 6 ^{1}H,^{1}H COSY NMR spectrum of $(Zn^{2+})_4(L_A)_8$.

Supplementary Note 3. X-ray structure analysis on $[(Zn^{2+})_4(L_A)_4(L_{A=O})_4]_n$
Supplementary Fig. 7 Electron density map ($F_o - F_c$) of $(Zn^{2+})_4(L_A)_4(L_{A=O})_4$.
Supplementary Fig. 8 Ratio between L_A and $L_{A=O}$ in the Zn_4-host frame.
Supplementary Fig. 9 ^{1}H NMR spectrum of $[(Zn^{2+})_4(L_A)_4(L_{A=O})_4]_n$ in DMF-d_7.
Supplementary Fig. 10 ^{1}H,^{1}H COSY NMR spectrum of $[(Zn^{2+})_4(L_A)_4(L_{A=o})_4]_n$.

Supplementary Note 4. Mechanism of slow oxidation-associated crystallization
Supplementary Fig. 11 Time course of molar ratio between L_A and $L_{A=O}$ during the crystallization of $[(Zn^{2+})_4(L_A)_4(L_{A=o})_4]_n$.
Supplementary Fig. 12 Dissociation and rearrangement of $[(Zn^{2+})_4(L_A)_4(L_{A=O})_4]_n$.
Supplementary Fig. 13 Oxidation of $(Zn^{2+})_4(L_A)_8$ under air and the deaerated conditions.
Supplementary Fig. 14 Crystal packing diagram of $[(Zn^{2+})_4(L_A)_4(L_{A=O})_4]_n$.

Supplementary Note 5. Porous ability of the nanotube crystals in the anion exchange
Supplementary Fig. 15 The procedures for the anion exchange experiment.
Supplementary Fig. 16 Molar ratio between $OSO_2CF_3^-$ and PF_6^- in the nanotube crystals.
Supplementary Fig. 17 Photograph of crystals of $[(Zn^{2+})_4(L_A)_4(L_{A=O})_4]_n(OSO_2CF_3^-)_{8n}$.
Supplementary Fig. 18 NMR titration of Zn_4-metallocycle by PF_6^-.
Supplementary Fig. 19 NMR titration of Zn_4-metallocycle by Fc.
Supplementary Fig. 20 NMR titration of Zn_4-metallocycle by TTF.

Supplementary Note 6. Absorption of Fc and TTF in the nanotube crystals
Supplementary Fig. 21 Visualization of cavity volume in $[(Zn^{2+})_4(L_A)_4(L_{A=O})_4]_n$.
Supplementary Fig. 22 Definition of window size of $[(Zn^{2+})_4(L_A)_4(L_{A=O})_4]_n$.

Supplementary Fig. 23 ^{1}H NMR spectrum of the Fc-soaking crystals in DMF-d_7.
Supplementary Fig. 24 Cyclic voltammogram of the TTF-soaking crystals of $[(Zn^{2+})_4(L_A)_4(L_{A=O})_4]_n$ in solution.
Supplementary Fig. 25 Interactions in $(Zn^{2+})_4(L_A)_4(L_{A=O})_4$, $(Fc)_2\subset(Zn^{2+})_4(L_A)_4(L_{A=O})_4$, and $(TTF)_2\subset(Zn^{2+})_4(L_A)_4(L_{A=O})_4$.
Supplementary Fig. 26 Photograph of $[(Zn^{2+})_4(L_A)_4(L_{A=O})_4]_n$ after soaking in solution containing Fc and TTF.
Supplementary Fig. 27 Representation of *P*- and *M*-helicity for the guest molecules.

Supplementary Note 7. Mechanism for ET oxidation
Supplementary Fig. 28 Cyclic voltammogram of Fc and TTF.
Supplementary Fig. 29 Solid state ESR spectra of the TTF-incorporated nanotube crystals.
Supplementary Fig. 30 The procedures for the solid-state ET oxidation.
Supplementary Fig. 31 Crystal structures of $[(Zn^{2+})_4(L_A)_4(L_{A=O})_4]_n$ before and after ET.
Supplementary Fig. 32 L_A:$L_{A=O}$ in $(TTF)_2\subset(Zn^{2+})_4(L_A)_4(L_{A=O})_4$ before and after ET.
Supplementary Fig. 33 L_A:$L_{A=O}$ in $(Fc)_2\subset(Zn^{2+})_4(L_A)_4(L_{A=O})_4$ before and after ET.
Supplementary Fig. 34 L_A:$L_{A=O}$ in the Zn_4-host frame before and after ET.
Supplementary Fig. 35 NMR-determined L_A:$L_{A=O}$ before and after ET.
Supplementary Fig. 36 Zn_4-host frame in $(TTF)_2\subset(Zn^{2+})_4(L_A)_4(L_{A=O})_4$ before and after ET.
Supplementary Fig. 37 Zn_4-host frame in $(Fc)_2\subset(Zn^{2+})_4(L_A)_4(L_{A=O})_4$ before and after ET.
Supplementary Fig. 38 Zn_4-host frame in $(Zn^{2+})_4(L_A)_4(L_{A=O})_4$ before and after ET.
Supplementary Fig. 39 Electron density map (F_o) of Zn_4-host frame before and after ET.
Supplementary Fig. 40 Crystal packing diagram of Zn_4-host frame before and after ET.
Supplementary Fig. 41 Core structure of the OTf···TTF unit.
Supplementary Fig. 42 Photograph of TTF crystal.
Supplementary Fig. 43 Schematic representation for the proposed mechanism of the hole accumulation in $[(TTF)_2\subset(Zn^{2+})_4(L_A)_4(L_{A=O})_4]_n$ by facial ET oxidation.
Supplementary Fig. 44 Comparison between the host window size and guest.
Supplementary Fig. 45 Oxidation of the nanotube crystal with magic blue.
Supplementary Fig. 46 Schematic representation for redox-stimuli responsive MOF and solid-state oxidation of $[(D)_2\subset(Zn^{2+})_4(L_A)_4(L_{A=O})_4]_n$ crystals.

Supplementary Note 8. Determination of reorganization energy of ET in solids
Supplementary Fig. 47 Core structures before and after ET oxidation.
Supplementary Fig. 48 Schematic representation of reorganization energy.
Supplementary Fig. 49 ^{1}H NMR spectrum of L_A.

Supplementary Fig. 50 ^{13}C NMR spectrum of L_A.

Supplementary Note 9. Crystallographic data and structure refinement details
Supplementary Table 1 $(Zn^{2+})_4(L_A)_4(L_{A=O})_4$
Supplementary Table 2 $(Fc)_2 \subset (Zn^{2+})_4(L_A)_4(L_{A=O})_4$
Supplementary Table 3 $(TTF)_2 \subset (Zn^{2+})_4(L_A)_4(L_{A=O})_4$
Supplementary Table 4 $(Zn^{2+})_4(L_{A=O})_8$
Supplementary Table 5 $(TTF)_2 \subset (Zn^{2+})_4(L_A)_4(L_{A=O})_4$ after ET
Supplementary Table 6 $(Fc)_2 \subset (Zn^{2+})_4(L_A)_4(L_{A=O})_4$ after ET

Supplementary References

Supplementary Note 1. Supplementary Experimental Section

Materials and Methods

Spectroscopic grade solvents used in this study, acetonitrile, 1,4-dioxane, chloroform, *n*-hexane and toluene were purchased from Wako Pure Chemical Industries, Ltd. Triethylamine were distilled, and the other chemicals for the synthesis were purchased from Kanto, Wako Pure Chemical Industries, Ltd., TCI, or Combi-Blocks and used without further purification. ^{1}H and ^{13}C NMR spectra were measured with JEOL JNM-ECZ400S, JNM-ECA500 and Bruker AVANCE NEO 400. UV-Vis absorption spectra were recorded by an JASCO V-660 at ambient temperature. High resolution Electrospray Ionization (HR-ESI) mass spectra were measured with mass spectrometers X500R QTOF (Sciex, MA, USA). DFT studies were performed with GAUSSIAN '09.[3]

Benzyltriethylammonium tribromide: Benzyltriethylammonium tribromide was synthesized in accordance with the literature procedures.[1] In particular, nitric acid (60%, 26.5 mL, 345 mmol) was added dropwise over 20 min to a water solution (120 mL in a two necked flask) containing benzyltriethylammonium bromide (20.0 g, 73.5 mmol) and potassium bromide (26.5 mg, 222 mmol). Then, the mixture was stirred at room temperature for 2 h to give orange solids, which was filtered and washed with water (100 mL) and ether (35 mL x 2). After the solvent was removed by evaporation, the resulting solids were dried under high vacuum to afford pure benzyl triethylammonium tribromide (26.8 g, 84.2%). ^{1}H NMR ($CDCl_3$, 300 MHz): δ 7.53 (m, 5H), 4.51 (s, 2H), 3.36 (q, 6H), 1.54 (t, 9H).

2,7-Dibromoacridine: 2,7-Dibromoacridine was synthesized following procedures reported in literature.[2] In particular, a methanol (200 mL) solution containing benzyltriethylammonium tribromide (26.8 g, 62.0 mmol) and acridine (5.00 g, 27.9 mmol) was heated at reflux for 16 h. After the reaction solution was cooled to ambient temperature, the resulting crude solids were filtered and washed with pyridine (10 mL) and dichloromethane (100 mL). The resulting product was dried under high vacuum to afford 2,7-dibromoacridine (7.35 g, 78.2%) as yellow solids. ^{1}H NMR ($CDCl_3$, 300 MHz): δ 8.59 (s, 1H), 8.18 (d, 2H), 8.12 (d, 2H), 7.85 (dd, 2H).

2,7-Bis(ethynyltrimethylsilane) acridine: 2,7-Dibromoacridine (3.51 g, 10.4 mmol), CuI (0.20 g, 1.04 mmol), $Pd(PPh_3)_4$ (1.07 g, 1.04 mmol), and dry triethylamine (60.0 mL),

were placed into a reaction flask equipped with a reflux condenser. The reaction flask was degassed with a nitrogen flow for 15 min. Then, ethynyltrimethylsilane (5.00 mL, 36.1 mmol) was added to the reaction mixture and heated to 75 °C under the Ar atmosphere conditions for 24 h. After the reaction mixture was cooled to room temperature, the resulting crude solids were filtered over a Celite pad. The crude product was extracted by chloroform. After the organic layer was dried over Na_2SO_4, the solvent was removed in vacuo. The crude was subjected to column chromatography on silica gel (chloroform) to afford a white brown solid (2.67 g, 69.0%). ^{1}H NMR (300 MHz, $CDCl_3$) δ 8.63 (s, 1H), 8.15 (s, 2H), 8.13 (d, 2H), 7.66–7.79 (m, 2H), 0.30 (s, 18 H).

2,7-Bis((1-ethyl-1*H*-imidazol-2-yl)ethynyl)acridine (L_A): To a two necked flask, CuI (176 mg, 0.928 mmol), 2,7-bis(ethynyltrimethylsilane)acridine (3.45 g, 9.28 mmol), 1-ethyl-2-iodo-1*H*-imidazole (5.15 g, 23.2 mmol), THF (80 mL), and triethylamine (60 mL) were added. After the solution was degassed by bubbling with Ar gas for 30 min, tetrabutylammonium fluoride (in tetrahydrofuran 1 mol/L, 27.8 mL) and $Pd(PPh_3)_4$ (1.07 g, 0.928 mmol) were added to the flask. Then, the reaction mixture was refluxed under Ar atmosphere for 24 h. The filtrate was extracted with chloroform. The organic phase was consecutively washed with water and then brine. It was dried over Na_2SO_4, filtered and the solvent was evaporated, the crude product was subjected to column chromatography on silica gel (chloroform/methanol = 9/1) and purified by GPC with chloroform to afford a yellow solid (1.26 g, 32.7%). ^{1}H NMR (500 MHz, 298 K, CD_3CN) δ 8.94 (s, 1H), 8.41 (s, 2H), 8.18 (d, J = 8.9 Hz, 2H), 7.93 (dd, J = 8.9, 1.7 Hz, 2H), 7.22 (s, 2H), 7.06 (s, 2H), 4.24 (q, J = 7.2 Hz, 4H), 1.48 (t, J = 7.2 Hz, 6H). ^{13}C NMR (125 MHz, 298 K, $CDCl_3$) δ 148.59, 135.79, 132.55, 131.94, 131.12, 130.10, 129.74, 126.27, 119.88, 119.78, 92.18, 80.63, 41.95, 16.07 ppm. HRMS (ESI): *m/z* calcd for $[C_{27}H_{21}N_5 + H]^+$: 416.18752; found: 416.18681.

X-ray crystallographic analysis

X-ray diffraction data were collected on Bruker-AXS・D8 QUEST using microfocus Mo*Kα* radiation (λ = 0.71073 Å) equipped with CCD detector. All data collection strategies were performed at 90 K using cold nitrogen streams. The structures were solved with the SHELXT[4] and refined by full matrix least-squares techniques against F^2 (SHELXL-2019/1)[5]. All displacements for non-hydrogen atoms were refined anisotropically while hydrogen atoms were refined isotropically using the riding model. In the refinement of the guest molecules absorbed in the Zn_4-host frame, we treated with appropriate crystallographic restraints like DFIX, DANG,

RIGU, SIMU, FLAT, ISOR and EADP. Disordered counterions and solvent molecules could not be determined the discrete atomic positions. The contribution of the electron density of such disordered molecules were handled using the SQUEEZE[6] routine in PLATON[7].

Supplementary Note 2. NMR-based determination of homoleptic Zn_4-metallocycle $(Zn^{2+})_4(L_A)_8$. Complex formation between the acridine ligand (L_A) and Zn^{2+} was examined by the ^1H NMR titration of L_A by $Zn(OTf)_2$ ($OTf^- = OSO_2CF_3^-$) in CD_3CN. The ^1H NMR of L_A exhibited two-step spectral changes in response to the molar ratio of $[Zn^{2+}]/[L_A]_0 = 0.50$ and 0.70, indicating stepwise assembly formation with Zn^{2+} (Supplementary Fig. 1). The latter Zn^{2+}-assembly formed at $[Zn^{2+}]/[L_A]_0 = 0.70$ is most likely a Zn_2-helicate complex $[(Zn^{2+})_2(L_A)_3]$, while no detailed characterization was done in this study. The ^1H NMR at $[Zn^{2+}]/[L_A]_0 = 0.50$ gave broad signals, while the all signals exhibited the same diffusion rate, a clear indication for exclusive formation of a single Zn_4-metallocycle $[(Zn^{2+})_4(L_A)_8]$. Hydrodynamic radius (R_h = 1.39 nm) determined by the DOSY NMR data agrees well with the size of the Zn_4-metallocycles. The broad NMR signals should be ascribed to a structural fluctuation of the Zn_4-metallocyclic frame (Supplementary Fig. S2e). Therefore, for obtaining the well-dissolved ^1H NMR spectrum, we used SbF_6^- as a guest anion to suppress the structural fluctuation of the Zn_4-metallocyclic frame. ^1H NMR titration of the Zn_4-metallocycle $[(Zn^{2+})_4(L_A)_8]$ by SbF_6^- suggested a 1:1 host-guest complex formation with the association constant of $K = (1.7 \pm 0.1) \times 10^3\ M^{-1}$ (Supplementary Fig. 3). The broad ^1H NMR signals became well-resolved upon addition of 10 mM of $KSbF_6$, while the diffusion rate remained unchanged (Supplementary Fig. 2). Thus, the structure of the Zn_4-metallocycle remained unchanged after the host-guest complex formation with SbF_6^-.

The resulting well-resolved ^1H NMR spectrum due to $(Zn^{2+})_4(L_A)_8$ exhibited 21 resonances at 5.5–9.0 ppm. Therefore, as compared with the ^1H NMR signals of free L_A, the ^1H NMR signals of L_A approximately splitted into four components after formation of the Zn_4-metallocycle (Supplementary Fig. 4). The observed ^1H NMR signal splitting pattern is consistent with the structure of Zn_4-metallocycle $[(Zn^{2+})_4(L_A)_8]$ with the double-wall structure. When a rhombus shape with C_2 symmetry is assumed for the Zn_4-metallocycle, the Zn_4-metallocycle is comprised of two L_A/L_A units with shorter Zn^{2+}-Zn^{2+} distance (hor-L_A/L_A) and the other two L_A/L_A units with longer Zn^{2+}-Zn^{2+} distance (vert-L_A/L_A), in which both hor-L_A/L_A and vert-L_A/L_A have inner and outer L_A ligands. Consequently, the eight L_A ligands in $(Zn^{2+})_4(L_A)_8$ are devised to four sets ligand pairs in the different conditions, i.e., hor-$L_A^{(in)}$, hor-$L_A^{(out)}$, vert-$L_A^{(in)}$, and vert-$L_A^{(out)}$ (Supplementary Fig. 4). According to this assumption, total eight imidazole protons should be found in the ^1H NMR spectrum of $(Zn^{2+})_4(L_A)_8$. Among them, the four imidazole protons showed NOE cross peaks with the ethyl protons at the imidazole rings (closed circles in Supplementary Fig. 5), because these imidazole protons are located close to the ethyl groups. Then, the

corresponding vicinal imidazole protons were determined by their COSY cross peaks (closed circles in Supplementary Fig. 6).

Next, we assigned the acridine protons at 1-, 2-, 3-, and 4- positions sequentially starting from the unique doublet signal H^n that showed a large upfield shift as compared to that of free L_A (Supplementary Fig. 4). The observed upfield shift of H^n is ascribed to the shielding effect by the stacked acridine ring in $(Zn^{2+})_4(L_A)_8$, therefore H^n can be assigned to the acridine protons at the 3,6-positions of $L_A^{(in)}$ (Supplementary Fig. 4). Besides, H^n had COSY cross peak with the doublet signal H^l (Supplementary Fig. 6), therefore H^l is the vicinal proton of H^n (the acridine protons at the 4,5-positions of $L_A^{(in)}$). Similarly to the unique doublet signal H^n, the singlet signal H^m also showed a large upfield shift (Supplementary Fig. 4), indicating that H^m corresponds to the acridine protons at the 2,7-positions of $L_A^{(in)}$. Besides, the singlet signal H^m showed a weak COSY cross peak with the doublet signal H^k (Supplementary Fig. 6), therefore H^k should be the acridine protons at 3,6-positions of $L_A^{(in)}$. The corresponding vicinal protons (H^i) can be determined by the COSY NMR spectrum (Supplementary Fig. 6). Besides, the unique singlet signal H^m had a NOE correlation with the singlet signal H^g (Supplementary Fig. 5), suggesting that H^g should be the neighboring (stacked) acridine protons at the 1,8-positions of $L_A^{(out)}$ (Supplementary Fig. 4). The singlet signal H^g showed a weak COSY cross peak with the singlet signal H^a and H^c (Supplementary Fig. 6), hence H^a and H^b can be assigned to the acridine protons at the 9-position of $L_A^{(out)}$ (Supplementary Fig. 4). Conversely, the unique singlet signal H^m showed chemical exchange with the singlet signal H^f due to exchange (Supplementary Fig. 5), indicating that H^f should be the acridine protons at the 1,8-positions of the other $L_A^{(in)}$ ligands (Supplementary Fig. 4). The other ^{1}H NMR signals (H^b, H^d, H^e, H^h, and H^i) were assigned to the rest of acridine protons (Supplementary Fig. 4). Furthermore, Zn_4-metallocycle $[(Zn^{2+})_4(L_A)_8]$ was successfully detected upon ESI MS analysis of an acetonitrile solution containing L_A with $Zn(OTf)_2$ (Supplementary Fig. 2c).

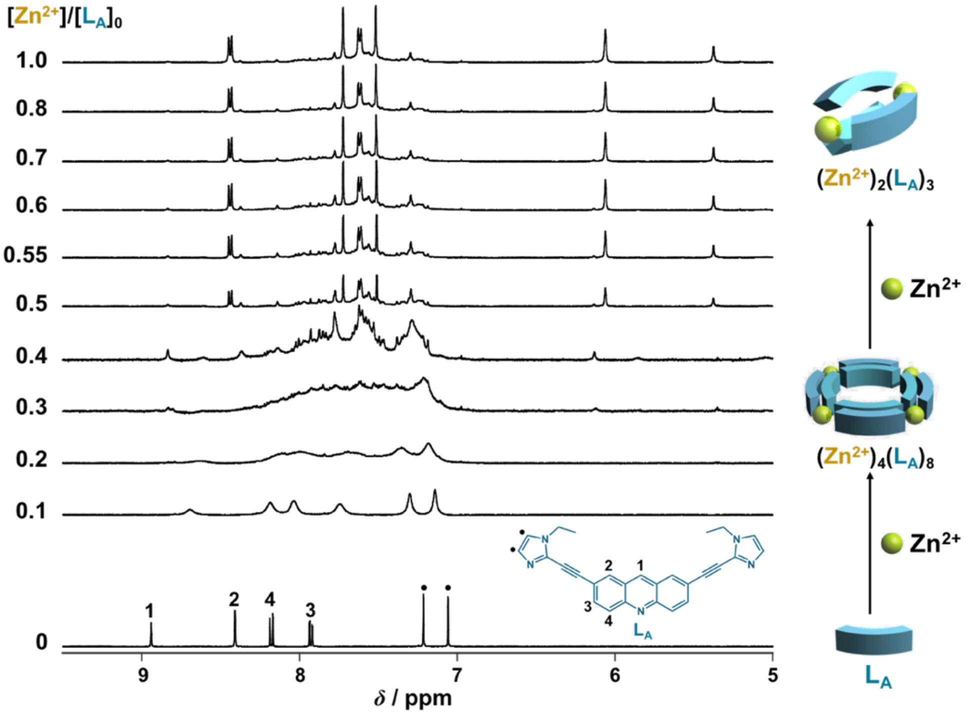

Supplementary Fig. 1 Stacked ^{1}H NMR spectra of L_A (2.0 mM) in the presence of $Zn(OTf)_2$ (0–2.0 mM) in CD_3CN at 298 K.

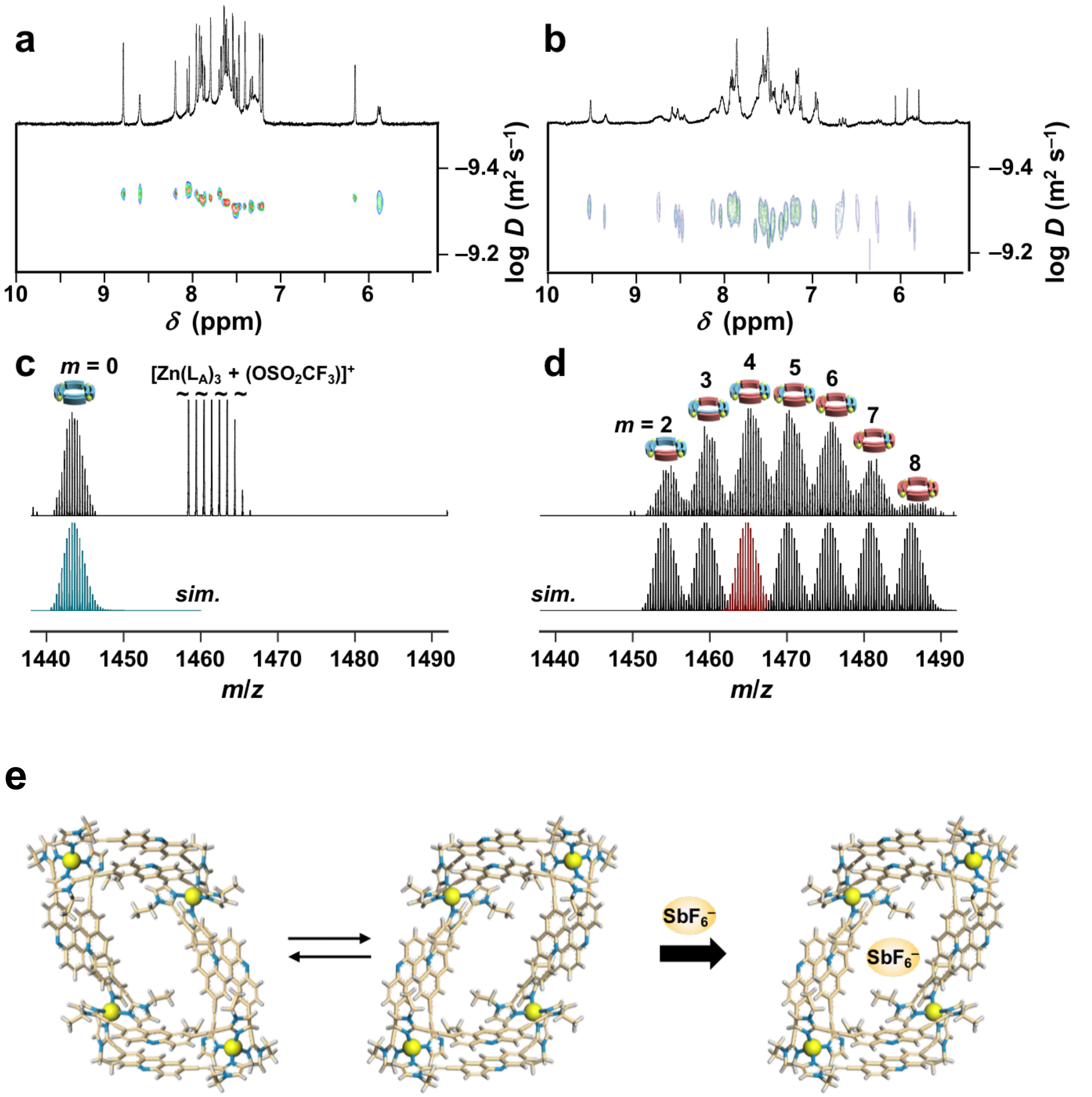

Supplementary Fig. 2 (a and b) DOSY NMR spectra of (a) in-situ prepared $(Zn^{2+})_4(L_A)_8$ (0.50 mM) in CD_3CN containing $KSbF_6$ (10 mM), (b) the double-wall nanotube crystals ($[(Zn^{2+})_4(L_A)_4(L_{A=O})_4]_n$) dissolved in CD_3CN containing $KSbF_6$ (10 mM) at 298 K. (c and d) ESI-mass spectra of (c) $(Zn^{2+})_4(L_A)_8$ and (d) $[(Zn^{2+})_4(L_A)_4(L_{A=O})_4]_n$ dissolved in acetonitrile. Bottom panels show simulated ESI mass spectra of $[Zn_4(L_A)_{(8-m)}(L_{A=O})_m + 5(OSO_2CF_3)]^{3+}$. (e) Schematic representation for the structural fluctuation of the Zn_4-metallocyclic frame, and the structure fixation by SbF_6^-.

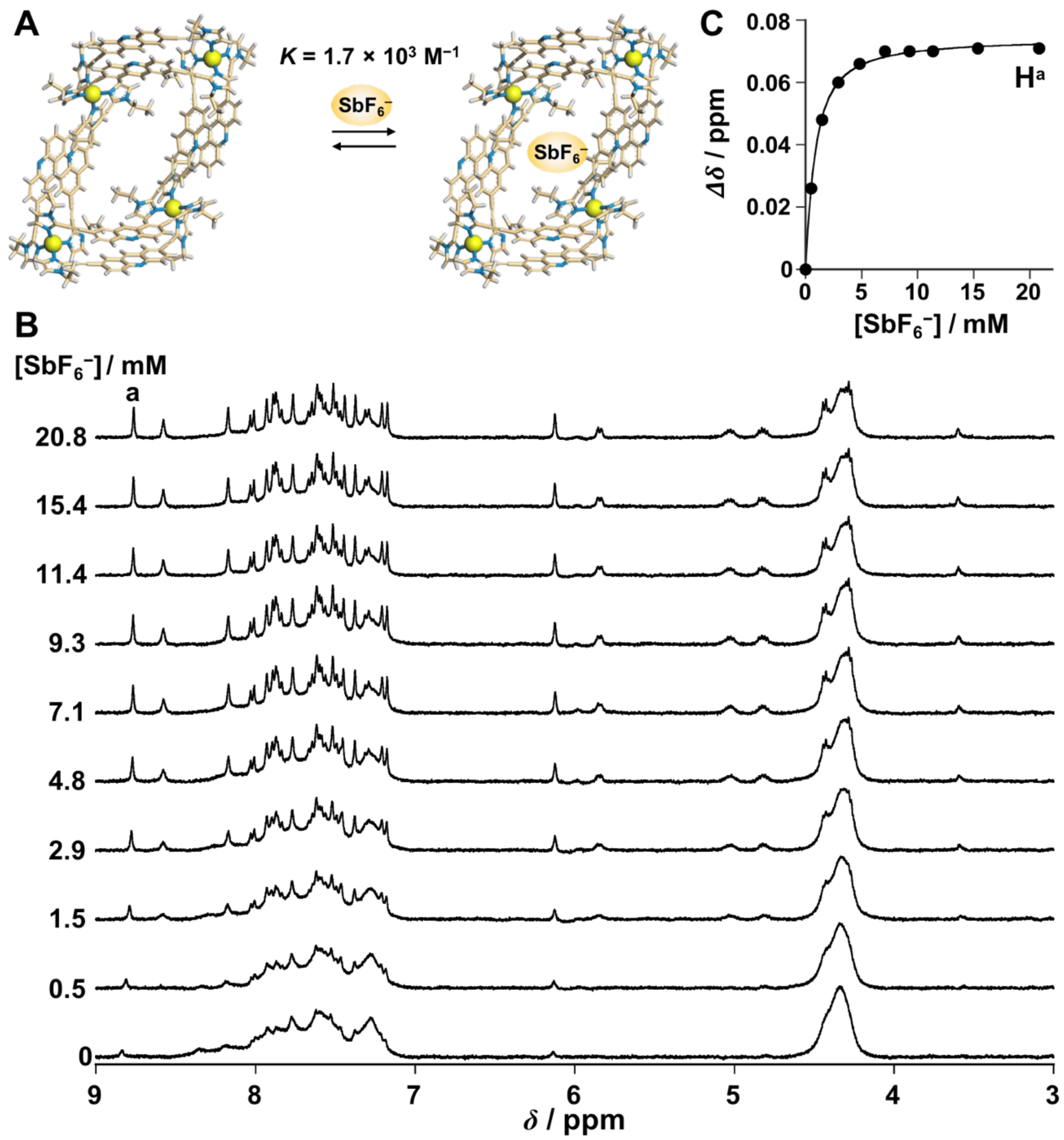

Supplementary Fig. 3 (A) Schematic representation for host-guest complex formation between $(Zn^{2+})_4(L_A)_8$ and SbF_6^-. (B) Stacked ^1H NMR spectra of $(Zn^{2+})_4(L_A)_8$ (0.50 mM) in the presence of $KSbF_6$ (0–20.8 mM) in CD_3CN at 298 K. (C) Plot of chemical shift (H^a) versus concentration of $[SbF_6^-]$. Solid line shows the theoretical fitting curve based on the 1:1 host-guest complex formation.

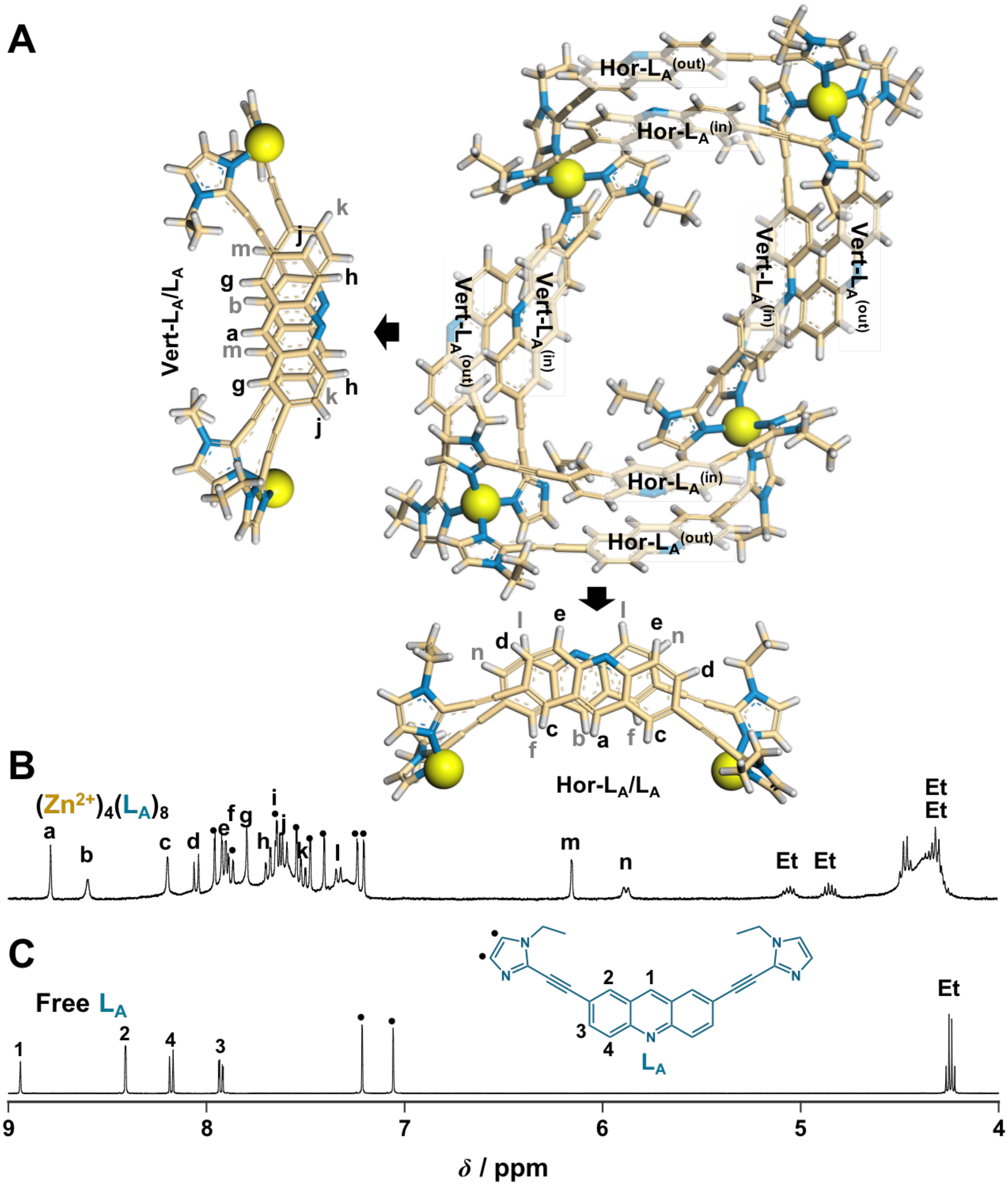

Supplementary Fig. 4 (A) Summary of ^{1}H NMR assignment of $(Zn^{2+})_4(L_A)_8$. (B,C) ^{1}H NMR spectra of (B) $(Zn^{2+})_4(L_A)_8$ (0.50 mM) in the presence of $KSbF_6$ (10 mM), and (C) free L_A in CD_3CN at 298 K.

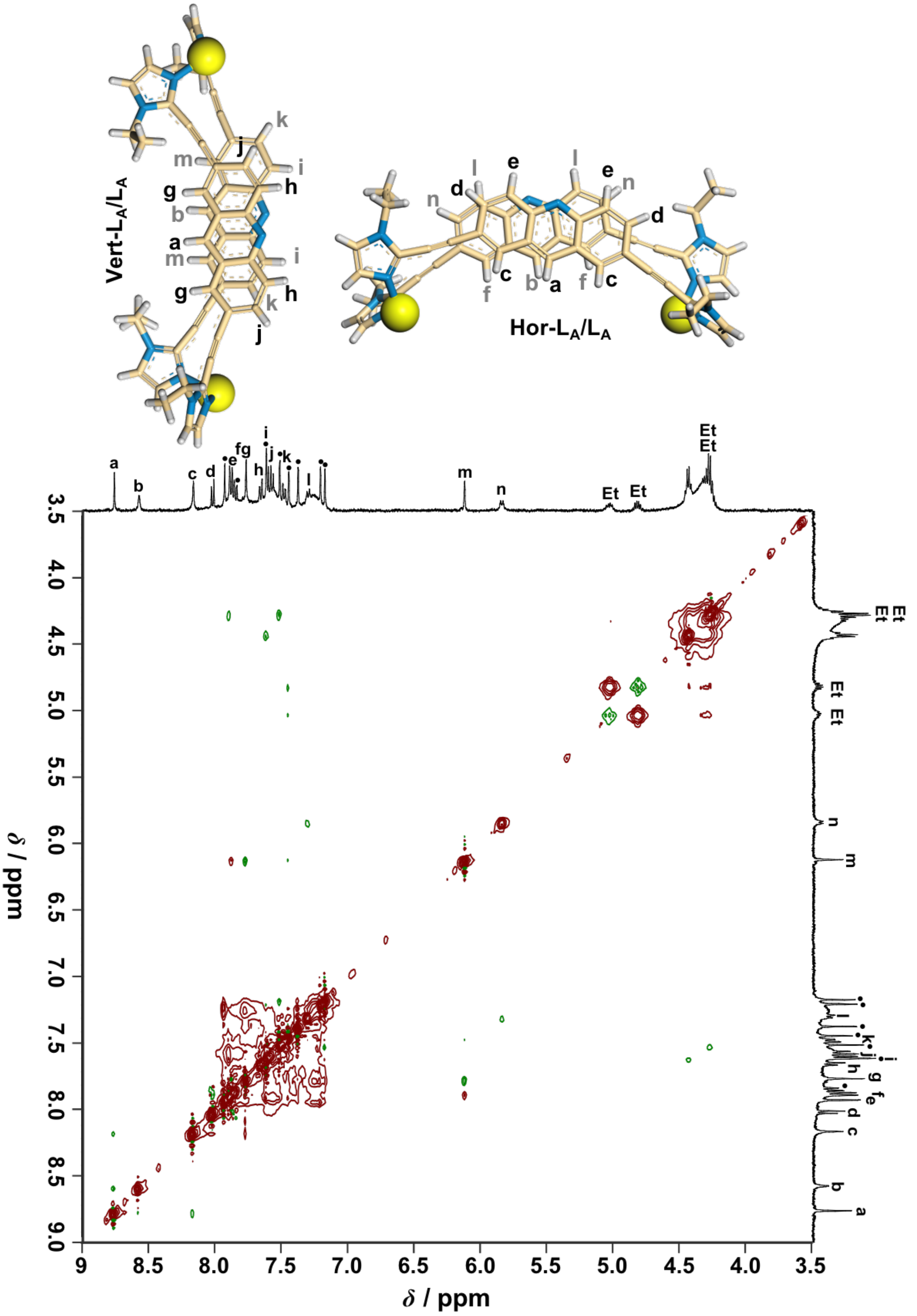

Supplementary Fig. 5 ROESY NMR spectrum of $(Zn^{2+})_4(L_A)_8$ (0.50 mM) in the presence of $KSbF_6$ (10 mM) in CD_3CN at 298 K.

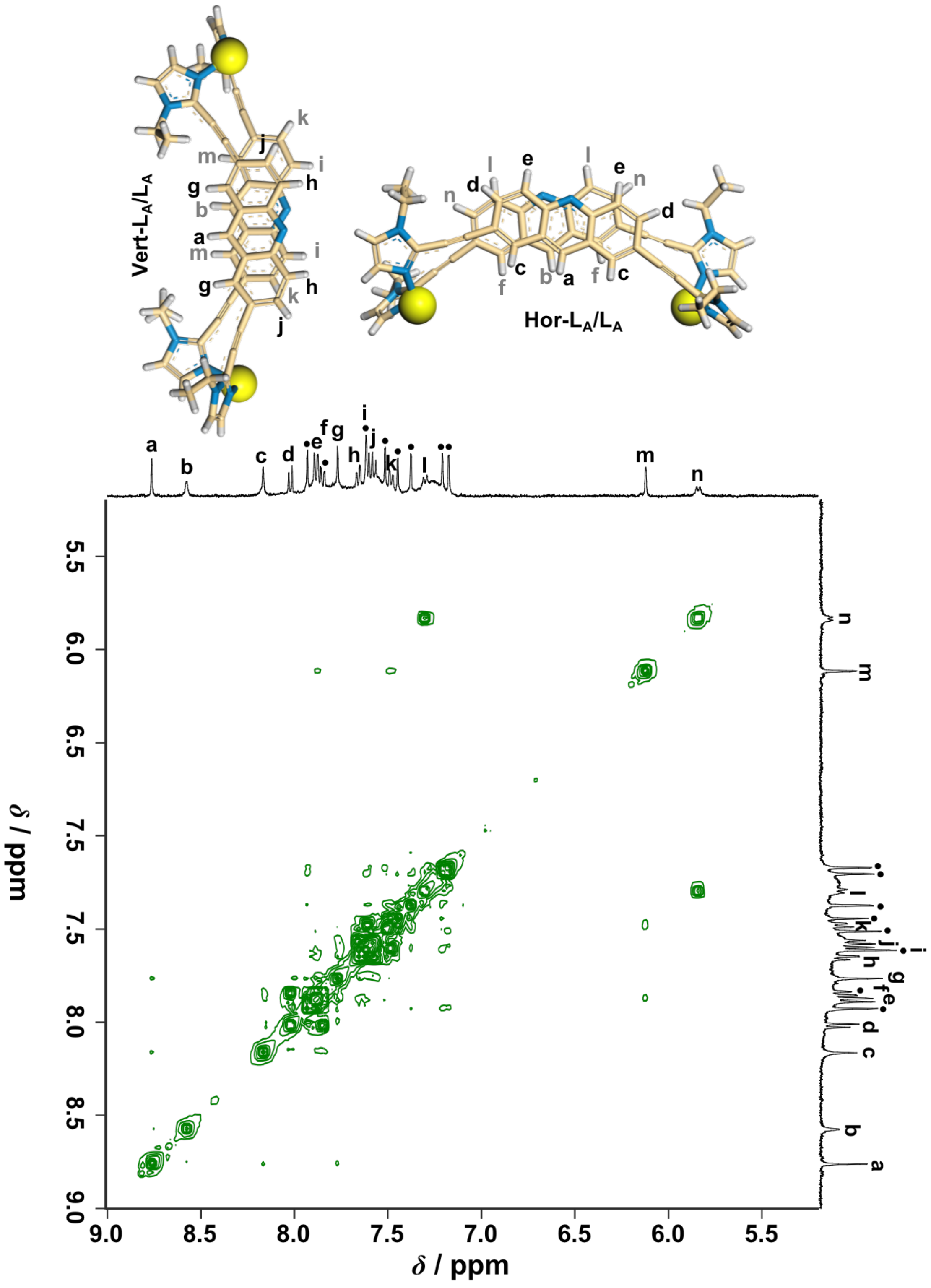

Supplementary Fig. 6 $^1H,^1H$ COSY NMR spectrum of $(Zn^{2+})_4(L_A)_8$ (0.50 mM) in the presence of $KSbF_6$ (10 mM) in CD_3CN at 298 K.

Supplementary Note 3. X-ray structure analysis on $[(Zn^{2+})_4(L_A)_4(L_{A=O})_4$. During the X-ray crystal structure analysis of the double-wall nanotube crystal ($[(Zn^{2+})_4(L_A)_4(L_{A=O})_4]_n\cdot(OTf^-)_{8n}$), it was revealed that the Zn_4-host frame had a rhombus shape consisting of cyc-Zn^{2+}–$L_{hor}^{(in)}$/$L_{hor}^{(out)}$–Zn^{2+}–$L_{vert}^{(in)}$/$L_{vert}^{(out)}$– Zn^{2+}–$L_{hor}^{(in)}$/$L_{hor}^{(out)}$–Zn^{2+}–$L_{vert}^{(in)}$/$L_{vert}^{(out)}$ sequence (Supplementary Fig. 7). Besides, the double-wall nanotube crystals contained L_A and $L_{A=O}$ in 1:1 ratio, which was determined by the ^{1}H NMR spectrum measured by dissolving the crystals in DMF-d_7 (Supplementary Figs. 9 and 10). Since L_A and $L_{A=O}$ are structural isomorphous, there is a possibility of random positioning of L_A and $L_{A=O}$ in the crystal structure of $[(Zn^{2+})_4(L_A)_4(L_{A=O})_4]_n$. Conversely, the resolution of the X-ray diffraction data can determine the absence and presence of electron density on the carbon atoms at the 9-psotions of L_A and $L_{A=O}$, respectively. When $L_{hor}^{(in)}$/$L_{hor}^{(out)}$ and $L_{vert}^{(in)}$/$L_{vert}^{(out)}$ were assigned as L_A/L_A and $L_{A=O}$/$L_{A=O}$, respectively (the opposite sequence as that defined in the final X-ray structure), the electron density ($F_o - F_c$) analysis showed deficiency of electron density on the oxygen atoms of $L_{A=O}$/$L_{A=O}$ and residual electron density on the carbon atoms of L_A/L_A at the 9-position (Supplementary Fig. 7B). Conversely when $L_{hor}^{(in)}$/$L_{hor}^{(out)}$ and $L_{vert}^{(in)}$/$L_{vert}^{(out)}$ were assigned as $L_{A=O}$/$L_{A=O}$ and L_A/L_A, respectively (the same sequence as that defined in the final X-ray structure), there is no huge residual electron density and deficiency of electron density in the electron density map ($F_o - F_c$) (Supplementary Fig. 7A). Thus, the relative positions of L_A and $L_{A=O}$ are almost fixed in $[(Zn^{2+})_4(L_A)_4(L_{A=O})_4]_n$, where the cyc-$Zn^{2+}$–$L_{A=O}$/$L_{A=O}$–$Zn^{2+}$–$L_A$/$L_A$–$Zn^{2+}$–$L_{A=O}$/$L_{A=O}$–$Zn^{2+}$–$L_A$/$L_A$ is the major sequence in the Zn_4-host frame. Conversely, slight deficiency of electron density was observed at the oxygen atoms of the outer $L_{A=O}$ ligands in the electron density analysis ($F_o - F_c$) of the final X-ray structure (Supplementary Fig. 7A), indicating that the positions of $L_{hor}^{(out)}$ were partially replaced by L_A in statistics (L_A:$L_{A=O}$ = 58:42, Supplementary Fig. 8).

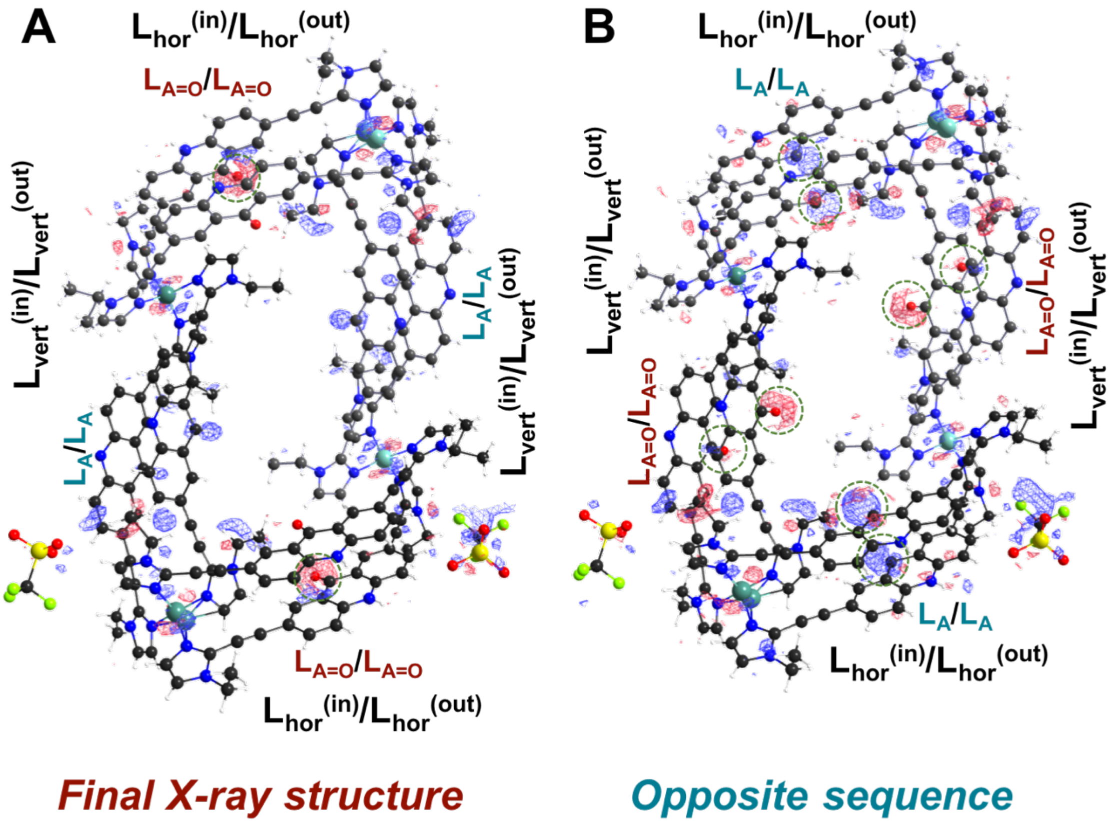

Supplementary Fig. 7 Electron density map ($F_o - F_c$) of $(Zn^{2+})_4(L_A)_4(L_{A=O})_4$ assigned hor-$L^{(in)}/L^{(out)}$ and vert- $L^{(in)}/L^{(out)}$ as (A) $L_{A=O}/L_{A=O}$ and L_A/L_A, (B) L_A/L_A and $L_{A=O}/L_{A=O}$, respectively.

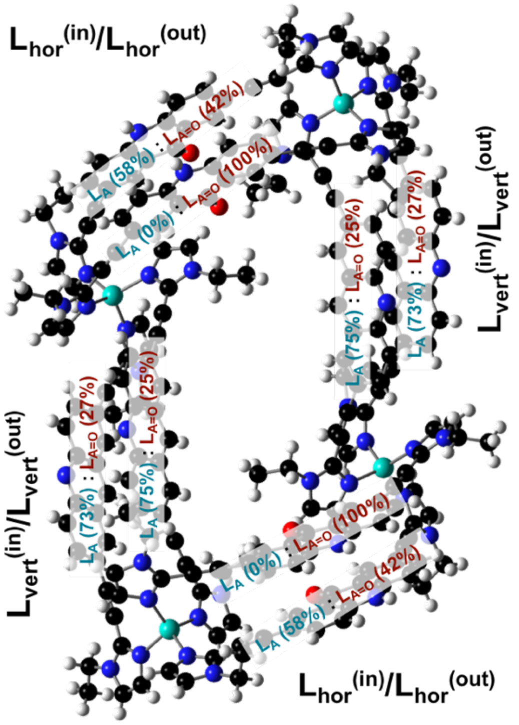

Supplementary Fig. 8 Ratio between L_A and $L_{A=O}$ in the Zn_4-host frame determined by the electron density ($F_o - F_c$) analysis of the oxygen atoms of the 9-positions of the acridone rings.

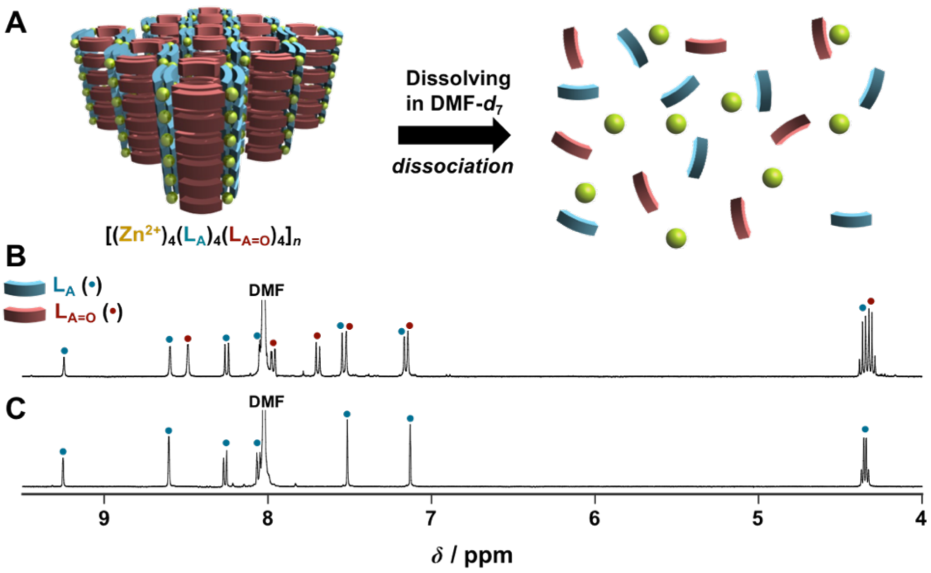

Supplementary Fig. 9 (A) Schematic representation for dissociation of $[(Zn^{2+})_4(L_A)_4(L_{A=O})_4]_n$ upon dissolving in DMF-d_7. (B) ^{1}H NMR spectrum of the $[(Zn^{2+})_4(L_A)_4(L_{A=O})_4]_n$ crystals upon dissolving DMF-d_7. (C) ^{1}H NMR spectrum of free L_A in DMF-d_7.

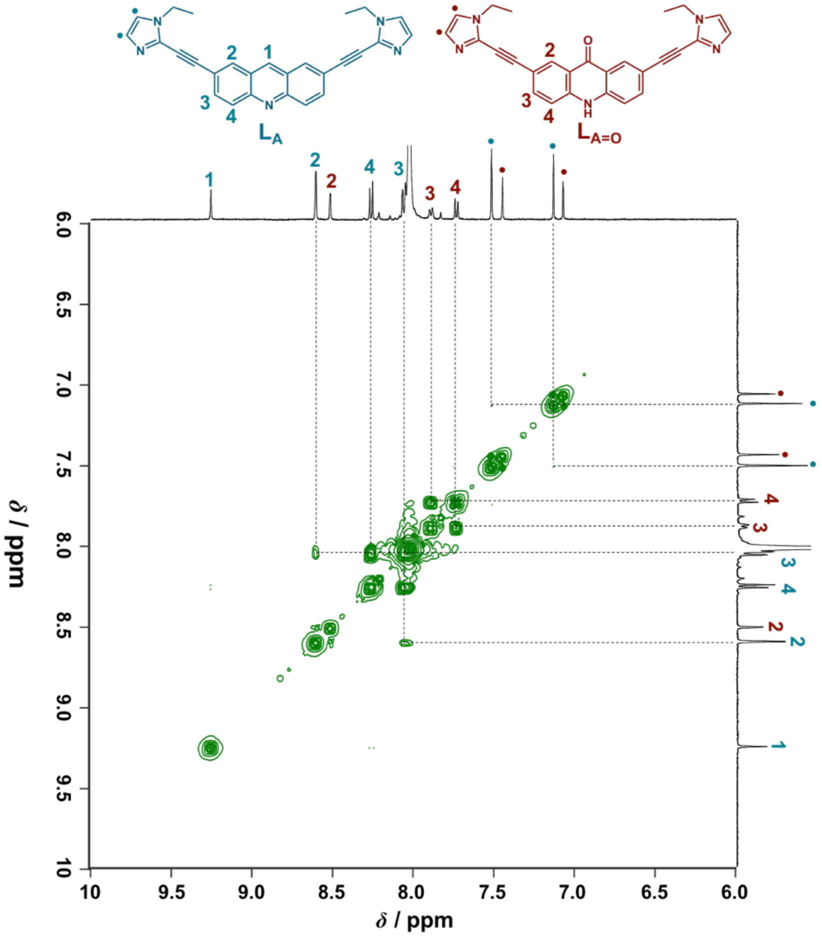

Supplementary Fig. 10 ^{1}H,^{1}H COSY NMR spectrum of the $[(Zn^{2+})_4(L_A)_4(L_{A=o})_4]_n$ crystals upon dissolving DMF-d_7.

Supplementary Note 4. Mechanism of slow oxidation-associated crystallization (SOAC). The acridone ligands ($L_{A=O}$) found in the double-wall nanotube crystal ($[(Zn^{2+})_4(L_A)_4(L_{A=O})_4]_n$) are formed by oxidation of acridine ligands (L_A) by oxygen[8] during the crystallization process. To verify the supramolecular crystallization mechanism, we monitored the ratio of the oxidized ligand over the total ligand concentration ($[L_{A=O}]/([L_A] + [L_{A=O}])$) in the crystallization solvent during crystallization. The oxidized ligand ratio gradually increases with time, becoming saturated at $[L_{A=O}]/([L_A] + [L_{A=O}]) = 0.5$ at ~400 h (Supplementary Fig. 11). This observation supports the proposed SOAC crystallization mechanism, because the oxidized ligands ($L_{A=O}$) gradually form in the crystallization solvent continue to be consumed by crystal growth at a steady state, and therefore not all the L_A ligands are oxidized to $L_{A=O}$ ligands during the crystallization process (Fig. 1a).

Conversely, upon dissolving the double-wall nanotube crystals ($[(Zn^{2+})_4(L_A)_4(L_{A=O})_4]_n$) in

CD_3CN, the ^1H NMR shows broad signals, even in the presence of the guest anion SbF_6^- (Supplementary Fig. 2b), and each resonance exhibits approximately the same diffusion rate as that of the homoleptic Zn_4-metallocycle $[(Zn^{2+})_4(L_A)_8]$ (Supplementary Fig. 2a *vs*. 2b). Hence, the NMR peak broadening of the resonances originates from a statistical species distribution for the heteroleptic Zn_4-metallocycles $((Zn^{2+})_4(L_A)_{(8-m)}(L_{A=O})_m)$, where the double-wall nanotube $([(Zn^{2+})_4(L_A)_4(L_{A=O})_4]_n)$ dissociates into the building metallocycles (Supplementary Fig. 12). As expected, ESI MS analysis of an acetonitrile solution of the obtained nanotube crystals presents mass signals arising from a statistical mixture of heteroleptic Zn_4-metallocycles with various $L_A/L_{A=O}$ ratios $((Zn^{2+})_4(L_A)_{(8-m)}(L_{A=O})_m$, m = 2–8; Supplementary Fig. 2d).

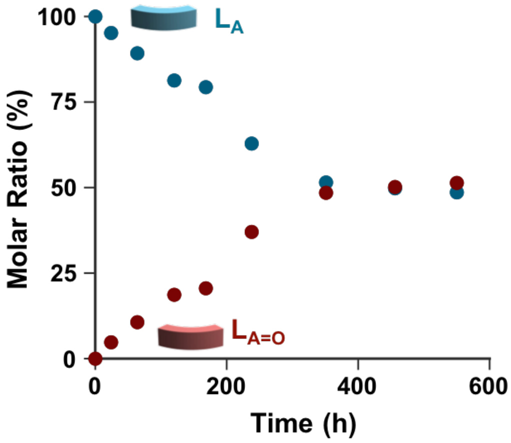

Supplementary Fig. 11 Time course of molar ratio between L_A (blue circles) and $L_{A=O}$ (red circles) in the crystallization solvent during the crystallization of $[(Zn^{2+})_4(L_A)_4(L_{A=O})_4]_n$.

The oxidation of L_A to $L_{A=O}$ occurred mostly in the solution phase prior to the crystallization to form the double-wall nanotube, giving a mixture of $(Zn^{2+})_4(L_A)_m(L_{A=O})_{(8-m)}$ (m = 0–8) host compounds in the solution (Fig. 1a). Conversely, oxidation of the $(Zn^{2+})_4(L_A)_4(L_{A=O})_4$ host frame could not be occurred in the crystalline state (without using the $[Fe(H_2O)_6](ClO_4)_3$ solid). Among the $(Zn^{2+})_4(L_A)_m(L_{A=O})_{(8-m)}$ host compounds, the $(Zn^{2+})_4(L_A)_4(L_{A=O})_4$ host frame favorably crystallizes through the slow-oxidation-associated crystallization process. In such a case, the composition of $(Zn^{2+})_4(L_A)_4(L_{A=O})_4$ is frozen in the crystal, and therefore the other four L_A ligands unchanged to $L_{A=O}$ during the crystallization process.

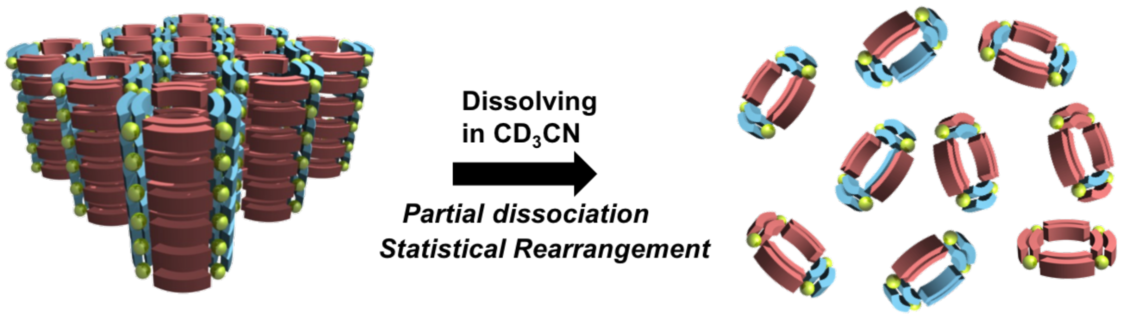

Supplementary Fig. 12 Schematic representation for partial dissociation and statistical rearrangement of $[(Zn^{2+})_4(L_A)_4(L_{A=O})_4]_n$ upon dissolving in CD_3CN.

The source of oxygen atom in the present system (SOAC) is most probably oxygen in air. DFT calculation (CAM-B3LYP/6-31G+(d,p)) suggests that the oxidation reaction, $L_A + 1/2O_2 \rightarrow L_{A=O}$, is 53.3 kcal mol^{-1} energetically down hill (while this is spin-forbidden oxygenation). In this context, we performed the negative control experiments (see below). 3 days standing of the acetonitrile/1,4-dioxane mixed solution (1/2, vol/vol) of L_A containing $Zn(OTf)_2$ ($OTf^- = OSO_2CF_3^-$) *under air* resulted in conversion of L_A to $L_{A=O}$ partially (Supplementary Fig. 13a and d). Hence, the oxidation from L_A to $L_{A=O}$ proceeds slowly in the presence of oxygen. Conversely, almost no oxidized product ($L_{A=O}$) was formed in that performed *under the deaerated conditions* (Supplementary Fig. 13b and c). The negative result obtained *under the deaerated conditions* suggests that oxygen in air is the source of oxygen atom in the present system.

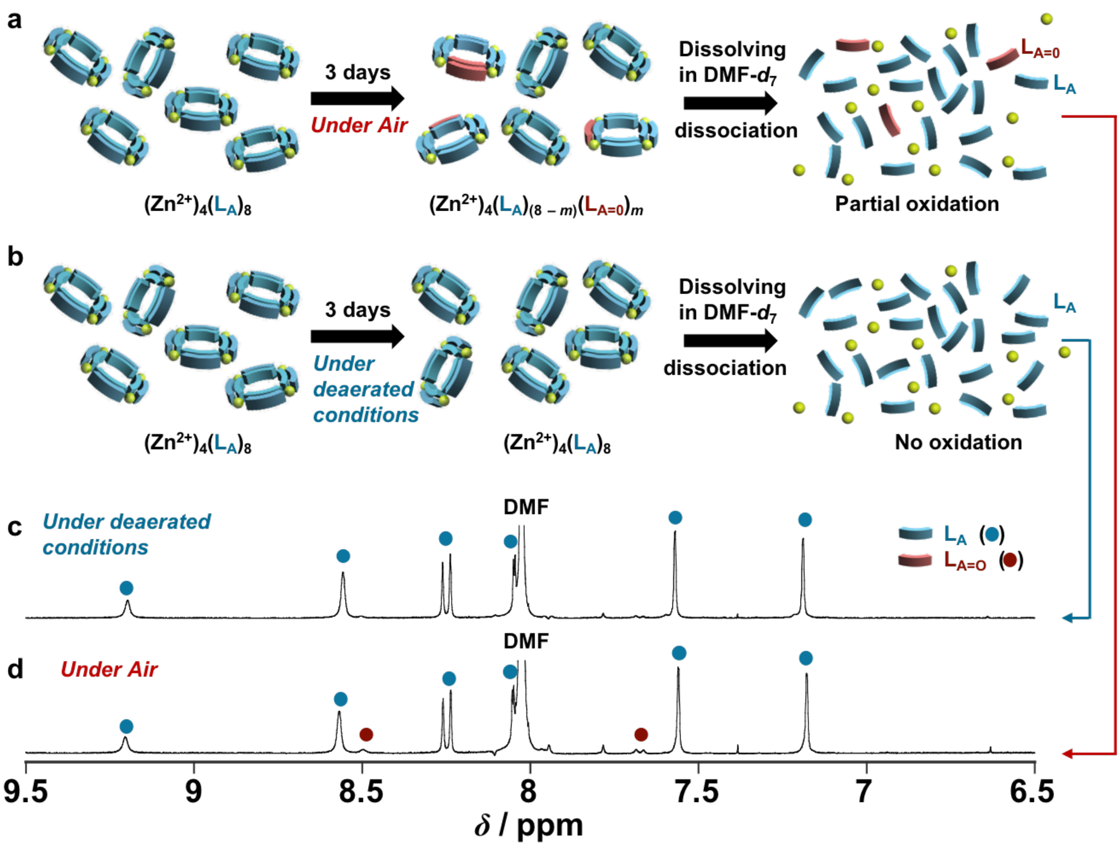

Supplementary Fig. 13 (a,b) Schematic representation for the oxidation experiments of $(Zn^{2+})_4(L_A)_8$ in (a) under air and (b) under the deaerated conditions: Acetonitrile/1,4-dioxane mixed solutions (1/2, vol/vol) of L_A (2.0 mM) containing $Zn(OTf)_2$ (2.0 mM) were standing for 3 days (a) under air and (b) under the deaerated conditions. Then, the solvent of the resulting solutions were removed by evaporation, and the crude products were dissolved in DMF-d_7. (c,d) ^{1}H NMR spectra (in DMF-d_7) of the resulting samples obtained (c) under the deaerated conditions and (d) under air.

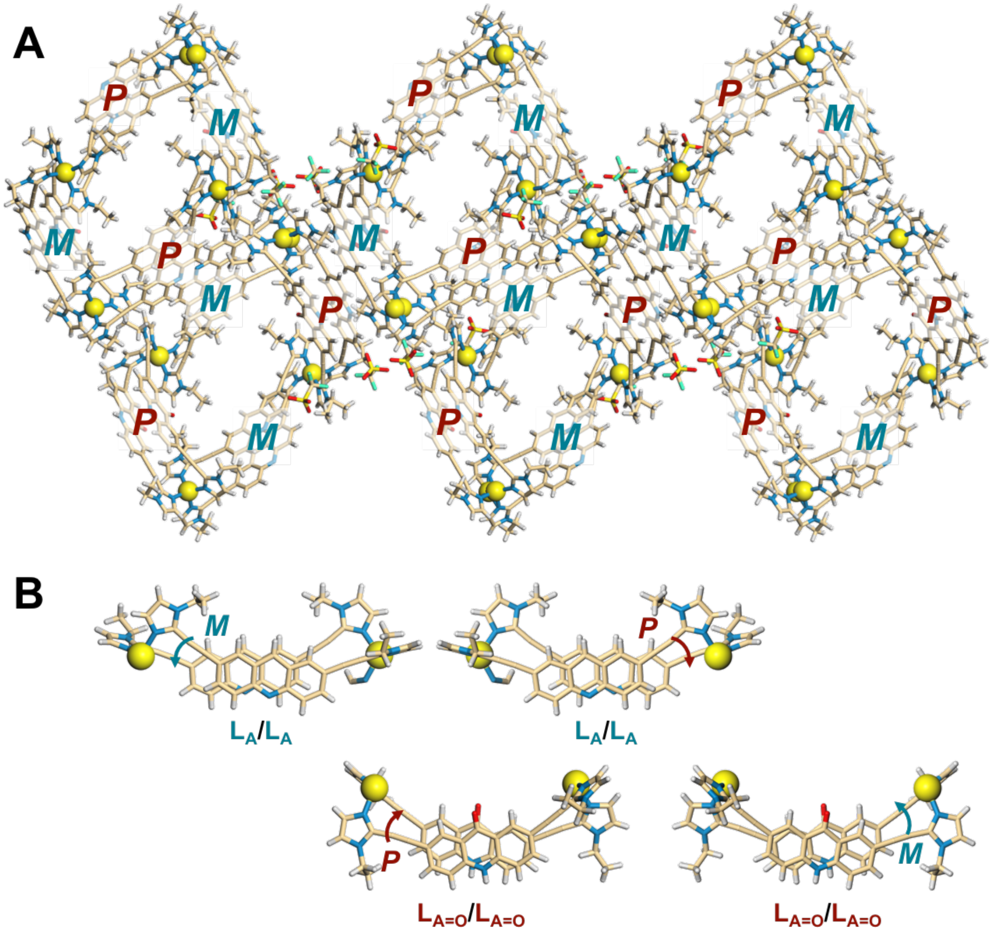

Supplementary Fig. 14 (A) *P*- and *M*-helicity of the L_A/L_A and $L_{A=O}/L_{A=O}$ units in crystal packing diagram of $[(Zn^{2+})_4(L_A)_4(L_{A=O})_4]_n$. (B) Representation for *P*- and *M*-helicity of the L_A/L_A and $L_{A=O}/L_{A=O}$ units in the $(Zn^{2+})_4(L_A)_4(L_{A=O})_4$ frame.

Supplementary Note 5. Porous ability of the nanotube crystals in the anion exchange experiments. X-ray crystal structure analysis of the double-wall nanotube crystal ($[(Zn^{2+})_4(L_A)_4(L_{A=O})_4]_n\cdot(OTf^-)_{8n}$) revealed that six OTf^- molecules are fixed and located outside the Zn_4-host frames (Fig. 2c), in which the two OTf^- molecules located around the acridone oxygen atoms are disordered and therefore removed by the PLATON/SQUEEZE program.[6,7] The rest of two OTf^- molecules should be confined inside the tube and the void space in the crystal lattice. In the present study, the porous ability of the nanotube crystal ($[(Zn^{2+})_4(L_A)_4(L_{A=O})_4]_n\cdot(OTf^-)_{8n}$) to pass guest molecules was firstly examined by the anion-change experiment with PF_6^- in a crystalline state as described in Supplementary Fig. 15. ^{1}H NMR titration of the Zn_4-metallocycle by PF_6^- suggested that PF_6^- had a binding affinity with the Zn_4-host frame (Supplementary Fig.

18). Upon soaking of the nanotube crystals into the acetonitrile/1,4-dioxane mixed solution (1/2, vol/vol) containing $NBu_4 \cdot PF_6$, total 25% of the OTf^- molecules in the nanotube crystals ($[(Zn^{2+})_4(L_A)_4(L_{A=O})_4]_n \cdot (OTf^-)_{8n}$) were quickly exchanged by PF_6^- (Supplementary Fig. 16), while the PF_6^--soaking crystals kept crystallinity (Supplementary Fig. 17). The exchanged 25% OTf^- molecules should correspond to the two OTf^- molecules confined inside the tube. Thus, the inner cavity of the nanotube crystal has porous ability to adsorb and pass the incoming guest anions, albeit the inner cavity of the nanotube confines the OTf^- molecules that loosely bind with the Zn_4-host frame.

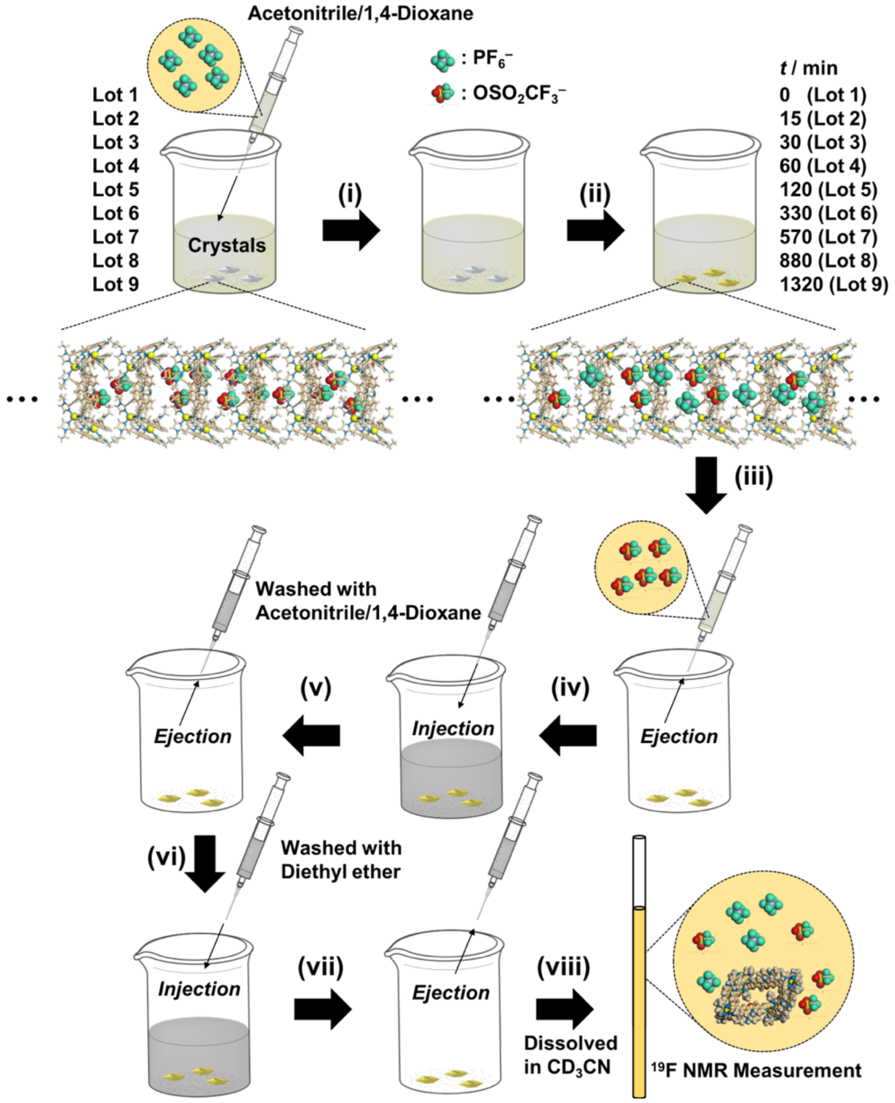

Supplementary Fig. 15 Schematic representation for the procedures for the anion exchange experiment of in the double-wall nanotube crystals ($[(Zn^{2+})_4(L_A)_4(L_{A=O})_4]_n(OSO_2CF_3^-)_{8n}$). (i) Crystals of $[(Zn^{2+})_4(L_A)_4(L_{A=O})_4]_n(OSO_2CF_3^-)_{8n}$ were placed into sample tubes (Lot 1–9, 0.5 mg on average). Then, 50 µL of acetonitrile/1,4-dioxane mixed solution (1/4, vol/vol) containing $NBu_4{\cdot}PF_6$ (96 mM) was added. (ii) The resulting solutions were left to stand for 0, 15, 30, 60, 120, 330, 570, 880, and 1320 min (Lot 1–9, respectively). (iii) Then, the solution phase was removed by syringe. (iv, v) The PF_6^--soaking crystals were washed by acetonitrile/1,4-dioxane mixed solution (1/4, vol/vol) 3 times, (vi, vii) and were further washed by diethyl ether 2 times. Then the samples were dried in a vacuum. (viii) Then, the PF_6^--soaking crystals were dissolved in CD_3CN, and the ratios between $OSO_2CF_3^-$ and PF_6^- were determined by ^{19}F NMR spectroscopy (Supplementary Fig. 16).

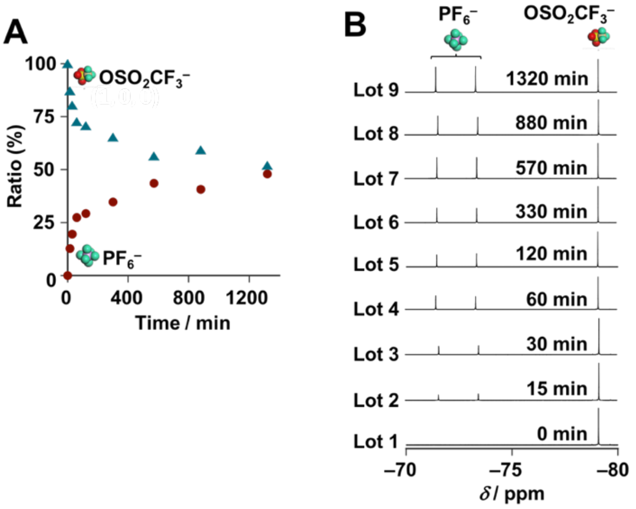

Supplementary Fig. 16 (A) Molar ratio between $OSO_2CF_3^-$ (blue triangles) and PF_6^- (red circles) in the nanotube crystals. (B) ^{19}F NMR spectral changes during the anion exchange experiments.

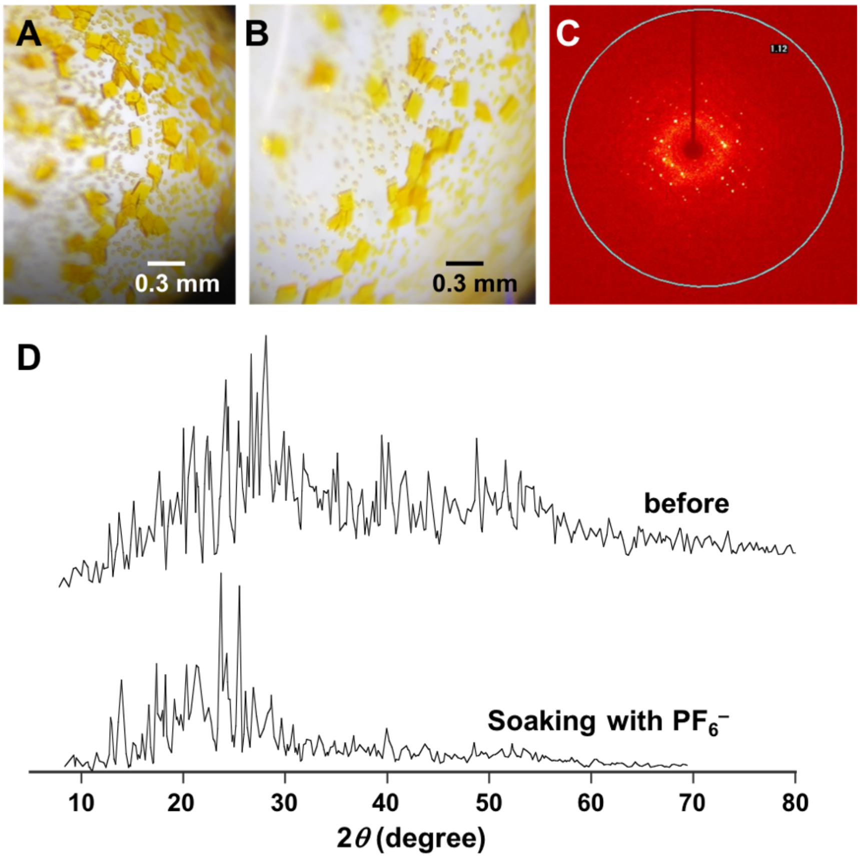

Supplementary Fig. 17 (A,B) Photograph of crystals of $[(Zn^{2+})_4(L_A)_4(L_{A=O})_4]_n(OSO_2CF_3^-)_{8n}$ (A) before, and (B) after the anion exchange experiment with $NBu_4{\cdot}PF_6$. (C) Photograph of X-ray diffraction

image of the PF_6^--soaking crystal after the anion exchange experiment. (D) X-ray diffraction patterns before and after the anion exchange experiment. *The amount of the PF_6^--soaking crystals is too small to perform powder XRD.

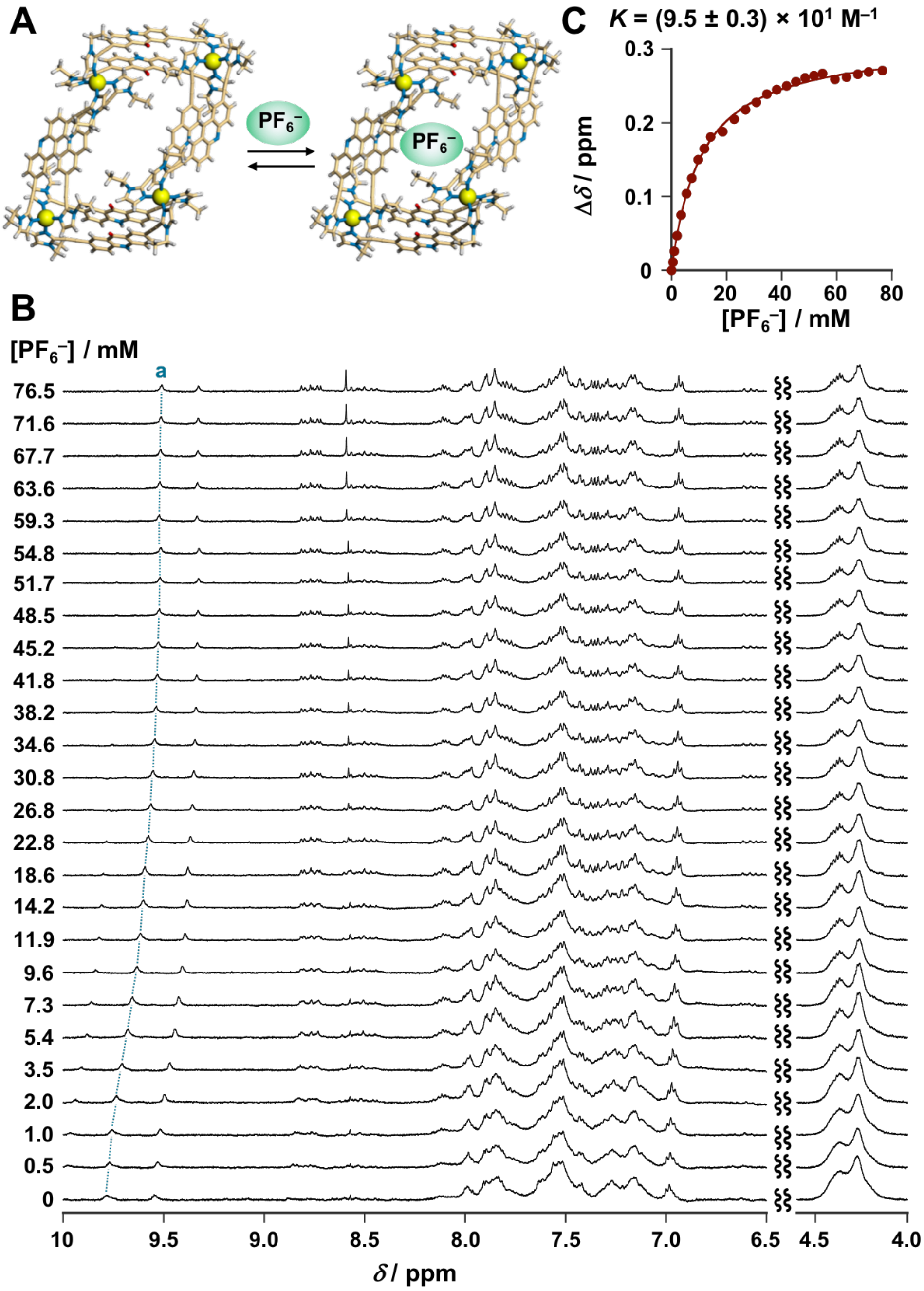

Supplementary Fig. 18 (A) Schematic representation for host-guest complex formation between the heteroleptic Zn_4-metallocycle and PF_6^-. (B) Stacked ^1H NMR spectra of the heteloreptic Zn_4-metallocycle (0.50 mM) in the presence of $NBu_4{\cdot}PF_6$ (0–76.5 mM) in CD_3CN at 298 K. (C) Plot of chemical shift (H^a) versus $[PF_6^-]$.

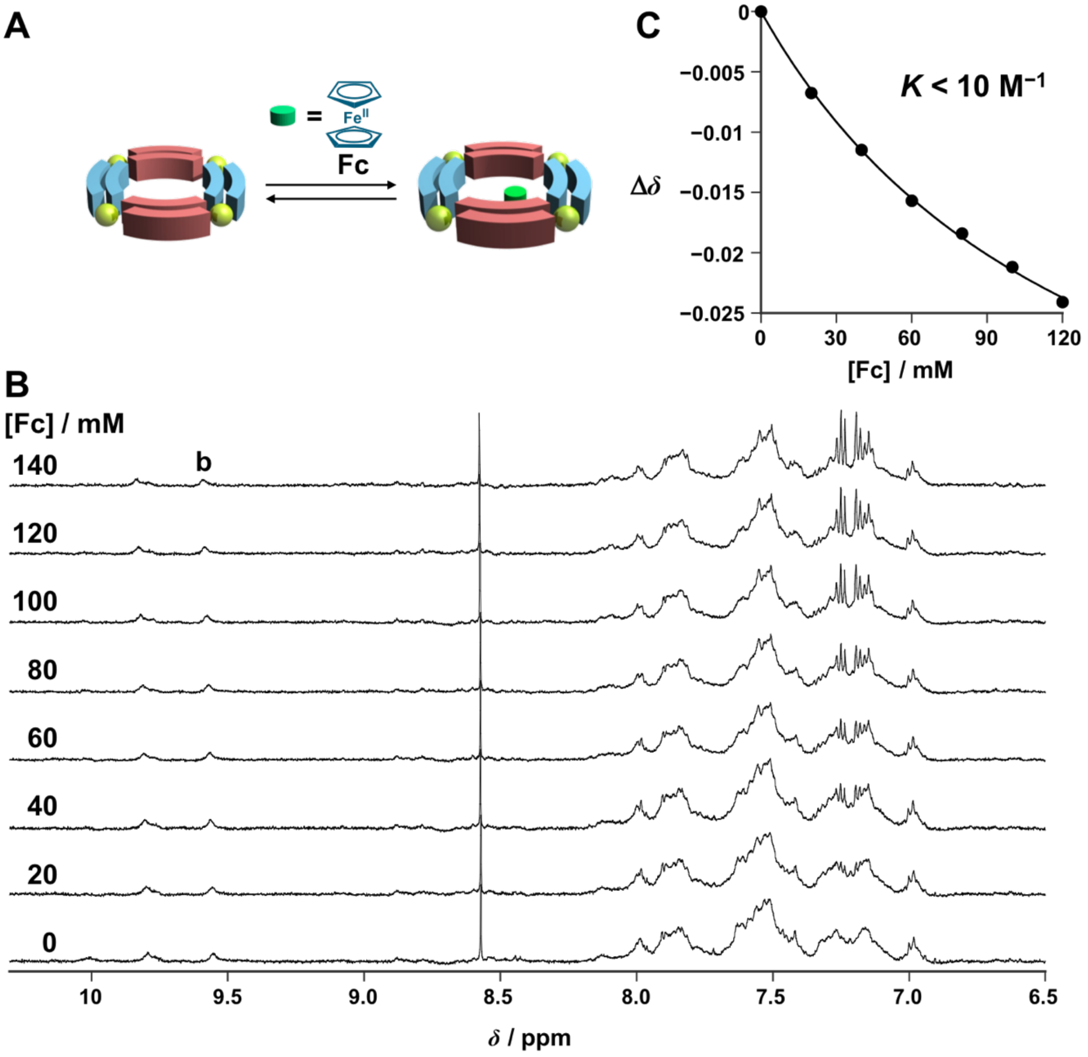

Supplementary Fig. 19 (A) Schematic representation for host-guest complex formation between the heteroleptic Zn_4-metallocycle and Fc. (B) Stacked ^{1}H NMR spectra of the heteroleptic Zn_4-metallocycle (0.50 mM) in the presence of Fc (0–140 mM) in CD_3CN at 298 K. (C) Plot of chemical shift (H^b) versus concentration of [Fc]. Solid line shows the theoretical fitting curve based on the 1:1 host-guest complex formation.

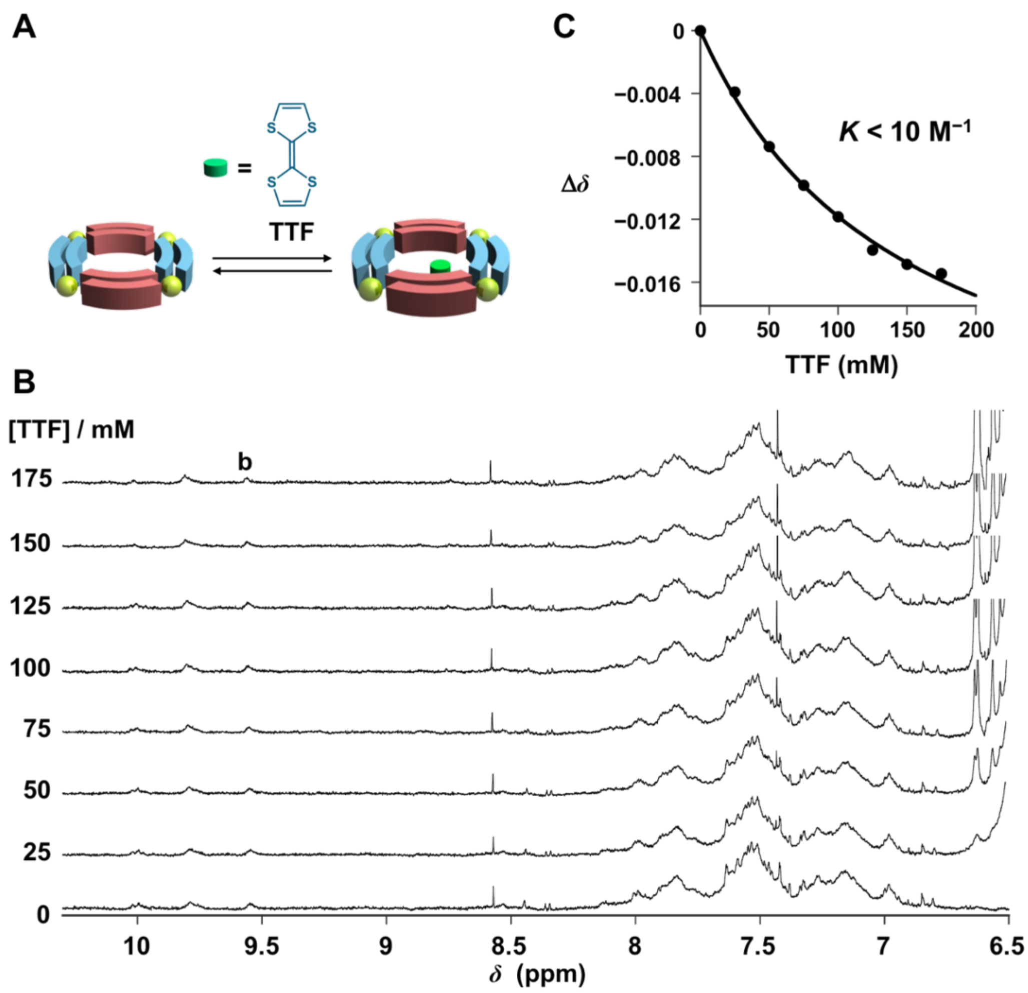

Supplementary Fig. 20 (A) Schematic representation for host-guest complex formation between the heteroleptic Zn_4-metallocycle and TTF. (B) Stacked ^{1}H NMR spectra of the heteroleptic Zn_4-metallocycle (0.50 mM) in the presence of TTF (0–175 mM) in CD_3CN at 298 K. (C) Plot of chemical shift (H^b) versus concentration of [TTF]. Solid line shows the theoretical fitting curve based on the 1:1 host-guest complex formation.

Supplementary Note 6. Absorption of ferrocene and tetrathiafulvalene in the nanotube crystals. In particular, 0.5 mg of the double-wall nanotube crystals ($[(Zn^{2+})_4(L_A)_4(L_{A=O})_4]_n$) were placed into the glass sample tube containing acetonitrile/1,4-doxane mixed solution (1/2, vol/vol, 0.8 mL) dissolving ferrocene ([Fc] = 0.35 M). After 7 days, color of the Fc-soaking crystals were changed from light yellow to dark yellow, while the nanotube crystals maintained their shape during the soaking experiments (Supplementary Fig. 22). The average occupancy of Fc in the nanotube crystal was determined to be 84% (meaning 0.84 Fc molecule absorbed per one Zn_4-host frame on average) from the electron density ($F_o - F_c$) of the Fc guests in the X-ray structure

analysis of the Fc-soaking crystal. Conversely, the Zn_4-host frame can absorb maximum two Fc molecules (i.e., $[(Fc)_2 \subset (Zn^{2+})_4(L_A)_4(L_{A=O})_4]_n$). The low occupancy of the Fc guest in the nanotube crystal was caused by less effective stacking interactions with the inner acridine rings (Supplementary Fig. 25). Besides, the absorption of Fc in the nanotube crystals was independently confirmed by ^{1}H NMR spectroscopy: The Fc-soaking crystals were washed with acetonitrile/1,4-doxane mixed solution (1/2, vol/vol, 0.8 mL) and then dried in a vacuum prior to the NMR measurement. Then, the resulting Fc-soaking crystals were dissolved in DMF-d_7 and the ^{1}H NMR spectrum was measured (Supplementary Fig. 23). It is worth noting that the average occupancy value estimated by the ^{1}H NMR spectroscopy should be underestimated, since the Fc guest molecules absorbed in the nanotube crystals largely removed by the surface washing and drying (in a vacuum) processes prior to the NMR measurement. As expected, the average occupancy of Fc in the nanotube crystal was underestimated to be 24% (meaning 0.24 Fc molecule absorbed per one Zn_4-host frame on average), which was obtained by the relative integration of ^{1}H NMR peaks due to Fc and those due to L_A and $L_{A=O}$ (Supplementary Fig. 23).

In the same manner, TTF-soaking crystals were obtained and analyzed by X-ray crystallography, where the average occupancy of TTF in the nanotube crystal was determined to be 126% (meaning 1.26 TTF molecules absorbed per one Zn_4-host frame on average) from the electron density ($F_o - F_c$) of the TTF guests in the X-ray crystal structure. The absorption of TTF in the nanotube crystals was also confirmed by cyclic voltammetry (CV): The TTF-soaking crystals were washed with acetonitrile/1,4-doxane (1/2, vol/vol, 0.8 mL) and dried in a vacuum. Then, the resulting crystals were dissolved in deaerated acetonitrile (263 μg/0.25 mL) and the CV was measured, where the reversible redox wave due to the TTF redox cycle was successfully observed (Supplementary Fig. 24). The average occupancy of TTF in the nanotube crystals was estimated to be 50% (meaning 0.5 TTF molecule absorbed per one Zn_4-host frame on average) from the observed current value of the TTF redox cycle by reference to that of standard TTF sample (Supplementary Fig. 24). In this case too, the average occupancy (50%) determined from the CV measurement was underestimated, since the TTF guest molecules absorbed in the nanotube crystals were partially removed by the surface washing and drying processes prior to the CV measurement.

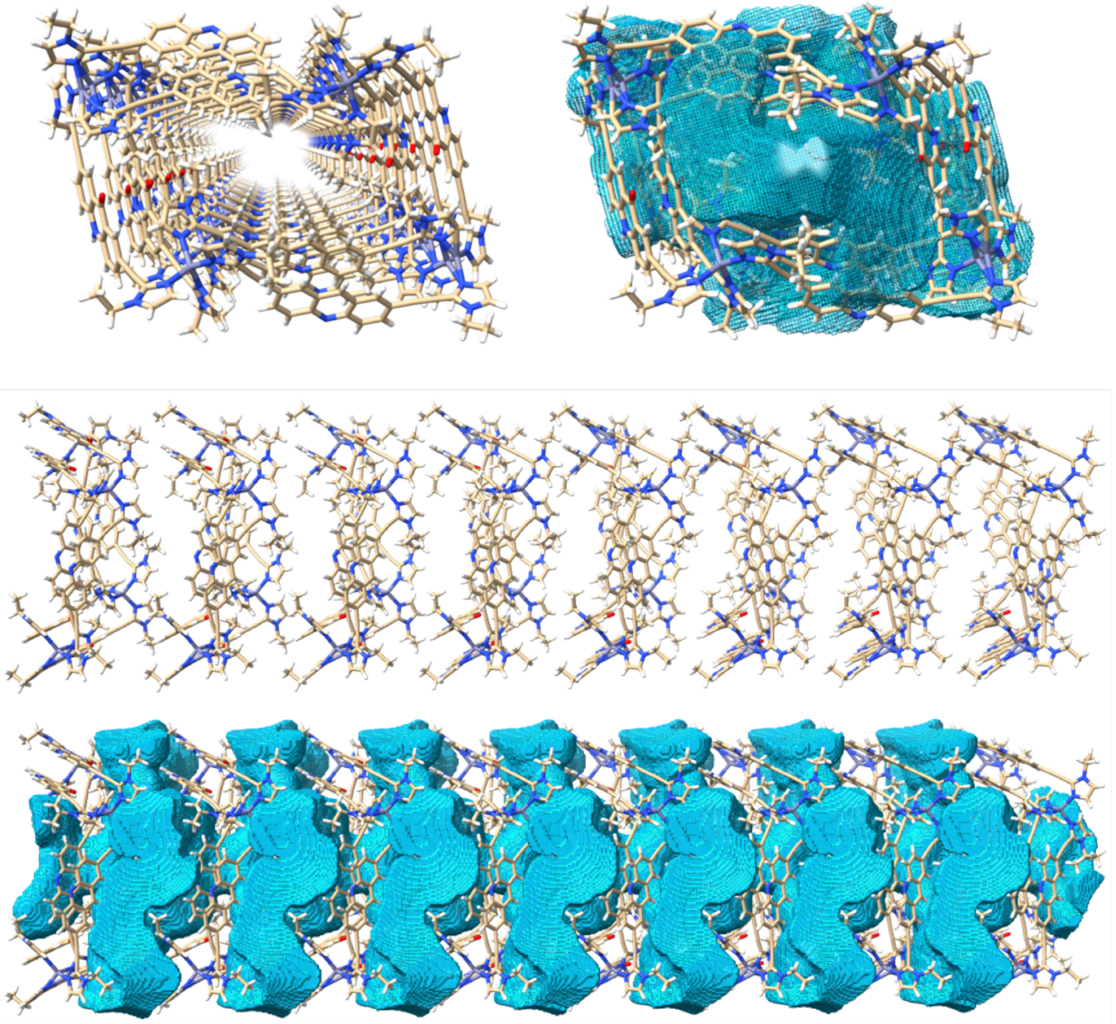

Tabel. Cavity data

**Cavity volume**	**21609.4 Å^3**
**Cavity type**	**Tunnel**

Tabel. Cavity calculation parameters

Probe mode	Two probes
Small probe radius	1.83 Å
Large probe radius	10 Å
Grid resolution	0.1 Å
Optimization depth	4

Supplementary Fig. 21 Visualization of cavity volume (blue) in $[(Zn^{2+})_4(L_A)_4(L_{A=O})_4]_n$. Cavity volume data of $[(Zn^{2+})_4(L_A)_4(L_{A=O})_4]_n$. Cavity volume was calculated with MoloVol 1.0.0 based on the $[(Zn^{2+})_4(L_A)_4(L_{A=O})_4]_n$ crystal structures.[9]

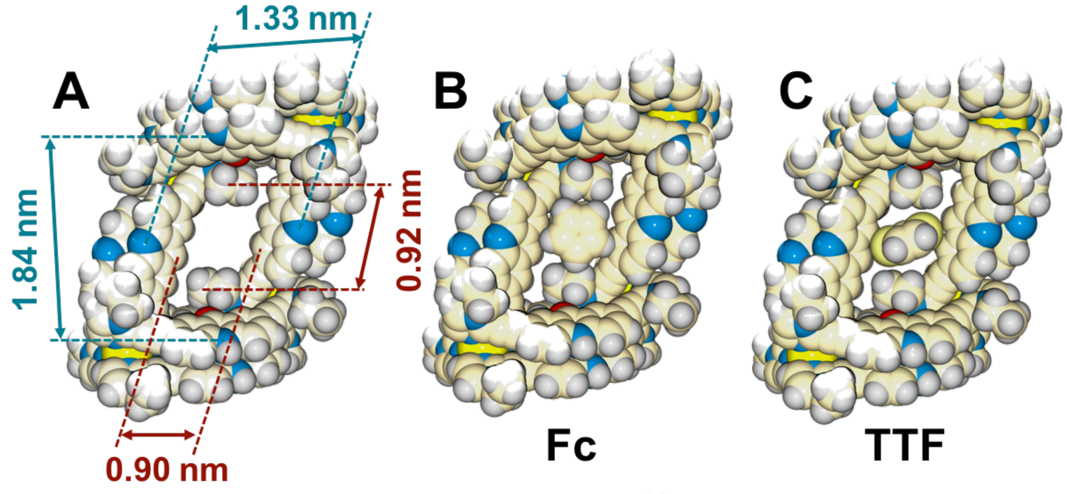

Supplementary Fig. 22 (A) Definition of window size of $[(Zn^{2+})_4(L_A)_4(L_{A=O})_4]_n$. Red arrows: smaller window. Blue arrows: larger window. (B and C) Proposed docking model structures upon passing (B) Fc, and (C) TTF through the $(Zn^{2+})_4(L_A)_4(L_{A=O})_4$ host frame. Here, the X-ray crystal structure of $(Zn^{2+})_4(L_A)_4(L_{A=O})_4$ was used as the structures of the host frame, and DFT-optimized structures ([B3LYP/6-31G+(d,p) [C H N O S]; LANL2DZ (Zn Fe)]) were used as the guest structures.

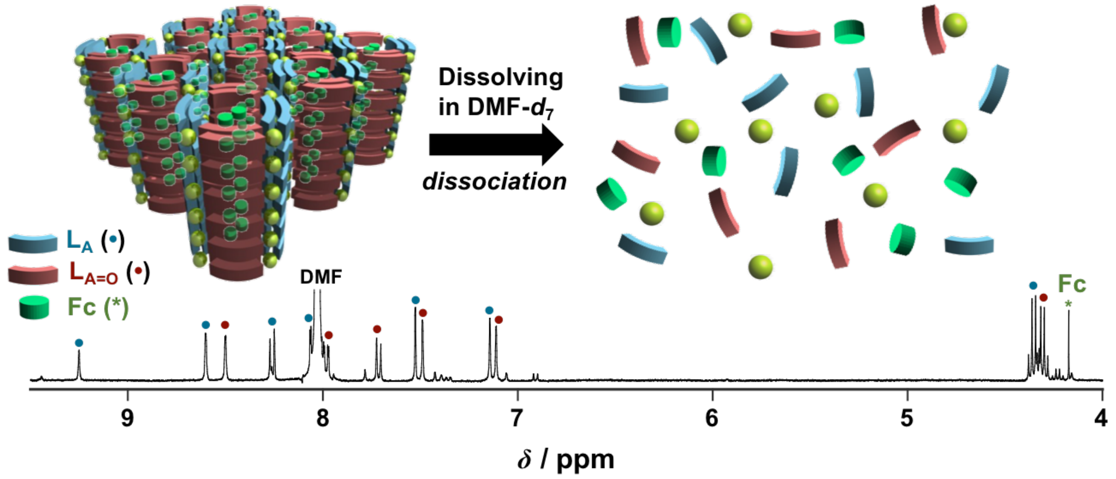

Supplementary Fig. 23 ^{1}H NMR spectrum of the Fc-soaking crystals dissolving in DMF-d_7.

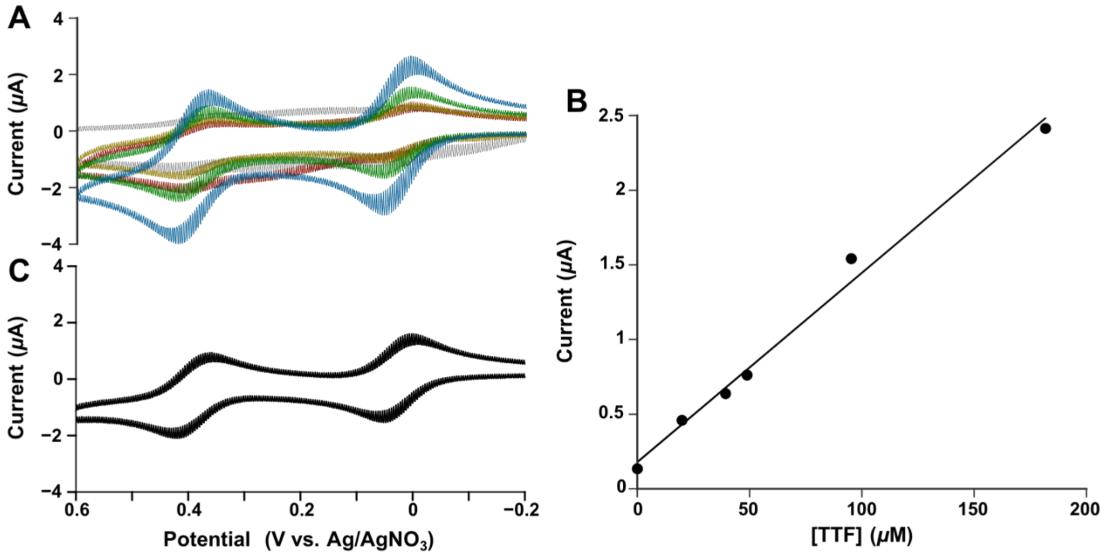

Supplementary Fig. 24 (A) Cyclic voltammogram of TTF (gray line: 19 μM, red line: 39 μM, yellow line: 49 μM, green line: 95 μM, and blue line: 180 μM) in deaerated acetonitrile containing 0.1 M TBAP. (B) Plot of current versus [TTF]. (C) Cyclic voltammogram of the TTF-soaking crystals of $[(Zn^{2+})_4(L_A)_4(L_{A=O})_4]_n$ upon dissolving in deaerated acetonitrile containing 0.1 M TBAP. Since the amount of the crystal sample (TTF-soaking crystal) is quite small, the specific electrochemical cell (SVC-2 Voltammetry cell/ Sample holder dia 9 mmϕ) was used.

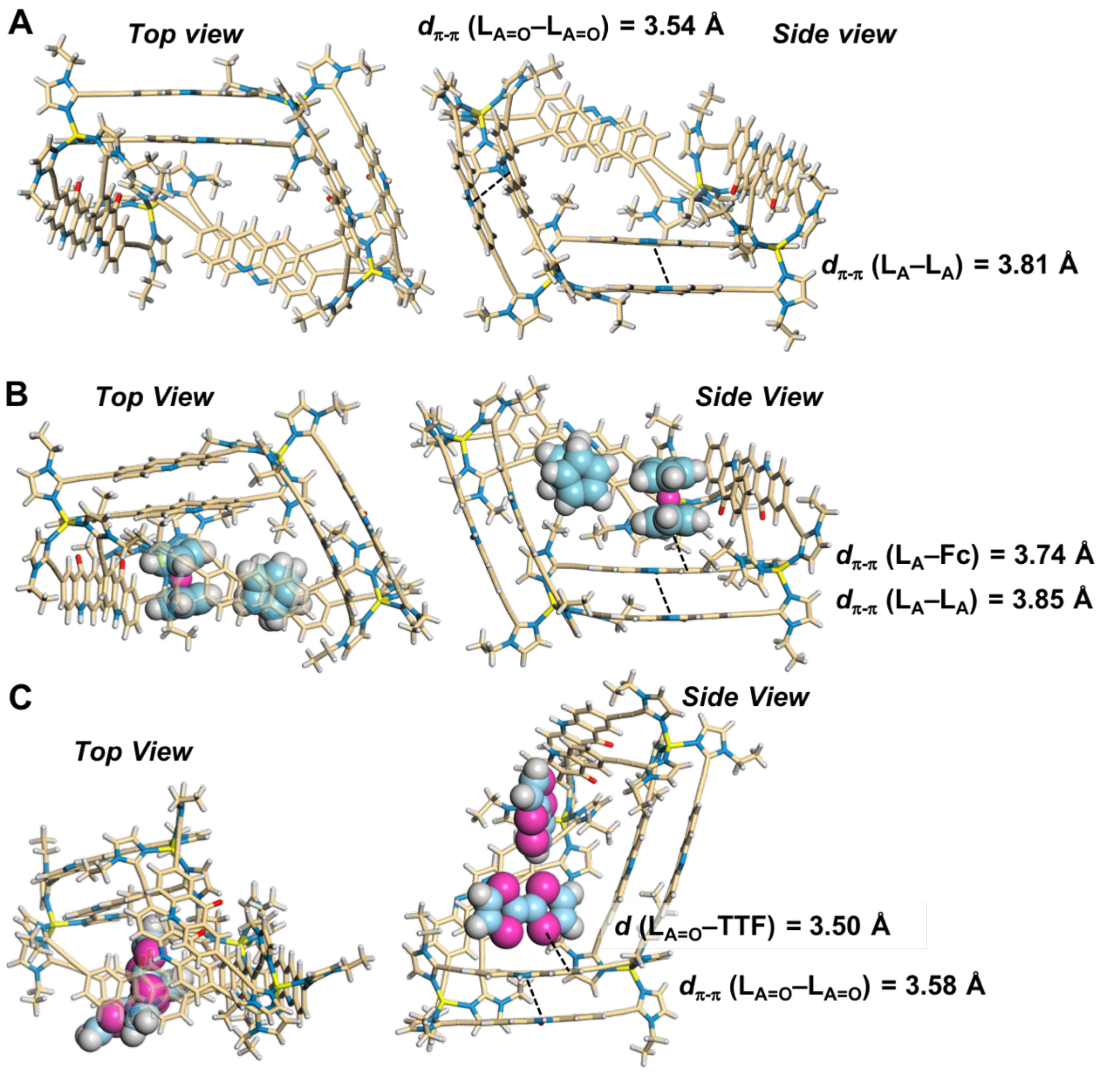

Supplementary Fig. 25 Suggested interactions in (A) $(Zn^{2+})_4(L_A)_4(L_{A=O})_4$, (B) $(Fc)_2\subset(Zn^{2+})_4(L_A)_4(L_{A=O})_4$, and (C) $(TTF)_2\subset(Zn^{2+})_4(L_A)_4(L_{A=O})_4$.

We have also performed the double guests (Fc or TTF) incorporation experiment (*vide infra*). The nanotube crystal ($[(Zn^{2+})_4(L_A)_4(L_{A=O})_4]_n$) was immersed in the mixed solution of Fc or TTF in 7 days. The nanotube crystal color was changed from light yellow to dark brown, indicating the incorporation of the guest molecules into the nanotube crystal. Then, we analyzed the Fc/TTF-soaking crystal by X-ray crystallography, where new electron density was appeared inside the $(Zn^{2+})_4(L_A)_4(L_{A=O})_4$ host frame. However, the appeared new electron density could not be defined well probably due to statistical incorporation of Fc and TTF into the nanotube channel.

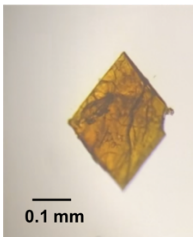

Supplementary Fig. 26 Photograph of double-wall nanotube crystal ($[(Zn^{2+})_4(L_A)_4(L_{A=O})_4]_n$) after soaking in solution containing Fc and TTF.

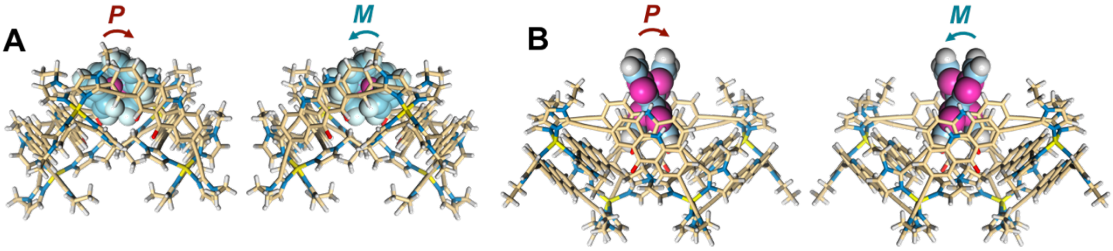

Supplementary Fig. 27 Representation of *P*- and *M*-helicity for the guest molecules in (A) $(Fc)_2 \subset (Zn^{2+})_4(L_A)_4(L_{A=O})_4$ and (B) $(TTF)_2 \subset (Zn^{2+})_4(L_A)_4(L_{A=O})_4$.

Supplementary Note 7. Mechanism for electron-transfer oxidation of the TTF-incorporated nanotube crystal. For the purpose of effective facial ET process, the TTF-incorporated nanotube crystals ($[(TTF)_2 \subset (Zn^{2+})_4(L_A)_4(L_{A=O})_4]_n$) and the enough amount of $[Fe(H_2O)_6](ClO_4)_3$ solids were mixing in poor solvent (hexane/liquid paraffin mixed solution (1/1, vol/vol)) in 1 hour (Supplementary Fig. 30), where the nanotube crystal surface was contacted with the $[Fe(H_2O)_6](ClO_4)_3$ solids many times during the mixing. This resulted in a drastic color change of the TTF-incorporated nanotube crystals from yellow to dark, indicating an effective facial ET oxidation with $[Fe(H_2O)_6](ClO_4)_3$. The X-ray crystal structure analysis of the TTF-incorporated nanotube crystals after the ET oxidation revealed oxidation of the Zn_4-host frame ($(Zn^{2+})_4(L_A)_4(L_{A=O})_4$ to $(Zn^{2+})_4(L_{A=O})_8$) (Supplementary Fig. 32) and the ET oxidation of the inner TTF guests. It is noteworthy that the surface oxidation of the Zn_4-host frame was also observed

in the electron-donor unoccupied nanotube crystal (Supplementary Figs. 31 and 34). Thus, the host-frame oxidation occurred independently of the ET oxidation of the inner electron donors. Besides, the surface oxidation of the Zn_4-host frame was also confirmed by ^{1}H NMR spectroscopy (Supplementary Fig. 35). Although it is apparent that the TTF-incorporated nanotube crystal was successfully oxidized through the crystal-to-crystal process, whose mechanism is not clear at present. Conversely, TTF single crystals without the nanotube crystals could not be sufficiently oxidized with $[Fe(H_2O)_6](ClO_4)_3$ solids, where the yellow color due to neutral TTF was not changed upon mixing with $[Fe(H_2O)_6](ClO_4)_3$ solids in hexane/liquid paraffin mixed solution (Supplementary Fig. 42). The negative result obtained with the TTF single crystals indicates that the large tubular void (0.90 nm × 0.92 nm) of the Zn_{4n}-host frame should play an important role in the crystal-to-crystal ET oxidation process, therefore we proposed a hole/anion-deliver mechanism (Supplementary Fig. 43).

The average occupancy of TTF in the nanotube crystal after the ET-oxidation was determined to be 126% (meaning ~1.3 TTF molecules per one Zn_4-host frame on average) from the electron density ($F_o - F_c$) of the TTF guests in the X-ray structure analysis of the TTF-incorporated crystal after the ET oxidation, which unchanged from the original average occupancy of TTF before the ET oxidation (126%, *vide supra*). Conversely, the average occupancy of ClO_4^- appeared after the ET oxidation is almost 100% (meaning two ClO_4^- molecules per one Zn_4-host frame). Hence, the average oxidation state of the inner TTF guests after the ET oxidation was suggested to be ~+1.6. Besides, the TTF-incorporated crystals after the ET oxidation exhibited an intense ESR signal due to $TTF^{\bullet+}$ (Supplementary Fig. 29), indicating that the Zn_{4n}-host frame contains the mixture of $TTF^{\bullet+}$ and TTF^{2+}.

In the same manner, the facial ET oxidation of the Fc-incorporated nanotube crystals($[(Fc)_2 \subset (Zn^{2+})_4(L_A)_4(L_{A=O})_4]_n$) was performed with the $[Fe(H_2O)_6](ClO_4)_3$ solids. The average occupancy of Fc in the nanotube crystal after the ET-oxidation was determined to be 53% (meaning ~1.1 Fc molecules per one Zn_4-host frame on average) from the electron density ($F_o - F_c$) of the Fc guests in the X-ray structure analysis of the Fc-incorporated crystal after the ET oxidation. In this case, the average occupancy of ClO_4^- appeared after the ET oxidation is the same as the average occupancy of Fc 53% (meaning 1.1 ClO_4^- molecules per one Zn_4-host frame). Thus, the inner Fc guests were fully oxidized to Fc^+ by the solid-state ET oxidation.

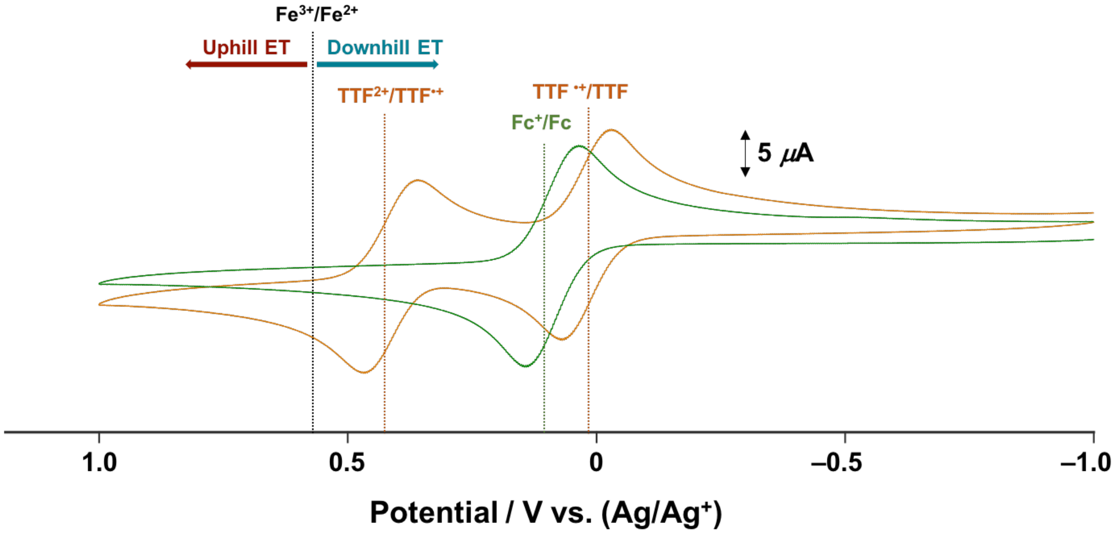

Supplementary Fig. 28 Cyclic voltammogram of Fc (1.0 mM) and TTF (1.0 mM) in deaerated acetonitrile containing 0.1 M $TBAPF_6$. Scan rate: 200 mV/s.

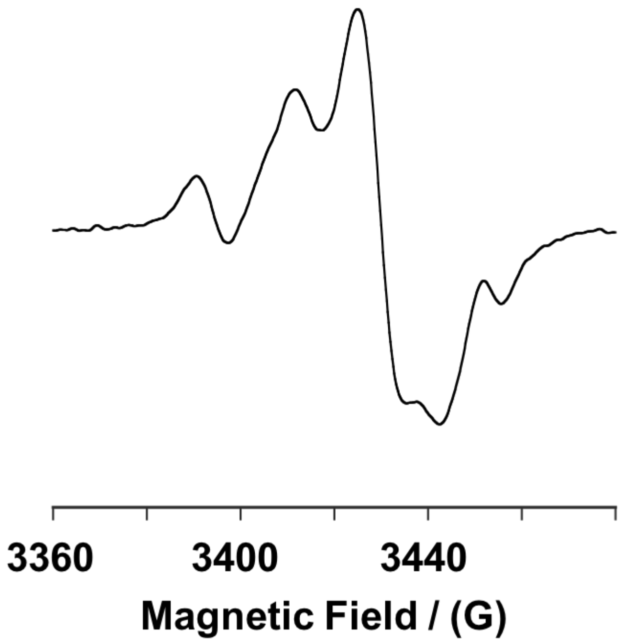

Supplementary Fig. 29 Solid state ESR spectra of the TTF-incorporated nanotube crystals after ET oxidation with $[Fe(H_2O)_6](ClO_4)_3$ solids.

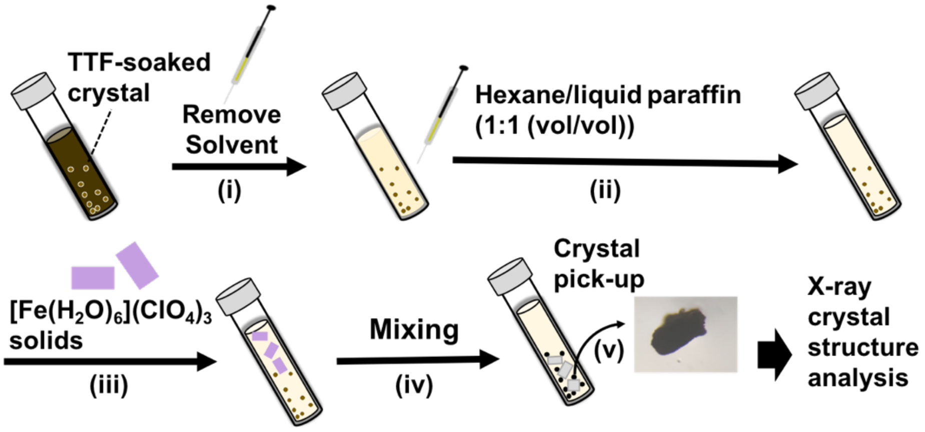

Supplementary Fig. 30 The procedures for ET oxidation of the $[(TTF)_2{\subset}(Zn^{2+})_4(L_A)_4(L_{A=O})_4]_n$ crystals. (i) The crystallization solvent of the TTF-soaked crystal was removed, and (ii) hexane/liquid paraffin mixed solution (1/1, vol/vol) was added. (iii) Then, the $[Fe(H_2O)_6](ClO_4)_3$ solids were added in the resulting solution, (iv) and the reaction mixture was mixing in 1 hour. Then, the crystal color was changed from yellow to dark. (v) The crystal suitable for X-ray crystallography was picked up.

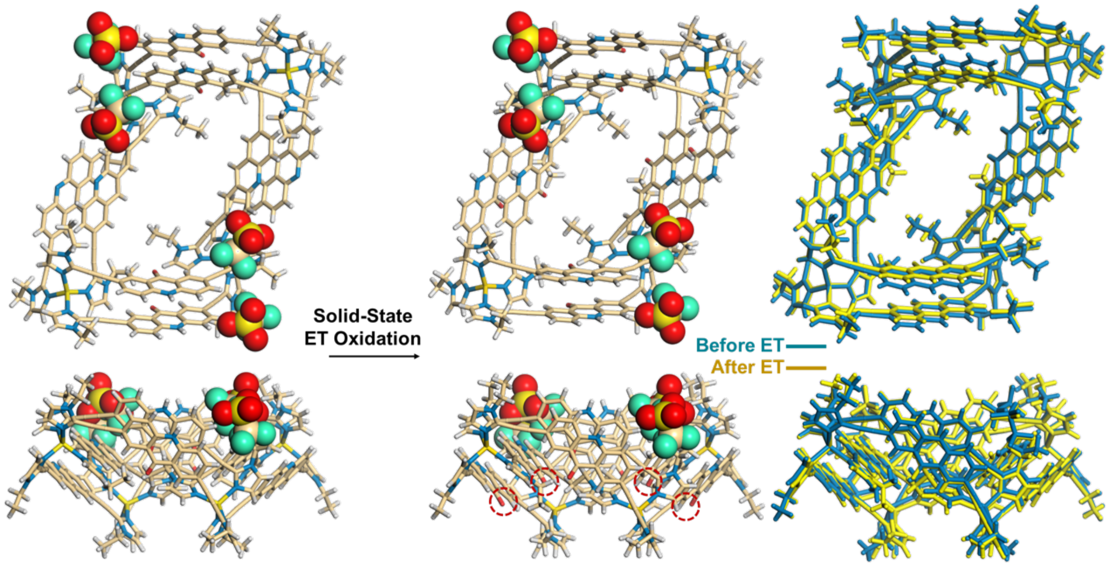

Supplementary Fig. 31 X-ray crystal structures of $[(Zn^{2+})_4(L_A)_4(L_{A=O})_4]_n$ before and after ET oxidation with $[Fe(H_2O)_6](ClO_4)_3$ solids. One of the disordered structures were shown for clarity. Dashed circles indicate newly appeared oxygen atoms after the solid-state ET oxidation.

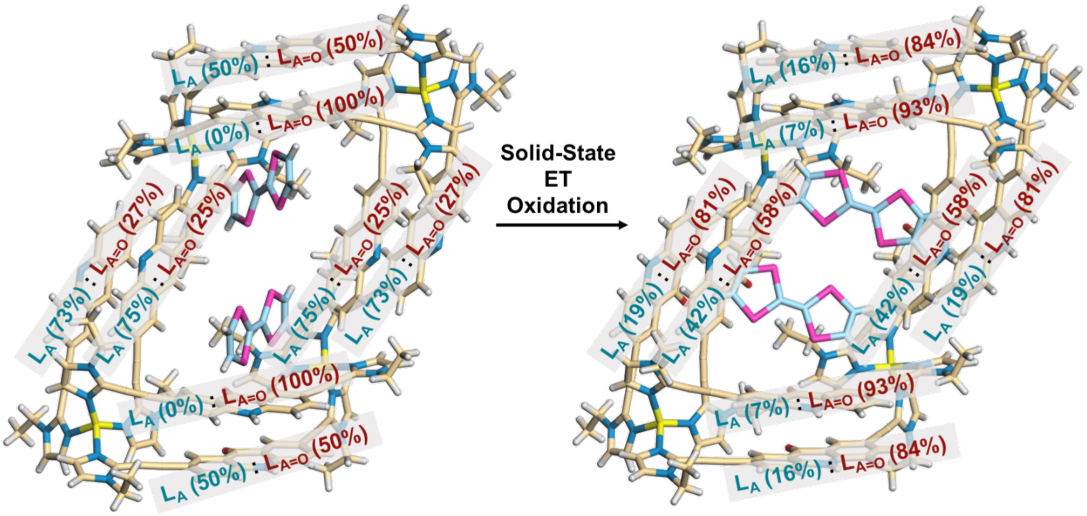

Supplementary Fig. 32 Ratio between L_A and $L_{A=O}$ in the Zn_4-host frame in $(TTF)_2\subset(Zn^{2+})_4(L_A)_4(L_{A=O})_4$ before (left) and after (right) the ET oxidation with $[Fe(H_2O)_6](ClO_4)_3$ solids determined by the electron density ($F_o - F_c$) analysis of the oxygen atoms of the 9-positions of the acridone rings.

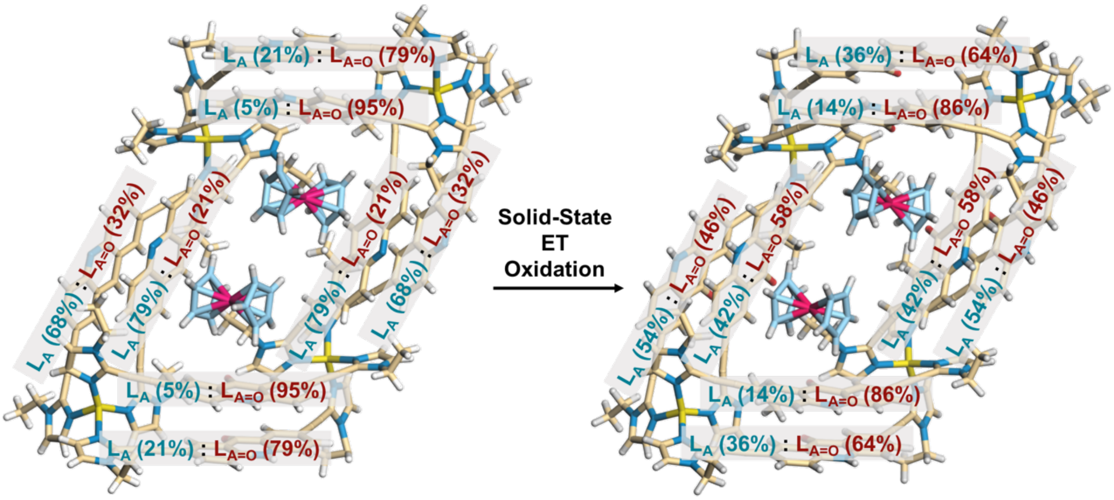

Supplementary Fig. 33 Ratio between L_A and $L_{A=O}$ in the Zn_4-host frame in $(Fc)_2\subset(Zn^{2+})_4(L_A)_4(L_{A=O})_4$ before (left) and after (right) the ET oxidation with $[Fe(H_2O)_6](ClO_4)_3$ solids determined by the electron density ($F_o - F_c$) analysis of the oxygen atoms of the 9-positions of the acridone rings.

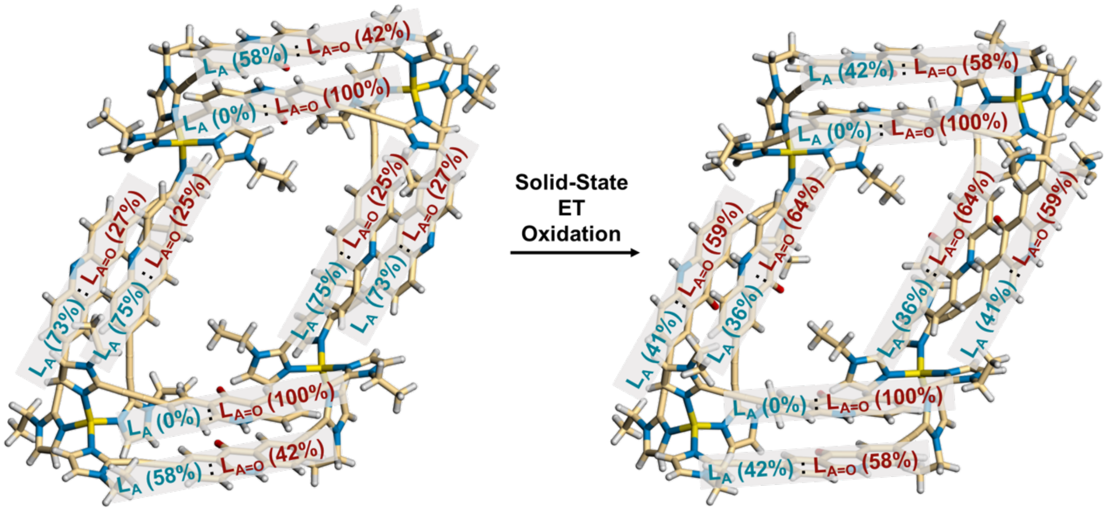

Supplementary Fig. 34 Ratio between L_A and $L_{A=O}$ in the Zn_4-host frame in $(Zn^{2+})_4(L_A)_4(L_{A=O})_4$ before (left) and after (right) the ET oxidation with $[Fe(H_2O)_6](ClO_4)_3$ solids determined by the electron density ($F_o - F_c$) analysis of the oxygen atoms of the 9-positions of the acridone rings.

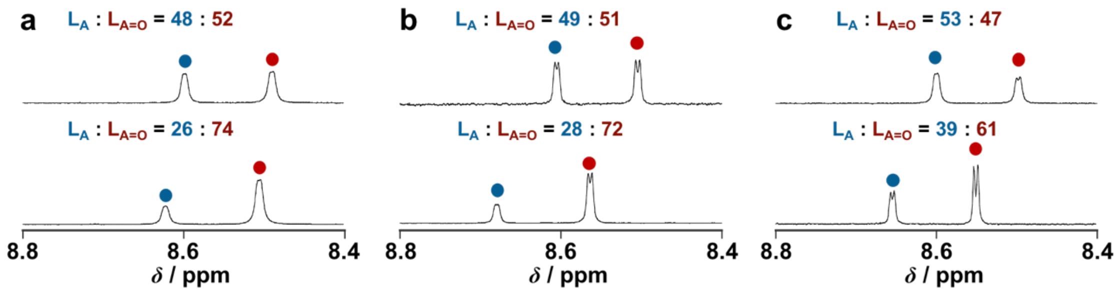

Supplementary Fig. 35 Partial ^1H NMR spectra (dissolving in DMF-d_7) of (a) $[(Zn^{2+})_4(L_A)_4(L_{A=O})_4]_n$, (b) $[(TTF)_2{\subset}(Zn^{2+})_4(L_A)_4(L_{A=O})_4]_n$, and (c) $[(Fc)_2{\subset}(Zn^{2+})_4(L_A)_4(L_{A=O})_4]_n$ crystals before (top) and after (bottom) the ET oxidation with $[Fe(H_2O)_6](ClO_4)_3$ solids. Blue and red circles denote the ^1H NMR signals due to L_A and $L_{A=O}$, respectively.

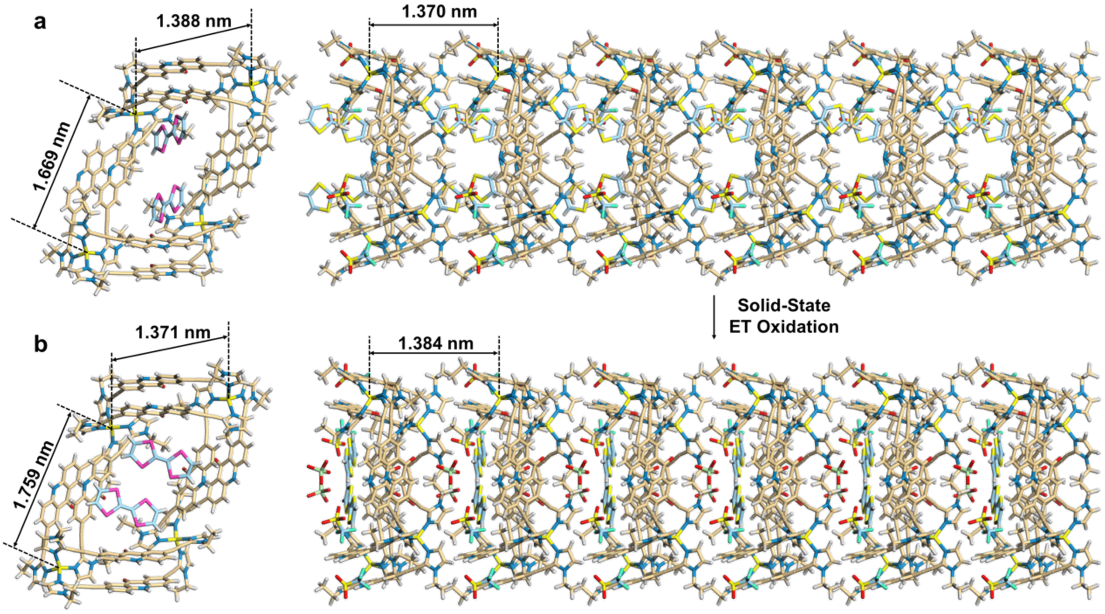

Supplementary Fig. 36 Selected Zn–Zn distance of the Zn_4-host frame in $(TTF)_2\subset(Zn^{2+})_4(L_A)_4(L_{A=O})_4$ before (a) and after (b) the ET oxidation with $[Fe(H_2O)_6](ClO_4)_3$ solids. Left figures: top views (counter anions were omitted for clarity); right figures: side views.

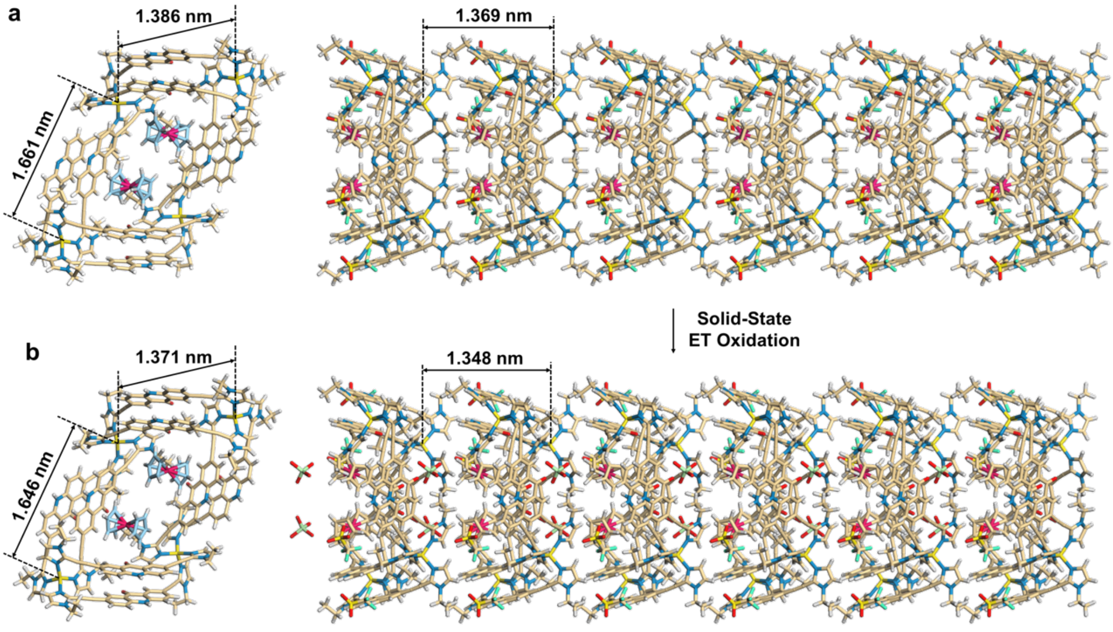

Supplementary Fig. 37 Selected Zn–Zn distance of the Zn_4-host frame in $(Fc)_2\subset(Zn^{2+})_4(L_A)_4(L_{A=O})_4$ before (a) and after (b) the ET oxidation with $[Fe(H_2O)_6](ClO_4)_3$ solids. Left figures: top views (counter anions were omitted for clarity); right figures: side views.

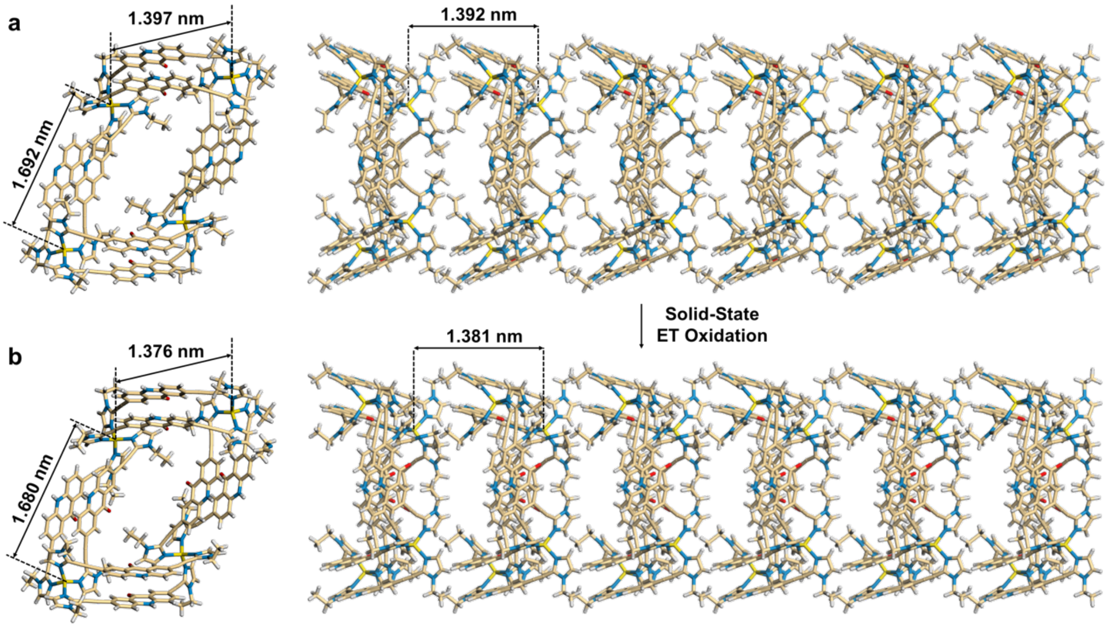

Supplementary Fig. 38 Selected Zn–Zn distance of the Zn_4-host frame in $(Zn^{2+})_4(L_A)_4(L_{A=O})_4$ before (a) and after (b) the ET oxidation with $[Fe(H_2O)_6](ClO_4)_3$ solids. Left figures: top views; right figures: side views. Counter anions were omitted for clarity.

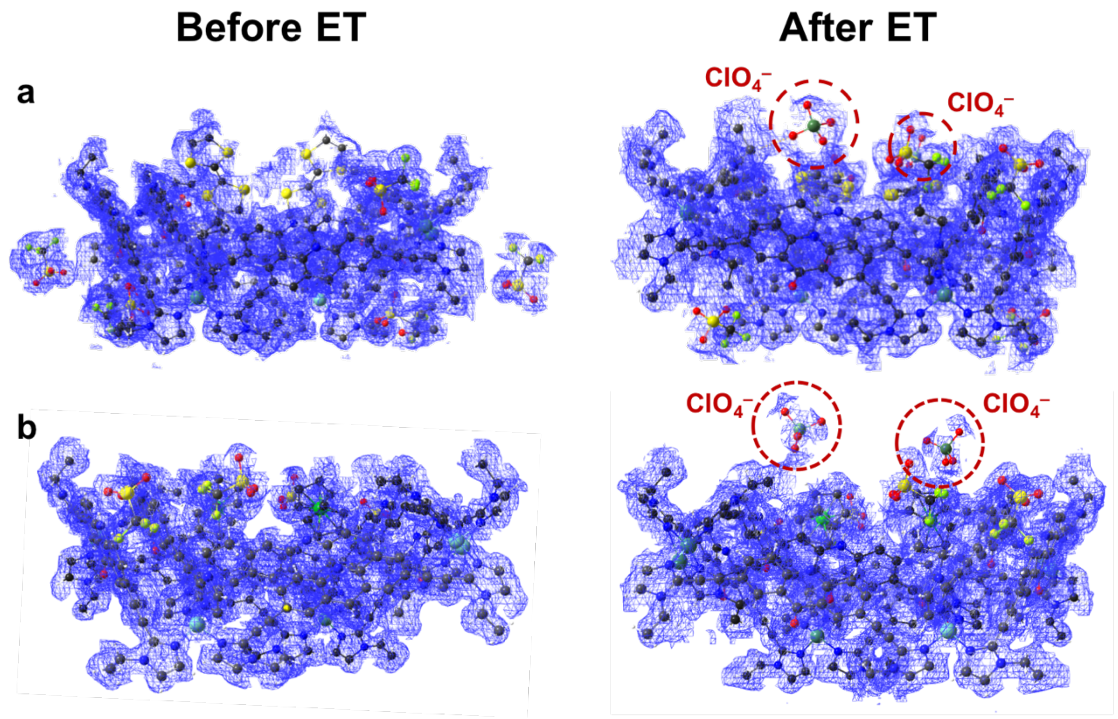

Supplementary Fig. 39 Electron density map (F_o) of **a**, $[(TTF)_2{\subset}(Zn^{2+})_4(L_A)_4(L_{A=O})_4]_n$ and **b**, $[(Fc)_2{\subset}(Zn^{2+})_4(L_A)_4(L_{A=O})_4]_n$ (left) before and (right) after ET oxidation with $[Fe(H_2O)_6](ClO_4)_3$ solids.

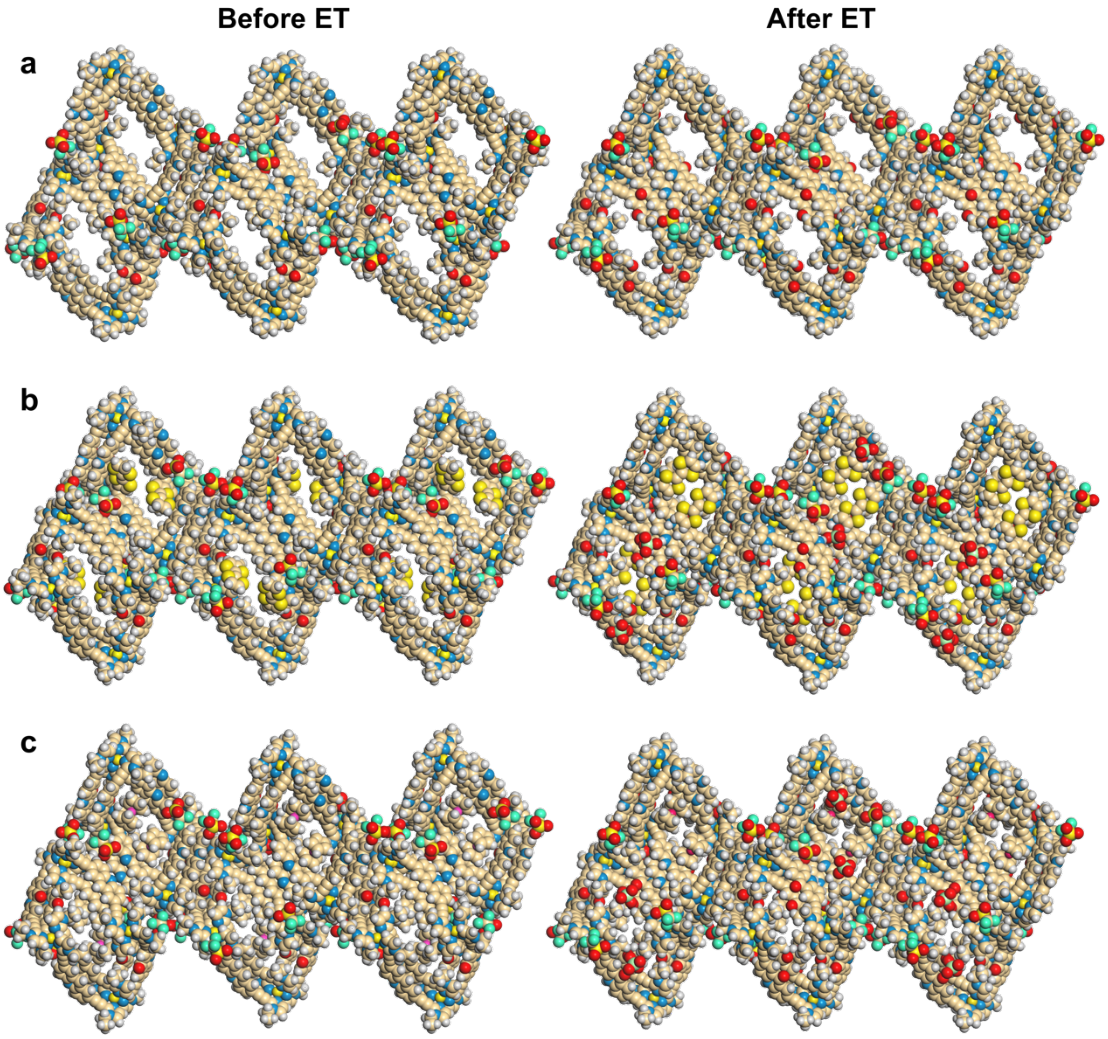

Supplementary Fig. 40 Crystal packing diagram of **a**, $[(Zn^{2+})_4(L_A)_4(L_{A=O})_4]_n$, **b**, $[(TTF)_2\subset(Zn^{2+})_4(L_A)_4(L_{A=O})_4]_n$, and **c**, $[(Fc)_2\subset(Zn^{2+})_4(L_A)_4(L_{A=O})_4]_n$ (left) before and (right) after ET oxidation with $[Fe(H_2O)_6](ClO_4)_3$ solids.

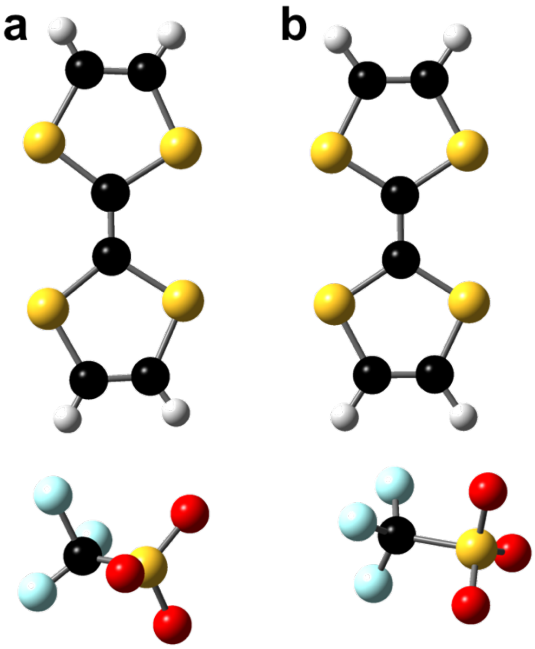

Supplementary Fig. 41 a, Core structure of the OTf···TTF unit in the X-ray crystal structure of $[(TTF)_2 \subset (Zn^{2+})_4(L_A)_4(L_{A=O})_4]_n$ after the solid-state ET oxidation. **b**, Structure of the hydrogen-bonded complex between OTf^- and $TTF^{\bullet+}$ optimized with DFT [CAM-UB3LYP/6-31G(d)].

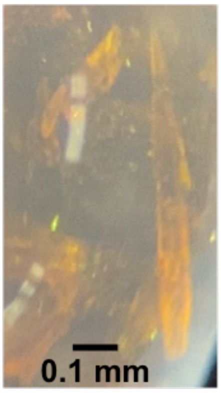

Supplementary Fig. 42 Photograph of TTF crystal after 1 hour mixing with $[Fe(H_2O)_6](ClO_4)_3$ solids in hexane/liquid paraffin mixed solution (1/1, vol/vol).

The appeared liquid interface between the two solids may be originated from the hygroscopic nature of $Fe(ClO_4)_3$ and the solvent molecules (e.g., 1,4-dioxane, diethyl ether, and acetonitrile) originally existing inside the nanotube crystal (see below).

It is worth noting that the size of $[Fe(H_2O)_6]^{3+}$ is too large to pass the window of the $(Zn^{2+})_4(L_A)_4(L_{A=O})_4$ host frame (0.90 nm × 0.92 nm) [Supplementary Fig. 44a]. Moreover, the incorporation of the positively charged $[Fe(H_2O)_6]^{3+}$ into the positively charged $[(Zn)_4(L_A)_4(L_{A=O})_4]^{8+}$ host frame is unlikely to occur. Thus, the (initial) electro-transfer oxidation of the inner electron-donor guests (G) by $[Fe(H_2O)_6]^{3+}$ occurred at the crystal surface (outside the nanotube channel) contacted with the $[Fe(H_2O)_6](ClO_4)_3$ solid (Supplementary Fig. 43(i)). Subsequent hole transfer along the nanotube channel and penetration of the ClO_4^- molecules (additional counter anion) are necessarily to complete the electron-transfer oxidation of the inner electro-donor guests in the whole range (Supplementary Fig. 43(ii) and (iii)). The size of ClO_4^- is small enough to pass the host-frame window (0.90 nm × 0.92 nm) [Supplementary Fig. S44b], and the incorporation of the negatively charged ClO_4^- into the positively charged $[(Zn)_4(L_{A=O})_8]^{8+}$ host frame is likely to occur. Indeed, the additional ClO_4^- molecules were appeared inside the nanotube channel after the electron-donor oxidation, which were directly determined by X-ray crystallography (Fig. 3e). The penetration of ClO_4^- inside the nanotube channels should result in removal of the inner solvent molecules outside the nanotube crystal (Supplementary Fig. 43(i)), causing the appearance of liquid interface between the two solids. In addition, the liquid interface was also appeared in the later period, which may be originated from the hygroscopic nature of the $[Fe(H_2O)_6](ClO_4)_3$ solid. Overall, the appeared liquid interface may work as lubricant to facilitate transport of ClO_4^- into the nanotube channels, assisting the completion of the electron-transfer reaction.

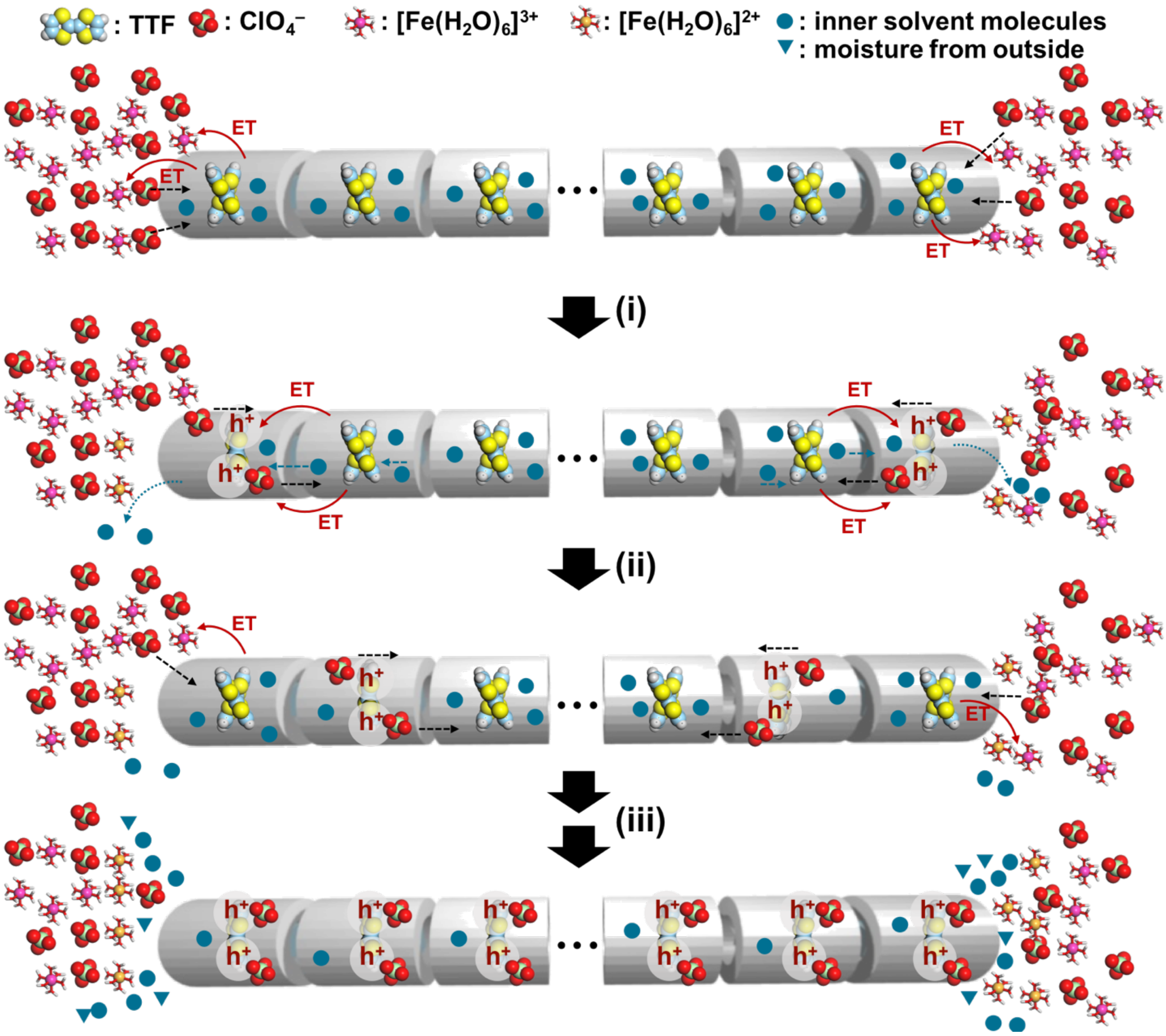

Supplementary Fig. 43 Schematic representation for the proposed mechanism of the hole accumulation in the $[(TTF)_2{\subset}(Zn^{2+})_4(L_A)_4(L_{A=O})_4]_n$ crystals by facial ET oxidation by $[Fe(H_2O)_6](ClO_4)_3$ solids. (i) ET from the inner TTF molecules to $[Fe(H_2O)_6]^{3+}$ yields $TTF^{\bullet+}$ at the crystal surface contact with the $[Fe(H_2O)_6](ClO_4)_3$ solid. (ii) Hole transfer from the terminal $TTF^{\bullet+}$ to the inner TTF reproduce the neutral TTF at the contact surface. (iii) ET oxidation of the terminal TTF occurs to yield $TTF^{\bullet+}$ at the terminal end again. Cycles of these ET process in association with adsorption of ClO_4^- inside the tube can accumulate hole in the whole range of the nanotube crystals. The penetration of ClO_4^- inside the nanotube channels results in removal of the inner solvent molecules (blue circles) outside the nanotube crystal.

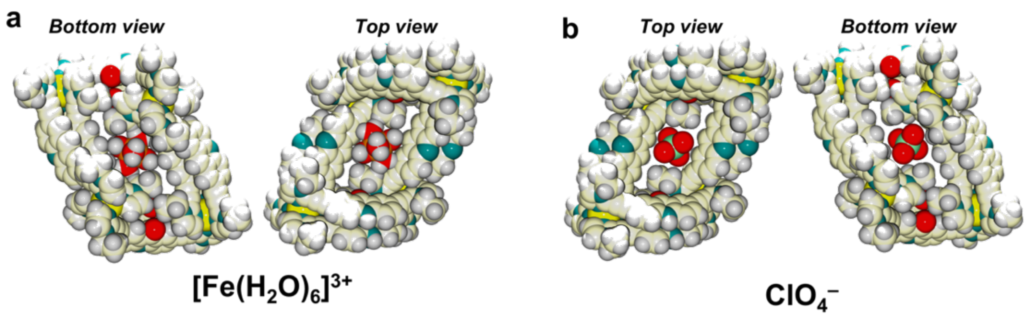

Supplementary Fig. 44 Comparison between window size of the host $(Zn^{2+})_4(L_A)_4(L_{A=O})_4$ frame and (a) $[Fe(H_2O)_6]^{3+}$ and (b) ClO_4^-. Here, the X-ray crystal structure of $(Zn^{2+})_4(L_A)_4(L_{A=O})_4$ was used as the structures of the host frame, and DFT-optimized structures ([CAM-B3LYP/6-31G(d) [H O Cl]; LANL2DZ (Fe)]) were used as the guest structures

We also tested tris(4-bromophenyl)ammoniumyl hexachloroantimonate salts (magic blue) as a solid-state oxidant (without oxygen atoms) in the present system (vide infra). The crystallinity of the nanotube crystal $((Zn^{2+})_4(L_A)_4(L_{A=O})_4]_n)$ was decreased after the surface contact with the magic blue solid (Supplementary Fig. 45a) probably due to the radical nature of magic blue. Hence, we could not resolve the X-ray crystal structure due to the bad quality X-ray data. Conversely, the ratio between L_A and $L_{A=O}$ was determined by ^1H NMR spectroscopy of the resulting crystals dissolved in DMF-d_7 (Supplementary Fig. 45b), indicating that the molar ratio ($[L_A]$:$[L_{A=O}]$ = 1:1) almost unchanged even after the surface contact with the magic blue. Thus, the host frame $((Zn^{2+})_4(L_A)_4(L_{A=O})_4)$ could be maintain as original form by using magic blue solids.

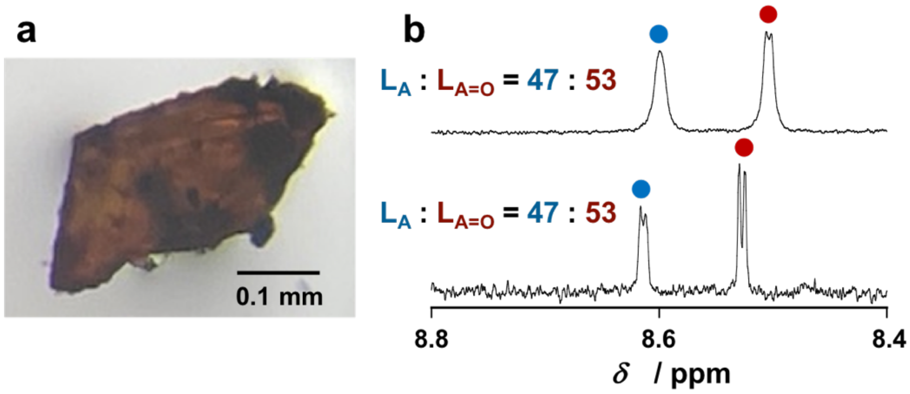

Supplementary Fig. 45 (a) Crystal photograph of the $[(Zn^{2+})_4(L_A)_4(L_{A=O})_4]_n$ crystal after surface contact with the tris(4-bromophenyl)ammoniumyl hexachloroantimonate solids. (b) Partial ^1H NMR spectra (dissolving in DMF-d_7) of $[(Zn^{2+})_4(L_A)_4(L_{A=O})_4]_n$ crystals before (top) and after

(bottom) the ET oxidation with tris(4-bromophenyl)ammoniumyl hexachloroantimonate solids. Blue and red circles denote the ^{1}H NMR signals due to L_A and $L_{A=O}$, respectively.

Our present system is different from the redox-responsive metal-organic-frameworks (MOF) and their crystal-to-crystal transition. (MOF) and their crystal-to-crystal transition in the following points.

(i) The redox-driven crystal-to-crystal transition of MOF relies on the electron-transfer (ET) oxidation of the *host frame* (i.e., redox-active host ligands) [Supplementary Fig. 46a].[10-12] Conversely, our manuscript reports the crystal-to-crystal process of the electron-transfer oxidation of the *guests* (non-derivatized TTF and Fc) *non-covalently incorporated* into the nanotube channels (Supplementary Fig. 46b). Thus, our non-derivatized free system is highly versatile for direct observation of solid-state electron-transfer process of the electron donor molecule itself by X-ray crystallography.

(ii) In addition, the oxidation of MOF was mostly used solution (or vapor) of iodine (I_2).[10-12] In contrast, we used the $[Fe(H_2O)_6](ClO_4)_3$ solid to oxidize the nanotube crystal.

(iii) Furthermore, in the redox-driven crystal-to-crystal transition of MOF, the oxidant of I_2 (dissolved in solution) was most probably directly incorporated into the host-frame channels.[10-12] Subsequently, electron transfer occurred directly between the host-frame and the incorporated oxidant (I_2) inside the channels (Supplementary Fig. 46a). Thus, after the electron transfer reaction, the incorporated I_2 molecules reduced to I_3^- (worked as counter anion) were kept included in the channels (Supplementary Fig. 46a). Conversely, in the present system, the ET oxidation of the inner guest was occurred outside the host-frame channels (Supplementary Fig. 46b), i.e., the crystal surface contacted with the oxidant ($[Fe(H_2O)_6](ClO_4)_3$ solid). It is worth noting that the size of $[Fe(H_2O)_6]^{3+}$ is too large to pass the window of the $(Zn^{2+})_4(L_A)_4(L_{A=O})_4$ host frame (0.90 nm × 0.92 nm) [Supplementary Fig. 44a], and the incorporation of the positively charged $[Fe(H_2O)_6]^{3+}$ into the positively charged $[(Zn)_4(L_A)_4(L_{A=O})_4]^{8+}$ host frame is unlikely to occur. Subsequent hole transfer and the incorporation of ClO_4^- into the nanotube channel resulted in oxidation of the whole range of the guest molecules inside the nanotube crystal (Supplementary Fig. 46b). Hence, the outer $[Fe(H_2O)_6]^{3+}$ reduced to $[Fe(H_2O)_6]^{2+}$ was kept outside the nanotube crystal (Supplementary Fig. 46b), where the additional counter anion of ClO_4^- was incorporated into the host-frame channels after the solid-state electron transfer. The size of ClO_4^- is small enough to pass the host-frame window size (0.90 nm

× 0.92 nm) [Supplementary Fig. 44b], where the incorporation of the negatively charged ClO_4^- into the positively charged $[(Zn)_4(L_{A=O})_8]^{8+}$ host frame is likely occur.

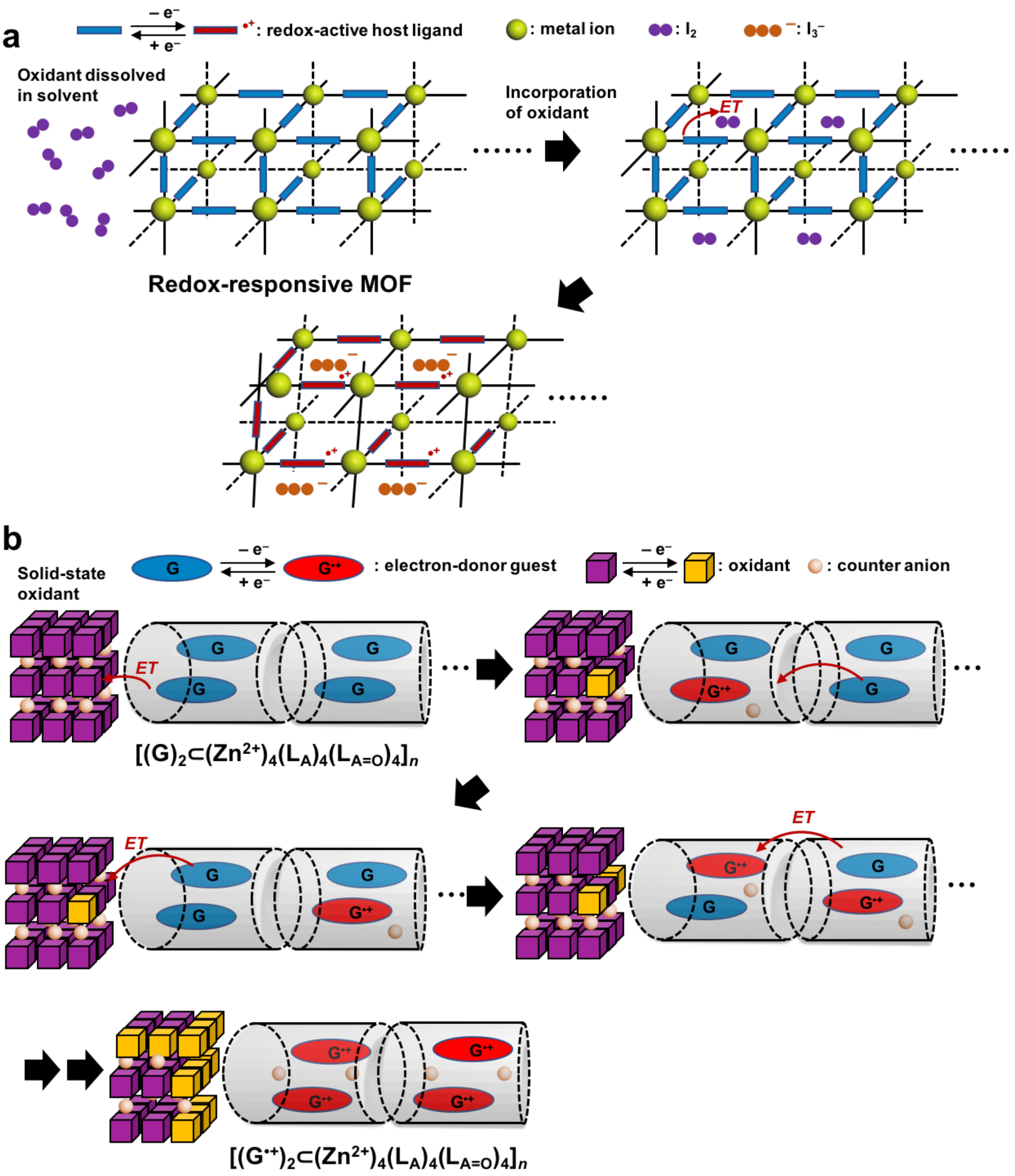

Supplementary Fig. 46 Schematic representation for (a) redox-stimuli responsive MOF and (b) solid-state oxidation of $[(D)_2\subset(Zn^{2+})_4(L_A)_4(L_{A=O})_4]_n$ crystals.

Supplementary Note 8. Determination of reorganization energy of ET in solids.

Since the above X-ray crystallography method can directly determine the initial and the final

structures of the ET in solids (Fig. 3e and f), this opens a way for direct determination of the reorganization energy (λ) of the thermal ET in solid. Besides, the ET oxidation of the nanotube crystal was completed by surface contact with the $[Fe(H_2O)_6](ClO_4)_3$ solid prior to the X-ray irradiation, and the X-ray irradiation was performed at 90 K, hence no further reaction could not procced during the X-ray irradiation. The determination of λ values was performed based on the Marcus-Hush two-state model.[13–15] To reduce the complexity, we exploited the structures of $TTF/(OTf^-)_2$ (initial structure) and $TTF^{\bullet+}/(OTf^-)_2/ClO_4^-$ from the X-ray crystal structures of $(TTF)_2{\subset}(Zn^{2+})_4(L_A)_4(L_{A=O})_4$ before and after the solid-state ET oxidation, respectively (Supplementary Fig. 47). Schematic representation for the Marcus-Hush calculation of reorganization energy was shown in Supplementary Fig. 48, wherein the single point energy of the isolated ClO_4^- was added to the energy of the initial structure ($TTF/(OTf^-)_2$) to match stoichiometry before and after the solid-state ET oxidation. The addition of the single point energy of the isolated ClO_4^- means that the ClO_4^- molecule is located away from the $TTF/(OTf^-)_2$ unit before the ET oxidation. In the same manner, the λ value for the ET oxidation of the inner Fc guest was determined. The present analysis revealed unusually large λ values for the solid-state ET oxidation of TTF and Fc inside the nanotube (1.36 and 2.23 eV, respectively). Such large λ values are mostly attributed to the incoming ClO_4^- molecules to compensate the additional positive charge on the electron donor molecules after the ET oxidation.

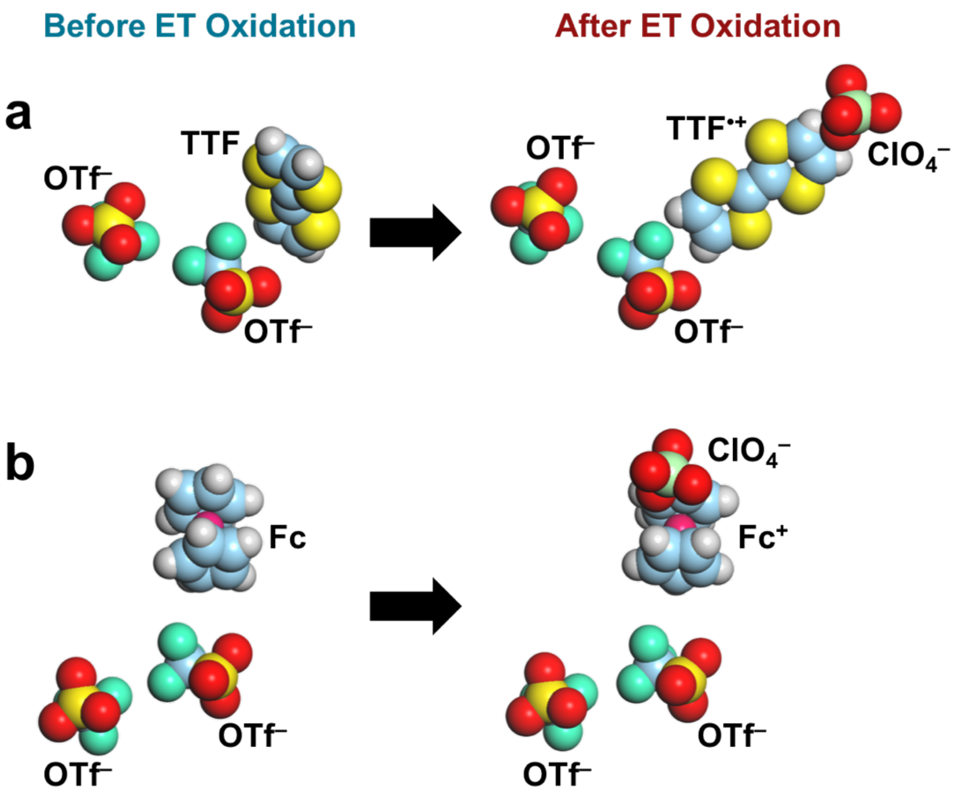

Supplementary Fig. 47 Core structures of **a**, $[(TTF)_2\subset(Zn^{2+})_4(L_A)_4(L_{A=O})_4]_n$, and **b**, $[(Fc)_2\subset(Zn^{2+})_4(L_A)_4(L_{A=O})_4]_n$ (left) before and (right) after ET oxidation with $[Fe(H_2O)_6](ClO_4)_3$ solids.

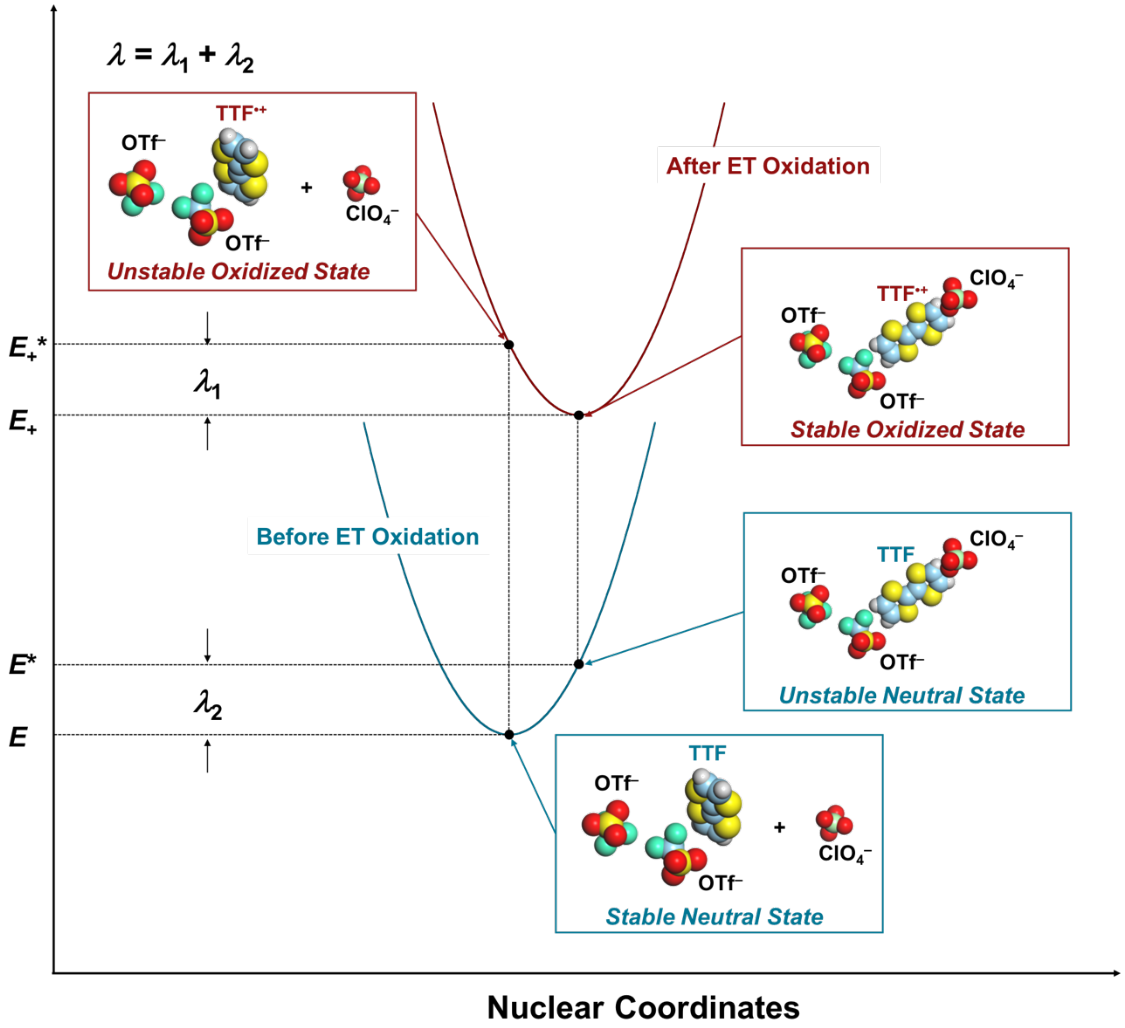

Supplementary Fig. 48 Schematic representation of reorganization energy (λ) for the solid-state ET oxidation of TTF in the $[(TTF)_2\subset(Zn^{2+})_4(L_A)_4(L_{A=O})_4]_n$ crystal. E_+^* and E_+ denote the single point energy (with DFT [CAM-UB3LYP/6-31G+(d,p)]) calculated the $(TTF^{\bullet+})(OTf^-)_2(ClO_4^-)$ units using the core structures found in the crystal structures of $[(TTF)_2\subset(Zn^{2+})_4(L_A)_4(L_{A=O})_4]_n$ before and after the solid-state ET oxidation, respectively. E and E^* denote the single point energy (with DFT [CAM-B3LYP/6-31G+(d,p)]) calculated the $(TTF)(OTf^-)_2$ units using the core structures found in the crystal structures of $[(TTF)_2\subset(Zn^{2+})_4(L_A)_4(L_{A=O})_4]_n$ before and after the solid-state ET oxidation, respectively, where the single point energy of the isolated ClO_4^- was added to the match stoichiometry before and after the solid-state ET oxidation. λ_1 and λ_2 are energy difference between E_+^* and E_+ ($\lambda_1 = E_+^* - E_+$) and that between E^* and E ($\lambda_2 = E^* - E$).

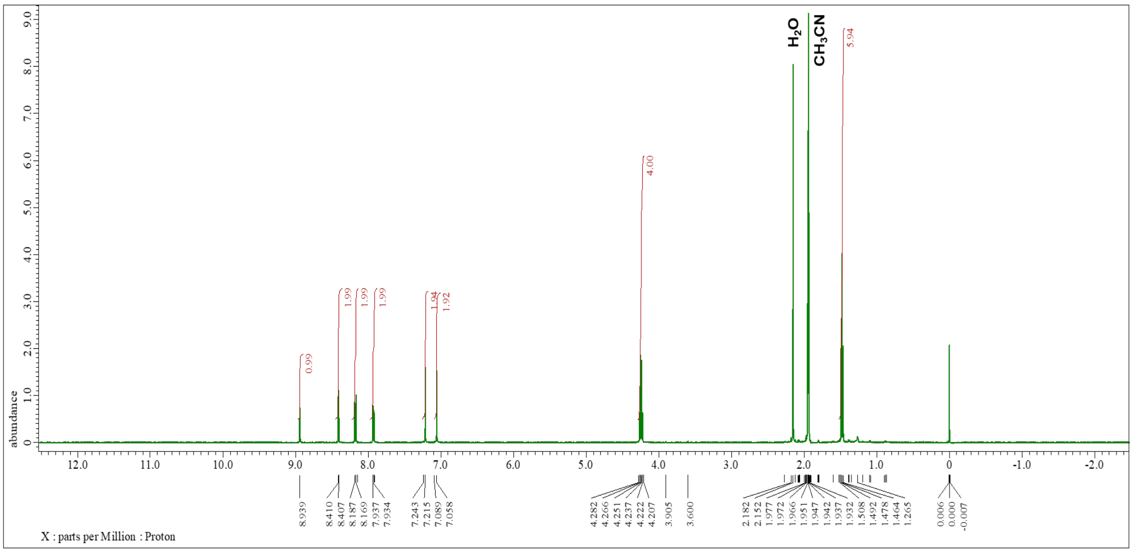

Supplementary Fig. 49 ^{1}H NMR spectrum of L_A in CD_3CN.

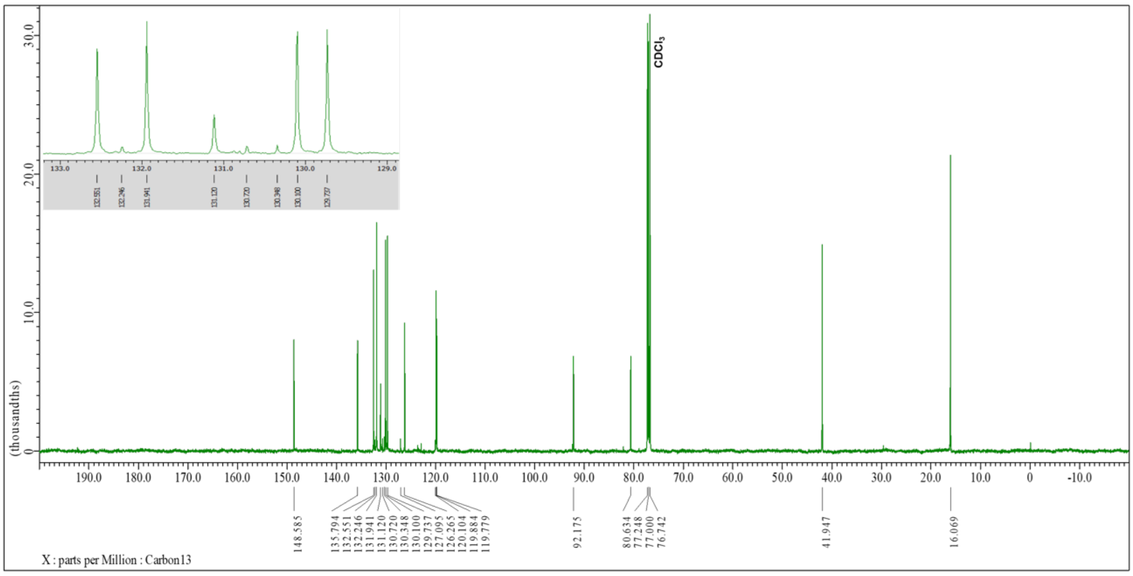

Supplementary Fig. 50. ^{13}C NMR spectrum of L_A in $CDCl_3$.

Supplementary Table 1

Crystallographic data and structure refinement details of $(Zn^{2+})_4(L_A)_4(L_{A=O})_4$

[1] Formula	$C_{219.88}H_{167.63}F_{12}N_{40}O_{16}S_4Zn_4$
[2] Formula weight	4243.81
[3] Temperature (K)	90(2)
[4] Wavelength (Å)	0.71073
[5] Crystal system	Monoclinic
[6] Space group	C2/c
[7] *a* (Å)	58.113(3)
b (Å)	13.9183(7)
c (Å)	38.9504(17)
α (°)	90
β (°)	117.066(2)
γ (°)	90
[8] Volume ($Å^3$)	28054(2)
[9] *Z*	4
[10] Density (calculated), (g/cm^3)	1.005
[11] F(000)	8748
[12] Crystal size (mm)	0.138×0.177×0.339
[13] μ (mm^{-1})	0.430
[14] Absorption correction type	Multi-Scan
[15] Total reflections	138251
[16] Unique reflections	22103
[17] Index ranges	−66≤ h ≤ 65, −15 ≤ k ≤ 15, −44 ≤ l ≤ 44
[18] Theta range for data collection (deg)	1.802–24.101
[19] R_{int}	0.0820
[20] Completeness (%)	99.3
[21] Data / restrains / parameters	22103/242/1357
[22] Goodness-of-fit on F^2	1.055
[23] R_1, wR_2 [I>2s(I)]	0.0923, 0.2760
[24] R_1, wR_2 (all data)	0.1477, 0.3054
[25] Largest diff. peak and hole (e. $Å^{-3}$)	0.663 and −0.604

Supplementary Table 2

Crystallographic data and structure refinement details of $(Fc)_2\subset(Zn^{2+})_4(L_A)_4(L_{A=O})_4$

[1] Formula	$C_{228.4}H_{176.4}F_{12}Fe_{0.84}N_{40}O_{16}S_4Zn_4$
[2] Formula weight	4401.92
[3] Temperature (K)	90(2)
[4] Wavelength (Å)	0.71073
[5] Crystal system	Monoclinic
[6] Space group	C2/c
[7] *a* (Å)	56.597(2)
b (Å)	13.6900(6)
c (Å)	38.8777(15)
α (°)	90
β (°)	117.327(2)
γ (°)	90
[8] Volume (Å^3)	26761(2)
[9] *Z*	4
[10] Density (calculated), (g/cm^3)	1.093
[11] F(000)	9075
[12] Crystal size (mm)	0.152×0.238×0.395
[13] μ (mm^{-1})	0.496
[14] Absorption correction type	Multi-Scan
[15] Total reflections	105479
[16] Unique reflections	15643
[17] Index ranges	$-58 \leq h \leq 58$, $-14 \leq k \leq 14$, $-40 \leq l \leq 40$
[18] Theta range for data collection (deg)	1.716–21.653
[19] R_{int}	0.0648
[20] Completeness (%)	99.7
[21] Data / restrains / parameters	15643/391/1442
[22] Goodness-of-fit on F^2	1.329
[23] R_1, wR_2 [I>2s(I)]	0.1309, 0.3591
[24] R_1, wR_2 (all data)	0.1758, 0.3881
[25] Largest diff. peak and hole (e. Å^{-3})	1.086 and −0.561

Supplementary Table 3

Crystallographic data and structure refinement details of $(TTF)_2\subset(Zn^{2+})_4(L_A)_4(L_{A=O})_4$

[1] Formula	$C_{227.56}H_{173.04}F_{12}N_{40}O_{16}S_{9.04}Zn_4$
[2] Formula weight	4502.97
[3] Temperature (K)	90(2)
[4] Wavelength (Å)	0.71073
[5] Crystal system	Monoclinic
[6] Space group	C2/c
[7] *a* (Å)	56.9041(18)
b (Å)	13.6996(4)
c (Å)	38.8225(12)
α (°)	90
β (°)	117.5340(10)
γ (°)	90
[8] Volume ($Å^3$)	26836.7(14)
[9] *Z*	4
[10] Density (calculated), (g/cm^3)	1.114
[11] F(000)	9276
[12] Crystal size (mm)	0.066×0.101×0.284
[13] μ (mm^{-1})	0.490
[14] Absorption correction type	Multi-Scan
[15] Total reflections	114723
[16] Unique reflections	15336
[17] Index ranges	$-58\leq h \leq 58, -14 \leq k \leq 14, -39 \leq l \leq 39$
[18] Theta range for data collection (deg)	1.825–21.480
[19] R_{int}	0.0679
[20] Completeness (%)	99.8
[21] Data / restrains / parameters	15336/311/1483
[22] Goodness-of-fit on F^2	1.239
[23] R_1, wR_2 [I>2s(I)]	0.1172, 0.3279
[24] R_1, wR_2 (all data)	0.1621, 0.3583
[25] Largest diff. peak and hole (e. $Å^{-3}$)	0.955 and –0.554

Supplementary Table 4

Crystallographic data and structure refinement details of $(Zn^{2+})_4(L_{A=O})_8$

[1] Formula	$C_{220}H_{168}F_{12}N_{40}O_{17.63}S_4Zn_4$
[2] Formula weight	4271.73
[3] Temperature (K)	90(2)
[4] Wavelength (Å)	0.71073
[5] Crystal system	Monoclinic
[6] Space group	C2/c
[7] *a* (Å)	57.446(4)
b (Å)	13.8083(10)
c (Å)	38.787(3)
α (°)	90
β (°)	118.041(3)
γ (°)	90
[8] Volume ($Å^3$)	27155(3)
[9] *Z*	4
[10] Density (calculated), (g/cm^3)	1.045
[11] F(000)	8804
[12] Crystal size (mm)	0.130×0.216×0.518
[13] μ (mm^{-1})	0.445
[14] Absorption correction type	Multi-Scan
[15] Total reflections	68478
[16] Unique reflections	8324
[17] Index ranges	$-48 \leq h \leq 47, -11 \leq k \leq 11, -32 \leq l \leq 32$
[18] Theta range for data collection (deg)	1.078–17.356
[19] R_{int}	0.1133
[20] Completeness (%)	98.7
[21] Data / restrains / parameters	8324/1241/1355
[22] Goodness-of-fit on F^2	0.999
[23] R_1, wR_2 [I>2s(I)]	0.1019, 0.2704
[24] R_1, wR_2 (all data)	0.1568, 0.3054
[25] Largest diff. peak and hole (e. $Å^{-3}$)	0.493 and −0.334

Supplementary Table 5

Crystallographic data and structure refinement details of oxidized $(TTF)_2 \subset (Zn^{2+})_4(L_A)_4(L_{A=O})_4$ with $[Fe(H_2O)_6](ClO_4)_3$

[1] Formula	$C_{227.56}H_{173.03}Cl_2F_{12}N_{40}O_{28}S_{9.03}Zn_4$
[2] Formula weight	4765.79
[3] Temperature (K)	90(2)
[4] Wavelength (Å)	0.71073
[5] Crystal system	Monoclinic
[6] Space group	C2/c
[7] *a* (Å)	56.592(3)
b (Å)	13.8354(8)
c (Å)	38.8581(18)
α (°)	90
β (°)	117.121(2)
γ (°)	90
[8] Volume ($Å^3$)	27080(2)
[9] *Z*	4
[10] Density (calculated), (g/cm^3)	1.169
[11] F(000)	9796
[12] Crystal size (mm)	0.133×0.259×0.478
[13] μ (mm^{-1})	0.512
[14] Absorption correction type	Multi-Scan
[15] Total reflections	82197
[16] Unique reflections	11403
[17] Index ranges	−52≤ h ≤ 52, −12 ≤ k ≤ 12, −36 ≤ l ≤ 36
[18] Theta range for data collection (deg)	1.568–19.351
[19] R_{int}	0.0867
[20] Completeness (%)	99.2
[21] Data / restrains / parameters	11403/1165/1497
[22] Goodness-of-fit on F^2	1.385
[23] R_1, wR_2 [I>2s(I)]	0.1418, 0.3707
[24] R_1, wR_2 (all data)	0.1908, 0.4004
[25] Largest diff. peak and hole (e. $Å^{-3}$)	0.721 and −0.389

Supplementary Table 6

Crystallographic data and structure refinement details of oxidized $(Fc)_2 \subset (Zn^{2+})_4(L_A)_4(L_{A=O})_4$ with $[Fe(H_2O)_6](ClO_4)_3$

[1] Formula	$C_{230.58}H_{178.58}Cl_{1.08}F_{12}Fe_{1.06}N_{40}O_{24.32}S_4Zn_4$
[2] Formula weight	4613.81
[3] Temperature (K)	90(2)
[4] Wavelength (Å)	0.71073
[5] Crystal system	Monoclinic
[6] Space group	C2/c
[7] *a* (Å)	55.367(5)
b (Å)	13.4749(12)
c (Å)	38.353(3)
α (°)	90
β (°)	116.843(3)
γ (°)	90
[8] Volume ($Å^3$)	25530(4)
[9] *Z*	4
[10] Density (calculated), (g/cm^3)	1.200
[11] F(000)	9498
[12] Crystal size (mm)	0.148×0.265×0.483
[13] μ (mm^{-1})	0.548
[14] Absorption correction type	Multi-Scan
[15] Total reflections	88101
[16] Unique reflections	12023
[17] Index ranges	$-52 \leq h \leq 53$, $-13 \leq k \leq 12$, $-36 \leq l \leq 36$
[18] Theta range for data collection (deg)	1.566–20.124
[19] R_{int}	0.0842
[20] Completeness (%)	99.2
[21] Data / restrains / parameters	12023/1238/1507
[22] Goodness-of-fit on F^2	1.501
[23] R_1, wR_2 [I>2s(I)]	0.1529, 0.3976
[24] R_1, wR_2 (all data)	0.1970, 0.4240
[25] Largest diff. peak and hole (e. $Å^{-3}$)	0.659 and –0.560

Supplementary references

1. S. A. Pourmousavi, P. Salehi, *Acta Chim. Slov.* **2009**, *56*, 734–739.
2. V. Martí-Centelles, P. D. Beer, *Chem. Eur. J.* **2015**, *21*, 9397–9404.
3. M. J. Frisch, G. W. Trucks, H. B. Schlegel, G. E. Scuseria, M. A. Robb, J. R. Cheeseman, G. Scalmani, V. Barone, B. Mennucci, G. A. Petersson, H. Nakatsuji, M. Caricato, X. Li, H. P. Hratchian, A. F. Izmaylov, J. Bloino, G. Zheng, J. L. Sonnenberg, M. Hada, M. Ehara, K. Toyota, R. Fukuda, J. Hasegawa, M. Ishida, T. Nakajima, Y. Honda, O. Kitao, H. Nakai, T. Vreven, J. A. Montgomery, J. E. Peralta Jr., F. Ogliaro, M. Bearpark, J. J. Heyd, E. Brothers, K. N. Kudin, V. N. Staroverov, T. Keith, R. Kobayashi, J. Normand, K. Raghavachari, A. Rendell, J. C. Burant, S. S. Iyengar, J. Tomasi, M. Cossi, N. Rega, J. M. Millam, M. Klene, J. E. Knox, J. B. Cross, V. Bakken, C. Adamo, J. Jaramillo, R. Gomperts, R. E. Stratmann, O. Yazyev, A. J. Austin, R. Cammi, C. Pomelli, J. W. Ochterski, R. L. Martin, K. Morokuma, V. G. Zakrzewski, G. A. Voth, P. Salvador, J. J. Dannenberg, S. Dapprich, A. D. Daniels, O. Farkas, J. B. Foresman, J. V. Ortiz, J. Cioslowski, D. J. Fox, Gaussian 09, revision D.01, (Gaussian Inc., 2013).
4. G. M. Sheldrick, *Acta. Cryst*. **2015**, *A71*, 3−8.
5. G. M. Sheldrick, *Acta. Cryst*. **2015**, *C71*, 3−8.
6. P. van. der. Sluis, A. L. Spek, *Acta Cryst.* **1990**, *A46*, 194−201.
7. A. L. Spek, *Acta. Cryst.* **2009**, *D65*, 148–155.
8. Yu, J., Yang, H., Jiang, Y. & Fu, H. *Chem. Eur. J.* **19**, 4271–4277 (2013).
9. Maglic, J.B. & Lavendomme, R. MoloVol : An easy-to-use program for analyzing cavities, volumes and surface areas of chemical structures. *J. Appl. Cryst*. **55**, 1033-1044 (2022).
10. Choi, H. J. & Suh, M. P. Dynamic and redox active pillared bilayer open framework: single-crystal-to-single-crystal transformations upon guest removal, guest exchange, and framework oxidation. *J. Am. Chem. Soc*. **126**, 15844–15851 (2004).

11. Su, J. et al. Redox activities of metal–organic frameworks incorporating rare-earth metal chains and tetrathiafulvalene linkers. *Inorg. Chem.* **58**, 3698–3706 (2019).

12. Su, J. et al. Redox-switchable breathing behavior in tetrathiafulvalene-based metal–organic frameworks. *Nat. Commun.* **8**, 2008 (2017).

13. Marcus, R. A. On the theory of oxidation-reduction reactions involving electron transfer. I. *J. Chem. Phys*. **24**, 966–978 (1956).

14. Hush, N. S. Adiabatic theory of outer sphere electron-transfer reactions in solution. *Trans. Faraday Soc*. **57**, 557−580 (1961).

15. Deng, W-Q & Goddard, W. A. Predictions of hole mobilities in oligoacene organic semiconductors from quantum mechanical calculations. *J. Phys. Chem. B*. **108**, 8614–8621 (2004).
