# Supplementary material for: Direct observation of electron transfer in solids through X-ray crystallography
Source: Nat Commun. 2024 May 23;15:4412. doi: 10.1038/s41467-024-48599-1 (PMC11116525; doi:10.1038/s41467-024-48599-1)
Supplement: Supplementary file 3 — Description of Additional Supplementary Files [file 41467_2024_48599_MOESM3_ESM.pdf]

Description of Additional Supplementary Files:

Supplementary Data 1: Atomic coordinates of ClO4 for DFT calculation in Suppl_Fig44.

Supplementary Data 2: Atomic coordinates of ClO4 for DFT calculation in Suppl_Fig48.

Supplementary Data 3: Atomic coordinates of the hydrogen-bonded complex for DFT calculation in Suppl_Fig41b.

Supplementary Data 4: Atomic coordinates of Fc for DFT calculation in Fig2b.

Supplementary Data 5: Atomic coordinates of Fc-nanotube core structure after ET oxidation.

Supplementary Data 6: Atomic coordinates of Fc-nanotube core structure before ET oxidation.

Supplementary Data 7: Atomic coordinates of [Fe(H2O)6]3+ for DFT calculation Suppl_Fig44.

Supplementary Data 8: Atomic coordinates of Fc+ for DFT calculation in Fig3d.

Supplementary Data 9: Atomic coordinates of TTF+ for DFT calculation in Fig3d.

Supplementary Data 10: Atomic coordinates of TTF for DFT calculation in Fig2b.

Supplementary Data 11: Atomic coordinates of TTF-nanotube core structure after ET oxidation for DFT calculation in Suppl_Fig48.

Supplementary Data 12: Atomic coordinates of TTF-nanotube core structure before ET oxidation for DFT calculation in Suppl_Fig48.

Supplementary Data 13: Cif file of [(Zn2+)4(LA)4(LA=O)4]n.

Supplementary Data 14: CheccCif file for [(Zn2+)4(LA)4(LA=O)4]n.

Supplementary Data 15: Cif file of [(Zn2+)4(LA)4(LA=O)4]n after ET oxidation.

Supplementary Data 16: Checkcif file for [(Zn2+)4(LA)4(LA=O)4]n after ET oxidation.

Supplementary Data 17: Cif file of [(Fc)2⊂(Zn2+)4(LA)4(LA=O)4]n.

Supplementary Data 18: Checkcif file of [(Fc)2⊂(Zn2+)4(LA)4(LA=O)4]n.

Supplementary Data 19: Cif file of [(Fc)2⊂(Zn2+)4(LA)4(LA=O)4]n after ET oxidation.

Supplementary Data 20: Checkcif file of [(Fc)2⊂(Zn2+)4(LA)4(LA=O)4]n after ET oxidation.

Supplementary Data 21: Cif file of [(TTF)2⊂(Zn2+)4(LA)4(LA=O)4]n.

Supplementary Data 22: Checkcif file of [(TTF)2⊂(Zn2+)4(LA)4(LA=O)4]n.

Supplementary Data 23: Cif file of [(TTF)2⊂(Zn2+)4(LA)4(LA=O)4]n after ET oxidation.

Supplementary Data 24: Checkcif file of [(TTF)2⊂(Zn2+)4(LA)4(LA=O)4]n after ET oxidation.

Supplementary Movie 1: Movie for the solid-sate ET oxidation of the [(TTF)2⊂(Zn2+)4(LA)4(LA=O)4]n crystal with [Fe(H2O)6]3+.

Supplementary Movie 2: Movie for the solid-sate ET oxidation of the [(Fc)2⊂(Zn2+)4(LA)4(LA=O)4]n crystal with [Fe(H2O)6]3+.

Supplementary Movie 3: Movie for the solid-sate ET oxidation of the [(Zn2+)4(LA)4(LA=O)4]n crystal with [Fe(H2O)6]3+.

Supplementary Movie 4: Schematic representation movie for the solid-sate ET oxidation of the [(TTF)2⊂(Zn2+)4(LA)4(LA=O)4]n.

Supplementary Movie 5: Schematic representation movie for the solid-sate ET oxidation of the [(Fc)2⊂(Zn2+)4(LA)4(LA=O)4]n.
