# Supplementary material for: Direct observation of electron transfer in solids through X-ray crystallography
Source: Nat Commun. 2024 May 23;15:4412. doi: 10.1038/s41467-024-48599-1 (PMC11116525; doi:10.1038/s41467-024-48599-1)

checkCIF/PLATON report

Structure factors have been supplied for datablock(s) 220627_Zn4L8Ksai_0m_a_sqd_sqd_sqd

THIS REPORT IS FOR GUIDANCE ONLY. IF USED AS PART OF A REVIEW PROCEDURE FOR PUBLICATION, IT SHOULD NOT REPLACE THE EXPERTISE OF AN EXPERIENCED CRYSTALLOGRAPHIC REFEREE.

No syntax errors found. CIF dictionary Interpreting this report

Datablock: 220627_Zn4L8Ksai_0m_a_sqd_sqd_sqd

```
Bond precision:   C-C = 0.0114 A          Wavelength=0.71073

Cell:             a=58.113(3)    b=13.9183(7)        c=38.9504(17)
                  alpha=90       beta=117.066(2)     gamma=90
Temperature:      90 K
```

	Calculated	Reported
Volume	28054(2)	28054(2)
Space group	C 2/c	C 2/c
Hall group	-C 2yc	-C 2yc
Moiety formula	C215.88 H167.64 N40 O4 Zn4, 4(C F3 O3 S) [+ solvent]	?
Sum formula	C219.88 H167.64 F12 N40 O16 S4 Zn4 [+ solvent]	C219.88 H167.63 F12 N40 O16 S4 Zn4
Mr	4243.95	4243.81
Dx,g cm-3	1.005	1.005
Z	4	4
Mu (mm-1)	0.430	0.430
F000	8747.7	8748.0
F000'	8757.73	
h,k,lmax	66,15,44	66,15,44
Nref	22262	22103
Tmin,Tmax	0.913,0.942	0.662,0.745
Tmin'	0.864	

```
Correction method= # Reported T Limits: Tmin=0.662 Tmax=0.745
AbsCorr = MULTI-SCAN

Data completeness= 0.993          Theta(max)= 24.101
```

R(reflections)= 0.0923(11537) wR2(reflections)= 0.3054(22103)

S = 1.055 Npar= 1357

The following ALERTS were generated. Each ALERT has the format
test-name_ALERT_alert-type_alert-level.
Click on the hyperlinks for more details of the test.

Alert level B

THETM01_ALERT_3_B The value of sine(theta_max)/wavelength is less than 0.575
Calculated sin(theta_max)/wavelength = 0.5745

Author Response: Despite long exposure times and rapid sample handling, few reflections at greater than 0.87 angstroms resolution were observed.

PLAT220_ALERT_2_B NonSolvent Resd 1 C Ueq(max)/Ueq(min) Range 6.5 Ratio

Author Response: This is due to the poor diffraction of the crystal.

PLAT242_ALERT_2_B Low 'MainMol' Ueq as Compared to Neighbors of C29 Check

Author Response: This alert is because ethyl groups of the ligands are partially disordered by thermal vibration or the relative positions of LA and LA=O in [(Zn2+)4(LA)4(LA=O)4]n are partially replaced.

PLAT242_ALERT_2_B Low 'MainMol' Ueq as Compared to Neighbors of C68 Check

Author Response: This alert is because ethyl groups of the ligands are partially disordered by thermal vibration or the relative positions of LA and LA=O in [(Zn2+)4(LA)4(LA=O)4]n are partially replaced.

PLAT242_ALERT_2_B Low 'MainMol' Ueq as Compared to Neighbors of C83 Check

Author Response: This alert is because ethyl groups of the ligands are partially disordered by thermal vibration or the relative positions of LA and LA=O in [(Zn2+)4(LA)4(LA=O)4]n are partially replaced.

PLAT990_ALERT_1_B Deprecated .res/.hkl Input Style SQUEEZE Job ... ! Note

---

Alert level C

ABSTY02_ALERT_1_C An _exptl_absorpt_correction_type has been given without
a literature citation. This should be contained in the
_exptl_absorpt_process_details field.
Absorption correction given as Multi-Scan

```
PLAT084_ALERT_3_C High wR2 Value (i.e. > 0.25) ...................       0.31 Report
PLAT222_ALERT_3_C NonSolvent Resd 1  H   Uiso(max)/Uiso(min) Range        7.7 Ratio
PLAT230_ALERT_2_C Hirshfeld Test Diff for    O1       --C68      .        6.0 s.u.
PLAT230_ALERT_2_C Hirshfeld Test Diff for    N5       --C26      .        5.6 s.u.
PLAT230_ALERT_2_C Hirshfeld Test Diff for    N19      --C107     .        5.7 s.u.
PLAT230_ALERT_2_C Hirshfeld Test Diff for    C24      --C25      .        5.4 s.u.
PLAT230_ALERT_2_C Hirshfeld Test Diff for    C26      --C27      .        6.3 s.u.
PLAT230_ALERT_2_C Hirshfeld Test Diff for    C51      --C52      .        5.7 s.u.
PLAT230_ALERT_2_C Hirshfeld Test Diff for    C69      --C70      .        6.4 s.u.
PLAT230_ALERT_2_C Hirshfeld Test Diff for    C80      --C81      .        5.7 s.u.
PLAT231_ALERT_4_C Hirshfeld Test (Solvent)   F1       --C1F      .        8.3 s.u.
PLAT234_ALERT_4_C Large Hirshfeld Difference Zn2A     --C24      .       0.17 Ang.
PLAT234_ALERT_4_C Large Hirshfeld Difference N2       --C2       .       0.18 Ang.
PLAT234_ALERT_4_C Large Hirshfeld Difference N2       --C3       .       0.17 Ang.
PLAT234_ALERT_4_C Large Hirshfeld Difference N3       --C11      .       0.20 Ang.
PLAT234_ALERT_4_C Large Hirshfeld Difference N8       --C43      .       0.16 Ang.
PLAT234_ALERT_4_C Large Hirshfeld Difference N10      --C53      .       0.16 Ang.
PLAT234_ALERT_4_C Large Hirshfeld Difference N14      --C80      .       0.17 Ang.
PLAT234_ALERT_4_C Large Hirshfeld Difference N15      --C78      .       0.16 Ang.
PLAT234_ALERT_4_C Large Hirshfeld Difference N17      --C86      .       0.16 Ang.
PLAT234_ALERT_4_C Large Hirshfeld Difference C8       --C13      .       0.18 Ang.
PLAT234_ALERT_4_C Large Hirshfeld Difference C12      --C13      .       0.17 Ang.
PLAT234_ALERT_4_C Large Hirshfeld Difference C17      --C18      .       0.19 Ang.
PLAT234_ALERT_4_C Large Hirshfeld Difference C38      --C39      .       0.19 Ang.
PLAT234_ALERT_4_C Large Hirshfeld Difference C42      --C43      .       0.17 Ang.
PLAT234_ALERT_4_C Large Hirshfeld Difference C44      --C45      .       0.18 Ang.
PLAT234_ALERT_4_C Large Hirshfeld Difference C89      --C90      .       0.17 Ang.
PLAT234_ALERT_4_C Large Hirshfeld Difference C100     --C102     .       0.16 Ang.
PLAT234_ALERT_4_C Large Hirshfeld Difference C102     --C103     .       0.17 Ang.
PLAT234_ALERT_4_C Large Hirshfeld Difference C105     --C106     .       0.16 Ang.
PLAT234_ALERT_4_C Large Hirshfeld Difference C107     --C108     .       0.18 Ang.
PLAT234_ALERT_4_C Large Hirshfeld Difference F5       --C2F      .       0.20 Ang.
PLAT241_ALERT_2_C High   'MainMol' Ueq as Compared to Neighbors of         N3 Check
PLAT241_ALERT_2_C High   'MainMol' Ueq as Compared to Neighbors of         N8 Check
PLAT241_ALERT_2_C High   'MainMol' Ueq as Compared to Neighbors of        N18 Check
PLAT241_ALERT_2_C High   'MainMol' Ueq as Compared to Neighbors of        C10 Check
PLAT241_ALERT_2_C High   'MainMol' Ueq as Compared to Neighbors of        C14 Check
PLAT241_ALERT_2_C High   'MainMol' Ueq as Compared to Neighbors of        C26 Check
PLAT241_ALERT_2_C High   'MainMol' Ueq as Compared to Neighbors of        C41 Check
PLAT241_ALERT_2_C High   'MainMol' Ueq as Compared to Neighbors of        C51 Check
PLAT241_ALERT_2_C High   'MainMol' Ueq as Compared to Neighbors of        C90 Check
PLAT241_ALERT_2_C High   'MainMol' Ueq as Compared to Neighbors of        C98 Check
PLAT242_ALERT_2_C Low    'MainMol' Ueq as Compared to Neighbors of        N17 Check
```

Author Response: This alert is because ethyl groups of the ligands are partially disordered by thermal vibration or the relative positions of LA and LA=O in [(Zn2+)4(LA)4(LA=O)4]n are partially replaced.

```
PLAT242_ALERT_2_C Low    'MainMol' Ueq as Compared to Neighbors of         C2 Check
```

Author Response: This alert is because ethyl groups of the ligands are partially disordered by thermal vibration or the relative positions of LA and LA=O in [(Zn2+)4(LA)4(LA=O)4]n are partially replaced.

```
PLAT242_ALERT_2_C Low    'MainMol' Ueq as Compared to Neighbors of        C39 Check
```

Author Response: This alert is because ethyl groups of the ligands are partially disordered by thermal vibration or the relative positions of LA and LA=O in [(Zn2+)4(LA)4(LA=O)4]n are partially replaced.

```
PLAT242_ALERT_2_C Low    'MainMol' Ueq as Compared to Neighbors of        C42 Check
```

Author Response: This alert is because ethyl groups of the ligands are partially disordered by thermal vibration or the relative positions of LA and LA=O in [(Zn2+)4(LA)4(LA=O)4]n are partially replaced.

```
PLAT242_ALERT_2_C Low    'MainMol' Ueq as Compared to Neighbors of        C56 Check
```

Author Response: This alert is because ethyl groups of the ligands are partially disordered by thermal vibration or the relative positions of LA and LA=O in [(Zn2+)4(LA)4(LA=O)4]n are partially replaced.

```
PLAT242_ALERT_2_C Low    'MainMol' Ueq as Compared to Neighbors of        C80 Check
```

Author Response: This alert is because ethyl groups of the ligands are partially disordered by thermal vibration or the relative positions of LA and LA=O in [(Zn2+)4(LA)4(LA=O)4]n are partially replaced.

```
PLAT244_ALERT_4_C Low    'Solvent' Ueq as Compared to Neighbors of         S1 Check
PLAT244_ALERT_4_C Low    'Solvent' Ueq as Compared to Neighbors of         S2 Check
PLAT260_ALERT_2_C Large Average Ueq of Residue Including      Zn1A      0.105 Check
PLAT260_ALERT_2_C Large Average Ueq of Residue Including        S1      0.104 Check
PLAT260_ALERT_2_C Large Average Ueq of Residue Including        S2      0.145 Check
PLAT341_ALERT_3_C Low Bond Precision on  C-C Bonds ...............     0.0114 Ang.
PLAT360_ALERT_2_C Short  C(sp3)-C(sp3) Bond  C26      - C27      .       1.36 Ang.
PLAT420_ALERT_2_C D-H Bond Without Acceptor  N13      --H13      .     Please Check
PLAT420_ALERT_2_C D-H Bond Without Acceptor  N18      --H18A     .     Please Check
PLAT767_ALERT_4_C INS Embedded LIST 6 Instruction Should be LIST 4     Please Check
PLAT906_ALERT_3_C Large K Value in the Analysis of Variance ......     24.974 Check
PLAT906_ALERT_3_C Large K Value in the Analysis of Variance ......      4.836 Check
PLAT906_ALERT_3_C Large K Value in the Analysis of Variance ......      2.351 Check
PLAT910_ALERT_3_C Missing # of FCF Reflection(s) Below Theta(Min).          9 Note
PLAT911_ALERT_3_C Missing FCF Refl Between Thmin & STh/L=    0.575        151 Report
PLAT913_ALERT_3_C Missing # of Very Strong Reflections in FCF ....         11 Note
PLAT918_ALERT_3_C Reflection(s) with I(obs) much Smaller I(calc) .          2 Check
```

Alert level G

```
FORMU01_ALERT_2_G  There is a discrepancy between the atom counts in the
            _chemical_formula_sum and the formula from the _atom_site* data.
            Atom count from _chemical_formula_sum:C219.88 H167.63 F12 N40 O16 S4 Z
            Atom count from the _atom_site data:  C219.88 H167.6400 F12 N40 O16 S4
PLAT002_ALERT_2_G Number of Distance or Angle Restraints on AtSite         23 Note
PLAT003_ALERT_2_G Number of Uiso or Uij Restrained non-H Atoms ...         34 Report
```

```
PLAT007_ALERT_5_G Number of Unrefined Donor-H Atoms .............          2 Report
PLAT041_ALERT_1_G Calc. and Reported SumFormula    Strings  Differ     Please Check
PLAT128_ALERT_4_G Alternate Setting for Input Space Group      C2/c       I2/a Note
PLAT171_ALERT_4_G The CIF-Embedded .res File Contains EADP Records          1 Report
PLAT172_ALERT_4_G The CIF-Embedded .res File Contains DFIX Records         11 Report
PLAT173_ALERT_4_G The CIF-Embedded .res File Contains DANG Records          5 Report
PLAT178_ALERT_4_G The CIF-Embedded .res File Contains SIMU Records          7 Report
PLAT186_ALERT_4_G The CIF-Embedded .res File Contains ISOR Records          1 Report
PLAT187_ALERT_4_G The CIF-Embedded .res File Contains RIGU Records          8 Report
PLAT190_ALERT_3_G A Non-default RIGU Restraint Value for First Par     0.0020 Report
PLAT190_ALERT_3_G A Non-default RIGU Restraint Value for First Par     0.0030 Report
PLAT230_ALERT_2_G Hirshfeld Test Diff for    C60      --C61      .       6.4 s.u.
PLAT244_ALERT_4_G Low    'Solvent' Ueq as Compared to Neighbors of        C1F Check
PLAT244_ALERT_4_G Low    'Solvent' Ueq as Compared to Neighbors of        C2F Check
PLAT301_ALERT_3_G Main Residue  Disorder .............(Resd  1  )          3% Note
PLAT304_ALERT_4_G Non-Integer Number of Atoms in ..... (Resd  1  )     431.52 Check
PLAT371_ALERT_2_G Long   C(sp2)-C(sp1) Bond  C7       - C8       .       1.43 Ang.
PLAT371_ALERT_2_G Long   C(sp2)-C(sp1) Bond  C22      - C23      .       1.44 Ang.
PLAT371_ALERT_2_G Long   C(sp2)-C(sp1) Bond  C34      - C35      .       1.43 Ang.
PLAT371_ALERT_2_G Long   C(sp2)-C(sp1) Bond  C46      - C48      .       1.44 Ang.
PLAT371_ALERT_2_G Long   C(sp2)-C(sp1) Bond  C59      - C60      .       1.43 Ang.
PLAT371_ALERT_2_G Long   C(sp2)-C(sp1) Bond  C61      - C62      .       1.46 Ang.
PLAT371_ALERT_2_G Long   C(sp2)-C(sp1) Bond  C73      - C75      .       1.45 Ang.
PLAT371_ALERT_2_G Long   C(sp2)-C(sp1) Bond  C76      - C77      .       1.43 Ang.
PLAT371_ALERT_2_G Long   C(sp2)-C(sp1) Bond  C86      - C87      .       1.46 Ang.
PLAT371_ALERT_2_G Long   C(sp2)-C(sp1) Bond  C88      - C89      .       1.45 Ang.
PLAT371_ALERT_2_G Long   C(sp2)-C(sp1) Bond  C100     - C102     .       1.45 Ang.
PLAT371_ALERT_2_G Long   C(sp2)-C(sp1) Bond  C103     - C104     .       1.42 Ang.
PLAT412_ALERT_2_G Short Intra XH3 .. XHn     H10A     ..H20C     .       1.43 Ang.
                                                      x,y,z  =      1_555 Check
PLAT412_ALERT_2_G Short Intra XH3 .. XHn     H10A     ..H20D     .       1.67 Ang.
                                                      x,y,z  =      1_555 Check
PLAT412_ALERT_2_G Short Intra XH3 .. XHn     H10A     ..H20E     .       1.09 Ang.
                                                      x,y,z  =      1_555 Check
PLAT412_ALERT_2_G Short Intra XH3 .. XHn     H28D     ..H29A     .       1.56 Ang.
                                                      x,y,z  =      1_555 Check
PLAT412_ALERT_2_G Short Intra XH3 .. XHn     H28D     ..H29B     .       1.88 Ang.
                                                      x,y,z  =      1_555 Check
PLAT412_ALERT_2_G Short Intra XH3 .. XHn     H28E     ..H29A     .       1.99 Ang.
                                                      x,y,z  =      1_555 Check
PLAT412_ALERT_2_G Short Intra XH3 .. XHn     H28E     ..H29B     .       1.68 Ang.
                                                      x,y,z  =      1_555 Check
PLAT412_ALERT_2_G Short Intra XH3 .. XHn     H28F     ..H29A     .       1.34 Ang.
                                                      x,y,z  =      1_555 Check
PLAT413_ALERT_2_G Short Inter XH3 .. XHn     H44      ..H28B     .       1.90 Ang.
                                                1-x,1-y,1-z  =      5_666 Check
PLAT432_ALERT_2_G Short Inter X...Y Contact  O5A      ..C208     .       2.93 Ang.
                                               x,2-y,-1/2+z  =      6_575 Check
PLAT432_ALERT_2_G Short Inter X...Y Contact  O6A      ..C28      .       2.83 Ang.
                                                1-x,1-y,1-z  =      5_666 Check
PLAT606_ALERT_4_G Solvent Accessible VOID(S) in Structure .......           ! Info
PLAT794_ALERT_5_G Tentative Bond Valency for Zn1A      (II)      .       1.89 Info
PLAT802_ALERT_4_G CIF Input Record(s) with more than 80 Characters          2 Info
PLAT860_ALERT_3_G Number of Least-Squares Restraints ............         242 Note
PLAT869_ALERT_4_G ALERTS Related to the Use of SQUEEZE  Suppressed          ! Info
PLAT883_ALERT_1_G No Info/Value for _atom_sites_solution_primary .     Please Do !
PLAT933_ALERT_2_G Number of HKL-OMIT Records in Embedded .res File          2 Note
```

```
PLAT961_ALERT_5_G Dataset Contains no Negative Intensities .......     Please Check
PLAT978_ALERT_2_G Number C-C Bonds with Positive Residual Density.          0 Info
```

```
   0 ALERT level A = Most likely a serious problem - resolve or explain
   6 ALERT level B = A potentially serious problem, consider carefully
  66 ALERT level C = Check. Ensure it is not caused by an omission or oversight
  51 ALERT level G = General information/check it is not something unexpected

   4 ALERT type 1 CIF construction/syntax error, inconsistent or missing data
  63 ALERT type 2 Indicator that the structure model may be wrong or deficient
  15 ALERT type 3 Indicator that the structure quality may be low
  38 ALERT type 4 Improvement, methodology, query or suggestion
   3 ALERT type 5 Informative message, check
```

It is advisable to attempt to resolve as many as possible of the alerts in all categories. Often the minor alerts point to easily fixed oversights, errors and omissions in your CIF or refinement strategy, so attention to these fine details can be worthwhile. In order to resolve some of the more serious problems it may be necessary to carry out additional measurements or structure refinements. However, the purpose of your study may justify the reported deviations and the more serious of these should normally be commented upon in the discussion or experimental section of a paper or in the "special_details" fields of the CIF. checkCIF was carefully designed to identify outliers and unusual parameters, but every test has its limitations and alerts that are not important in a particular case may appear. Conversely, the absence of alerts does not guarantee there are no aspects of the results needing attention. It is up to the individual to critically assess their own results and, if necessary, seek expert advice.

Publication of your CIF in IUCr journals

A basic structural check has been run on your CIF. These basic checks will be run on all CIFs submitted for publication in IUCr journals (*Acta Crystallographica*, *Journal of Applied Crystallography*, *Journal of Synchrotron Radiation*); however, if you intend to submit to *Acta Crystallographica Section C* or *E* or *IUCrData*, you should make sure that full publication checks are run on the final version of your CIF prior to submission.

Publication of your CIF in other journals

Please refer to the *Notes for Authors* of the relevant journal for any special instructions relating to CIF submission.

PLATON version of 28/11/2022; check.def file version of 28/11/2022

Datablock 220627_Zn4L8Ksai_0m_a_sqd_sqd_sqd - ellipsoid plot

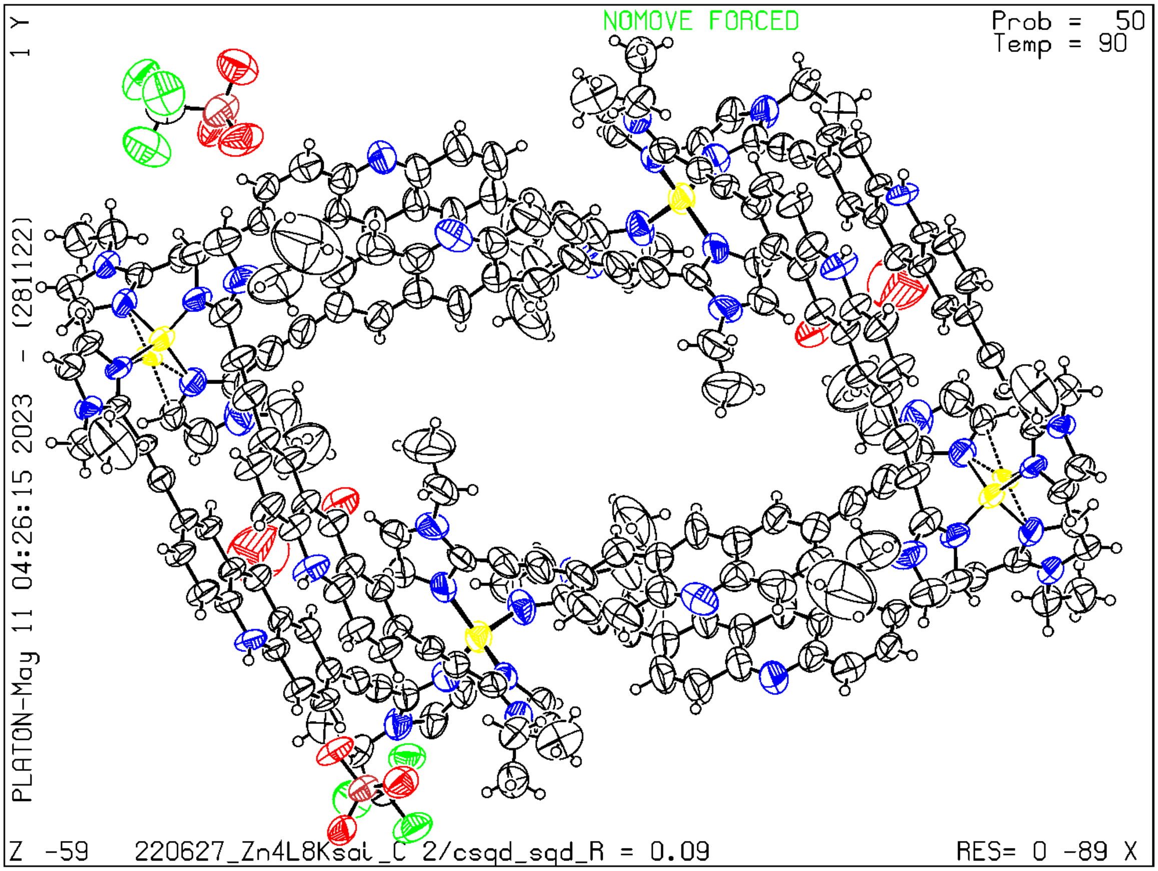

Supplement: Supplementary file 17 — Supplementary data file 14 [file 41467_2024_48599_MOESM17_ESM.pdf]
