# Supplementary material for: Direct observation of electron transfer in solids through X-ray crystallography
Source: Nat Commun. 2024 May 23;15:4412. doi: 10.1038/s41467-024-48599-1 (PMC11116525; doi:10.1038/s41467-024-48599-1)

checkCIF/PLATON report

Structure factors have been supplied for datablock(s) 230718_Zn4L8apo_FeClO4_0m_a_sqd

THIS REPORT IS FOR GUIDANCE ONLY. IF USED AS PART OF A REVIEW PROCEDURE FOR PUBLICATION, IT SHOULD NOT REPLACE THE EXPERTISE OF AN EXPERIENCED CRYSTALLOGRAPHIC REFEREE.

No syntax errors found.   CIF dictionary   Interpreting this report

Datablock: 230718_Zn4L8apo_FeClO4_0m_a_sqd

Bond precision:	C-C = 0.0285 A	Wavelength=0.71073	
Cell:	a=57.446(4)	b=13.8083(10)	c=38.787(3)
	alpha=90	beta=118.041(3)	gamma=90
Temperature:	90 K		

	Calculated	Reported
Volume	27155(4)	27155(3)
Space group	C 2/c	C 2/c
Hall group	-C 2yc	-C 2yc
Moiety formula	C216 H168 N40 O5.62 Zn4, 4(C F3 O3 S) [+ solvent]	?
Sum formula	C220 H168 F12 N40 O17.62 S4 Zn4 [+ solvent]	C220 H168 F12 N40 O17.63 S4 Zn4
Mr	4271.68	4271.73
Dx,g cm-3	1.045	1.045
Z	4	4
Mu (mm-1)	0.445	0.445
F000	8803.8	8804.0
F000'	8813.96	
h,k,lmax	48,11,32	48,11,32
Nref	8429	8324
Tmin,Tmax	0.891,0.944	0.582,0.744
Tmin'	0.794	

Correction method= # Reported T Limits: Tmin=0.582 Tmax=0.744
AbsCorr = MULTI-SCAN

Data completeness= 0.988   Theta(max)= 17.356

R(reflections)= 0.1019(4197) wR2(reflections)= 0.3054(8324)

S = 0.999 Npar= 1355

The following ALERTS were generated. Each ALERT has the format
test-name_ALERT_alert-type_alert-level.
Click on the hyperlinks for more details of the test.

Alert level A

THETM01_ALERT_3_A The value of sine(theta_max)/wavelength is less than 0.550
Calculated sin(theta_max)/wavelength = 0.4197

Author Response: Despite long exposure times and rapid sample handling, few reflections at greater than 1.20 angstroms resolution were observed.

Alert level B

PLAT088_ALERT_3_B Poor Data / Parameter Ratio .... 6.14 Note

Author Response: The low data to parameter ratio results from the poor diffraction of the crystal.

PLAT242_ALERT_2_B Low 'MainMol' Ueq as Compared to Neighbors of C26 Check

Author Response: This alert is because ethyl groups of the ligands are partially disordered by thermal vibration.

PLAT242_ALERT_2_B Low 'MainMol' Ueq as Compared to Neighbors of C80 Check

Author Response: This alert is because ethyl groups of the ligands are partially disordered by thermal vibration.

PLAT242_ALERT_2_B Low 'MainMol' Ueq as Compared to Neighbors of C107 Check

Author Response: This alert is because ethyl groups of the ligands are partially disordered by thermal vibration.

PLAT341_ALERT_3_B Low Bond Precision on C-C Bonds .... 0.02854 Ang.

Author Response: The low bond precision arises from the limited resolution of the data.

```
PLAT990_ALERT_1_B Deprecated .res/.hkl Input Style SQUEEZE Job ...          ! Note
```

Alert level C

```
ABSTY02_ALERT_1_C  An _exptl_absorpt_correction_type has been given without
            a literature citation. This should be contained in the
            _exptl_absorpt_process_details field.
            Absorption correction given as Multi-Scan
PLAT084_ALERT_3_C High wR2 Value (i.e. > 0.25) ...................       0.31 Report
PLAT220_ALERT_2_C NonSolvent   Resd 1  C   Ueq(max)/Ueq(min) Range        5.7 Ratio
PLAT222_ALERT_3_C NonSolvent Resd 1  H   Uiso(max)/Uiso(min) Range        6.8 Ratio
PLAT230_ALERT_2_C Hirshfeld Test Diff for    C82      --C83      .        5.3 s.u.
PLAT230_ALERT_2_C Hirshfeld Test Diff for    C107     --C108     .        5.9 s.u.
PLAT231_ALERT_4_C Hirshfeld Test (Solvent)   S2       --O10      .        6.5 s.u.
PLAT234_ALERT_4_C Large Hirshfeld Difference Zn01     --N16_a    .       0.20 Ang.
PLAT234_ALERT_4_C Large Hirshfeld Difference Zn02     --N19      .       0.18 Ang.
PLAT234_ALERT_4_C Large Hirshfeld Difference O4       --C95      .       0.25 Ang.
PLAT234_ALERT_4_C Large Hirshfeld Difference N5       --C23      .       0.22 Ang.
PLAT234_ALERT_4_C Large Hirshfeld Difference N9       --C50      .       0.22 Ang.
PLAT234_ALERT_4_C Large Hirshfeld Difference N13      --C65      .       0.21 Ang.
PLAT234_ALERT_4_C Large Hirshfeld Difference N15      --C80      .       0.21 Ang.
PLAT234_ALERT_4_C Large Hirshfeld Difference N16      --C86      .       0.21 Ang.
PLAT234_ALERT_4_C Large Hirshfeld Difference N17      --C86      .       0.21 Ang.
PLAT234_ALERT_4_C Large Hirshfeld Difference N18      --C92      .       0.21 Ang.
PLAT234_ALERT_4_C Large Hirshfeld Difference C19      --C20      .       0.22 Ang.
PLAT234_ALERT_4_C Large Hirshfeld Difference C42      --C47      .       0.21 Ang.
PLAT234_ALERT_4_C Large Hirshfeld Difference C46      --C48      .       0.22 Ang.
PLAT234_ALERT_4_C Large Hirshfeld Difference C64      --C65      .       0.19 Ang.
PLAT234_ALERT_4_C Large Hirshfeld Difference C66      --C67      .       0.19 Ang.
PLAT234_ALERT_4_C Large Hirshfeld Difference C66      --C68      .       0.19 Ang.
PLAT234_ALERT_4_C Large Hirshfeld Difference C71      --C72      .       0.21 Ang.
PLAT234_ALERT_4_C Large Hirshfeld Difference C88      --C89      .       0.22 Ang.
PLAT234_ALERT_4_C Large Hirshfeld Difference C90      --C91      .       0.22 Ang.
PLAT234_ALERT_4_C Large Hirshfeld Difference C100     --C102     .       0.22 Ang.
PLAT234_ALERT_4_C Large Hirshfeld Difference S1       --O7       .       0.19 Ang.
PLAT234_ALERT_4_C Large Hirshfeld Difference S2       --O9       .       0.24 Ang.
PLAT241_ALERT_2_C High   'MainMol' Ueq as Compared to Neighbors of         N3 Check
PLAT241_ALERT_2_C High   'MainMol' Ueq as Compared to Neighbors of         N8 Check
PLAT241_ALERT_2_C High   'MainMol' Ueq as Compared to Neighbors of        N18 Check
PLAT241_ALERT_2_C High   'MainMol' Ueq as Compared to Neighbors of         C3 Check
PLAT241_ALERT_2_C High   'MainMol' Ueq as Compared to Neighbors of        C30 Check
PLAT241_ALERT_2_C High   'MainMol' Ueq as Compared to Neighbors of        C36 Check
PLAT241_ALERT_2_C High   'MainMol' Ueq as Compared to Neighbors of        C48 Check
PLAT241_ALERT_2_C High   'MainMol' Ueq as Compared to Neighbors of        C53 Check
PLAT241_ALERT_2_C High   'MainMol' Ueq as Compared to Neighbors of        C75 Check
PLAT241_ALERT_2_C High   'MainMol' Ueq as Compared to Neighbors of       C104 Check
PLAT241_ALERT_2_C High   'MainMol' Ueq as Compared to Neighbors of       C106 Check
PLAT242_ALERT_2_C Low    'MainMol' Ueq as Compared to Neighbors of         N2 Check
```

Author Response: This alert is because ethyl groups of the ligands are partially disordered by thermal vibration.

PLAT242_ALERT_2_C Low 'MainMol' Ueq as Compared to Neighbors of N7 Check

Author Response: This alert is because ethyl groups of the ligands are partially disordered by thermal vibration.

PLAT242_ALERT_2_C Low 'MainMol' Ueq as Compared to Neighbors of N10 Check

Author Response: This alert is because ethyl groups of the ligands are partially disordered by thermal vibration.

PLAT242_ALERT_2_C Low 'MainMol' Ueq as Compared to Neighbors of N15 Check

Author Response: This alert is because ethyl groups of the ligands are partially disordered by thermal vibration.

PLAT242_ALERT_2_C Low 'MainMol' Ueq as Compared to Neighbors of N20 Check

Author Response: This alert is because ethyl groups of the ligands are partially disordered by thermal vibration.

PLAT242_ALERT_2_C Low 'MainMol' Ueq as Compared to Neighbors of C2 Check

Author Response: This alert is because ethyl groups of the ligands are partially disordered by thermal vibration.

PLAT242_ALERT_2_C Low 'MainMol' Ueq as Compared to Neighbors of C14 Check

Author Response: This alert is because ethyl groups of the ligands are partially disordered by thermal vibration.

PLAT242_ALERT_2_C Low 'MainMol' Ueq as Compared to Neighbors of C16 Check

Author Response: This alert is because ethyl groups of the ligands are partially disordered by thermal vibration.

PLAT242_ALERT_2_C Low 'MainMol' Ueq as Compared to Neighbors of C38 Check

Author Response: This alert is because ethyl groups of the ligands are partially disordered by thermal vibration.

PLAT242_ALERT_2_C Low 'MainMol' Ueq as Compared to Neighbors of C41 Check

Author Response: This alert is because ethyl groups of the ligands are partially disordered by thermal vibration.

```
PLAT242_ALERT_2_C Low    'MainMol' Ueq as Compared to Neighbors of        C43 Check
```

Author Response: This alert is because ethyl groups of the ligands are partially disordered by thermal vibration.

```
PLAT242_ALERT_2_C Low    'MainMol' Ueq as Compared to Neighbors of        C46 Check
```

Author Response: This alert is because ethyl groups of the ligands are partially disordered by thermal vibration.

```
PLAT242_ALERT_2_C Low    'MainMol' Ueq as Compared to Neighbors of        C56 Check
```

Author Response: This alert is because ethyl groups of the ligands are partially disordered by thermal vibration.

```
PLAT242_ALERT_2_C Low    'MainMol' Ueq as Compared to Neighbors of        C68 Check
```

Author Response: This alert is because ethyl groups of the ligands are partially disordered by thermal vibration.

```
PLAT242_ALERT_2_C Low    'MainMol' Ueq as Compared to Neighbors of        C73 Check
```

Author Response: This alert is because ethyl groups of the ligands are partially disordered by thermal vibration.

```
PLAT242_ALERT_2_C Low    'MainMol' Ueq as Compared to Neighbors of        C83 Check
```

Author Response: This alert is because ethyl groups of the ligands are partially disordered by thermal vibration.

```
PLAT242_ALERT_2_C Low    'MainMol' Ueq as Compared to Neighbors of        C86 Check
```

Author Response: This alert is because ethyl groups of the ligands are partially disordered by thermal vibration.

```
PLAT242_ALERT_2_C Low    'MainMol' Ueq as Compared to Neighbors of        C95 Check
```

Author Response: This alert is because ethyl groups of the ligands are partially disordered by thermal vibration.

```
PLAT244_ALERT_4_C Low    'Solvent' Ueq as Compared to Neighbors of         S1 Check
PLAT244_ALERT_4_C Low    'Solvent' Ueq as Compared to Neighbors of         S2 Check
PLAT250_ALERT_2_C Large U3/U1 Ratio for Average U(i,j) Tensor ....        2.2 Note
PLAT260_ALERT_2_C Large Average Ueq of Residue Including      Zn01      0.169 Check
PLAT260_ALERT_2_C Large Average Ueq of Residue Including        S1      0.238 Check
PLAT260_ALERT_2_C Large Average Ueq of Residue Including        S2      0.202 Check
PLAT360_ALERT_2_C Short  C(sp3)-C(sp3) Bond  C53      - C54      .       1.39 Ang.
PLAT372_ALERT_2_C Short  C(sp)-C(sp)   Bond  C33      - C34      .       1.14 Ang.
PLAT420_ALERT_2_C D-H Bond Without Acceptor  N3       --H3Z      .     Please Check
PLAT420_ALERT_2_C D-H Bond Without Acceptor  N8       --H8Z      .     Please Check
PLAT420_ALERT_2_C D-H Bond Without Acceptor  N13      --H13Z     .     Please Check
PLAT420_ALERT_2_C D-H Bond Without Acceptor  N18      --H18Z     .     Please Check
PLAT767_ALERT_4_C INS Embedded LIST 6 Instruction Should be LIST 4     Please Check
PLAT906_ALERT_3_C Large K Value in the Analysis of Variance ......     25.423 Check
PLAT906_ALERT_3_C Large K Value in the Analysis of Variance ......      5.346 Check
PLAT906_ALERT_3_C Large K Value in the Analysis of Variance ......      2.414 Check
PLAT911_ALERT_3_C Missing FCF Refl Between Thmin & STh/L=    0.420        105 Report
PLAT918_ALERT_3_C Reflection(s) with I(obs) much Smaller I(calc) .          7 Check
PLAT922_ALERT_1_C wR2 in the CIF and FCF Differ by ..............      0.0025 Check
```

Alert level G

```
FORMU01_ALERT_2_G  There is a discrepancy between the atom counts in the
            _chemical_formula_sum and the formula from the _atom_site* data.
            Atom count from _chemical_formula_sum:C220 H168 F12 N40 O17.63 S4 Zn4
            Atom count from the _atom_site data:  C220 H168 F12 N40 O17.62 S4 Zn4
PLAT002_ALERT_2_G Number of Distance or Angle Restraints on AtSite        138 Note
PLAT003_ALERT_2_G Number of Uiso or Uij Restrained non-H Atoms ...        126 Report
PLAT007_ALERT_5_G Number of Unrefined Donor-H Atoms .............           4 Report
PLAT041_ALERT_1_G Calc. and Reported SumFormula    Strings  Differ     Please Check
PLAT128_ALERT_4_G Alternate Setting for Input Space Group     C2/c       I2/a Note
PLAT172_ALERT_4_G The CIF-Embedded .res File Contains DFIX Records         56 Report
PLAT173_ALERT_4_G The CIF-Embedded .res File Contains DANG Records         52 Report
PLAT174_ALERT_4_G The CIF-Embedded .res File Contains FLAT Records          2 Report
PLAT178_ALERT_4_G The CIF-Embedded .res File Contains SIMU Records         12 Report
PLAT186_ALERT_4_G The CIF-Embedded .res File Contains ISOR Records          2 Report
PLAT187_ALERT_4_G The CIF-Embedded .res File Contains RIGU Records          6 Report
PLAT244_ALERT_4_G Low    'Solvent' Ueq as Compared to Neighbors of        C1A Check
PLAT244_ALERT_4_G Low    'Solvent' Ueq as Compared to Neighbors of        C2A Check
PLAT301_ALERT_3_G Main Residue  Disorder .............(Resd  1  )          1% Note
PLAT304_ALERT_4_G Non-Integer Number of Atoms in ..... (Resd  1  )     433.62 Check
PLAT371_ALERT_2_G Long   C(sp2)-C(sp1) Bond  C7       - C8       .       1.42 Ang.
PLAT371_ALERT_2_G Long   C(sp2)-C(sp1) Bond  C19      - C21      .       1.43 Ang.
PLAT371_ALERT_2_G Long   C(sp2)-C(sp1) Bond  C22      - C23      .       1.42 Ang.
PLAT371_ALERT_2_G Long   C(sp2)-C(sp1) Bond  C34      - C35      .       1.50 Ang.
PLAT371_ALERT_2_G Long   C(sp2)-C(sp1) Bond  C46      - C48      .       1.43 Ang.
PLAT371_ALERT_2_G Long   C(sp2)-C(sp1) Bond  C61      - C62      .       1.47 Ang.
PLAT371_ALERT_2_G Long   C(sp2)-C(sp1) Bond  C73      - C75      .       1.50 Ang.
PLAT371_ALERT_2_G Long   C(sp2)-C(sp1) Bond  C86      - C87      .       1.46 Ang.
PLAT371_ALERT_2_G Long   C(sp2)-C(sp1) Bond  C100     - C102     .       1.41 Ang.
PLAT606_ALERT_4_G Solvent Accessible VOID(S) in Structure ........          ! Info
PLAT720_ALERT_4_G Number of Unusual/Non-Standard Labels ..........          2 Note
PLAT794_ALERT_5_G Tentative Bond Valency for Zn01      (II)      .       2.00 Info
PLAT794_ALERT_5_G Tentative Bond Valency for Zn02      (II)      .       1.98 Info
PLAT802_ALERT_4_G CIF Input Record(s) with more than 80 Characters          2 Info
PLAT860_ALERT_3_G Number of Least-Squares Restraints ............        1241 Note
PLAT869_ALERT_4_G ALERTS Related to the Use of SQUEEZE  Suppressed          ! Info
```

```
PLAT883_ALERT_1_G No Info/Value for _atom_sites_solution_primary .     Please Do !
PLAT910_ALERT_3_G Missing # of FCF Reflection(s) Below Theta(Min).          1 Note
PLAT933_ALERT_2_G Number of HKL-OMIT Records in Embedded .res File          2 Note
PLAT961_ALERT_5_G Dataset Contains no Negative Intensities .......     Please Check
PLAT978_ALERT_2_G Number C-C Bonds with Positive Residual Density.          0 Info
```

```
  1 ALERT level A = Most likely a serious problem - resolve or explain
  6 ALERT level B = A potentially serious problem, consider carefully
 77 ALERT level C = Check. Ensure it is not caused by an omission or oversight
 37 ALERT level G = General information/check it is not something unexpected

  5 ALERT type 1 CIF construction/syntax error, inconsistent or missing data
 59 ALERT type 2 Indicator that the structure model may be wrong or deficient
 13 ALERT type 3 Indicator that the structure quality may be low
 40 ALERT type 4 Improvement, methodology, query or suggestion
  4 ALERT type 5 Informative message, check
```

It is advisable to attempt to resolve as many as possible of the alerts in all categories. Often the minor alerts point to easily fixed oversights, errors and omissions in your CIF or refinement strategy, so attention to these fine details can be worthwhile. In order to resolve some of the more serious problems it may be necessary to carry out additional measurements or structure refinements. However, the purpose of your study may justify the reported deviations and the more serious of these should normally be commented upon in the discussion or experimental section of a paper or in the "special_details" fields of the CIF. checkCIF was carefully designed to identify outliers and unusual parameters, but every test has its limitations and alerts that are not important in a particular case may appear. Conversely, the absence of alerts does not guarantee there are no aspects of the results needing attention. It is up to the individual to critically assess their own results and, if necessary, seek expert advice.

Publication of your CIF in IUCr journals

A basic structural check has been run on your CIF. These basic checks will be run on all CIFs submitted for publication in IUCr journals (*Acta Crystallographica*, *Journal of Applied Crystallography*, *Journal of Synchrotron Radiation*); however, if you intend to submit to *Acta Crystallographica Section C* or *E* or *IUCrData*, you should make sure that full publication checks are run on the final version of your CIF prior to submission.

Publication of your CIF in other journals

Please refer to the *Notes for Authors* of the relevant journal for any special instructions relating to CIF submission.

PLATON version of 06/07/2023; check.def file version of 30/06/2023

Datablock 230718_Zn4L8apo_FeClO4_0m_a_sqd - ellipsoid plot

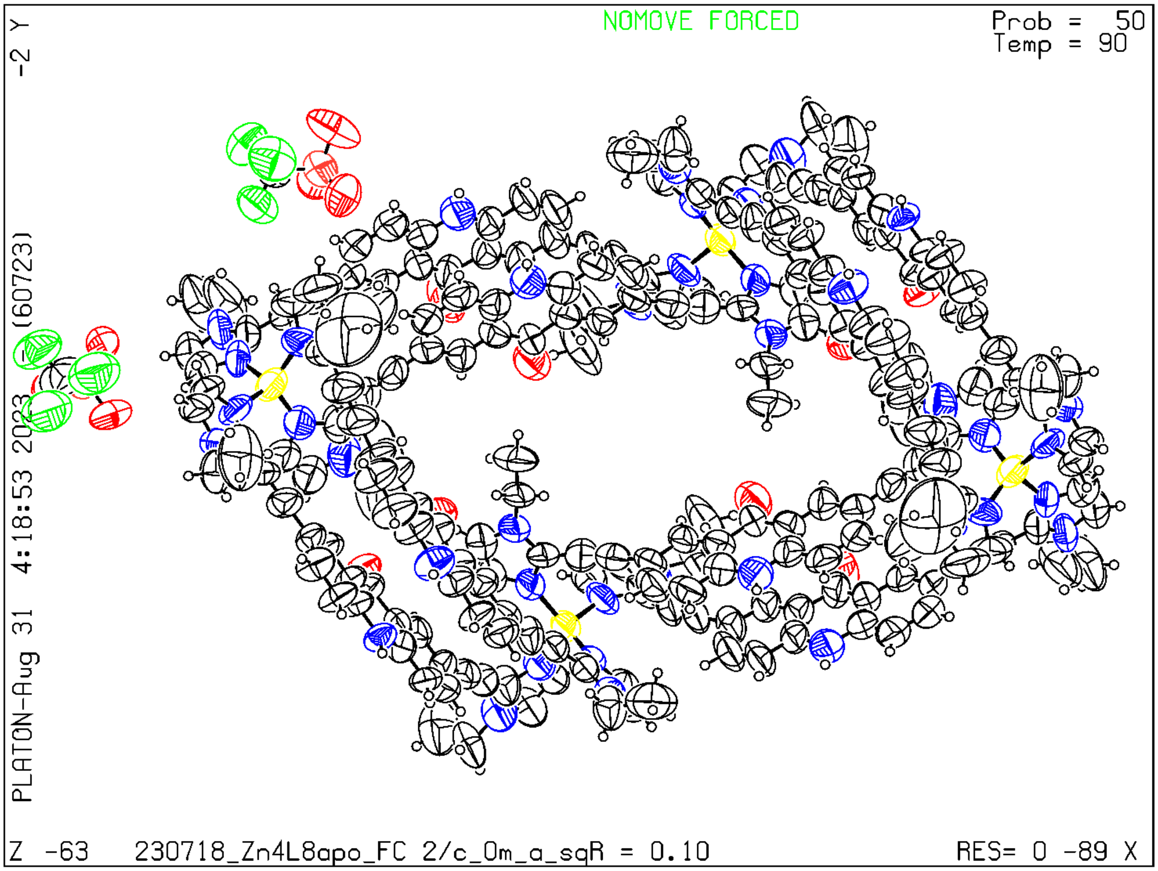

Supplement: Supplementary file 18 — Supplementary data file 16 [file 41467_2024_48599_MOESM18_ESM.pdf]
