# Supplementary material for: Direct observation of electron transfer in solids through X-ray crystallography
Source: Nat Commun. 2024 May 23;15:4412. doi: 10.1038/s41467-024-48599-1 (PMC11116525; doi:10.1038/s41467-024-48599-1)

checkCIF/PLATON report

Structure factors have been supplied for datablock(s) 220921_Zn4L8_Fer_0m_sqd_sqd

THIS REPORT IS FOR GUIDANCE ONLY. IF USED AS PART OF A REVIEW PROCEDURE FOR PUBLICATION, IT SHOULD NOT REPLACE THE EXPERTISE OF AN EXPERIENCED CRYSTALLOGRAPHIC REFEREE.

No syntax errors found. CIF dictionary Interpreting this report

Datablock: 220921_Zn4L8_Fer_0m_sqd_sqd

Bond precision: C-C = 0.0199 A Wavelength=0.71073

Cell:	a=56.597(2)	b=13.6900(6)	c=38.8777(15)
	alpha=90	beta=117.327(2)	gamma=90
Temperature:	90 K		

	Calculated	Reported
Volume	26761.3(19)	26761(2)
Space group	C 2/c	C 2/c
Hall group	-C 2yc	-C 2yc
Moiety formula	C216 H168 N40 O4 Zn4, 0.84(C10 H10 Fe), 4(C F3 O3 S) [+ solvent	?
Sum formula	C228.40 H176.40 F12 Fe0.84 N40 O16 S4 Zn4 [+ solvent]	C228.40 H176.40 F12 Fe0.84 N40 O16 S4 Zn4
Mr	4402.02	4401.92
Dx,g cm-3	1.093	1.093
Z	4	4
Mu (mm-1)	0.496	0.496
F000	9074.6	9075.0
F000'	9085.83	
h,k,lmax	58,14,40	58,14,40
Nref	15694	15643
Tmin,Tmax	0.868,0.927	0.659,0.745
Tmin'	0.822	

Correction method= # Reported T Limits: Tmin=0.659 Tmax=0.745
AbsCorr = MULTI-SCAN

Data completeness= 0.997 Theta(max)= 21.653

R(reflections)= 0.1309(8519)

wR2(reflections)= 0.3881(15643)

S = 1.329 Npar= 1442

The following ALERTS were generated. Each ALERT has the format
test-name_ALERT_alert-type_alert-level.
Click on the hyperlinks for more details of the test.

Alert level A

THETM01_ALERT_3_A The value of sine(theta_max)/wavelength is less than 0.550
Calculated sin(theta_max)/wavelength = 0.5192

Author Response: Despite long exposure times and rapid sample handling, few reflections at greater than 0.97 angstroms resolution were observed.

Alert level B

PLAT084_ALERT_3_B High wR2 Value (i.e. > 0.25) .... 0.39 Report

Author Response: The diffraction data of the crystals are not well observed and Fc molecules are weakly trapped in the crystals, giving the large thermal ellipsoids. As a result, the R1 and wR2 values are high.

PLAT230_ALERT_2_B Hirshfeld Test Diff for O1 --C68 . 16.3 s.u.

Author Response: This alert is because the relative positions of LA and LA=O in [(Zn2+)4(LA)4(LA=O)4]n are partially replaced. However, these have no significant impact on the double wall structure.

PLAT242_ALERT_2_B Low 'MainMol' Ueq as Compared to Neighbors of C68 Check

Author Response: This alert is because ethyl groups of the ligands are partially disordered by thermal vibration or the relative positions of LA and LA=O in [(Zn2+)4(LA)4(LA=O)4]n are partially replaced. However, these have no significant impact on the double wall structure.

PLAT341_ALERT_3_B Low Bond Precision on C-C Bonds .... 0.01989 Ang.

Author Response: The low bond precision arises from the limited resolution of the data.

PLAT934_ALERT_3_B Number of (Iobs-Icalc)/Sigma(W) > 10 Outliers .. 4 Check
PLAT990_ALERT_1_B Deprecated .res/.hkl Input Style SQUEEZE Job ... ! Note

---

Alert level C

```
ABSTY02_ALERT_1_C  An _exptl_absorpt_correction_type has been given without
            a literature citation. This should be contained in the
            _exptl_absorpt_process_details field.
            Absorption correction given as Multi-Scan
PLAT077_ALERT_4_C Unitcell Contains Non-integer Number of Atoms ..     Please Check
PLAT082_ALERT_2_C High R1 Value .................................       0.13 Report
PLAT213_ALERT_2_C Atom N18             has ADP max/min Ratio .....        3.1 prolat
PLAT220_ALERT_2_C NonSolvent   Resd 1  C   Ueq(max)/Ueq(min) Range        5.9 Ratio
PLAT222_ALERT_3_C NonSolvent Resd 1  H   Uiso(max)/Uiso(min) Range        7.3 Ratio
PLAT230_ALERT_2_C Hirshfeld Test Diff for    O2       --C95      .        5.4 s.u.
```

Author Response: This alert is because the relative positions of LA and LA=O in [(Zn2+)4(LA)4(LA=O)4]n are partially replaced. However, these have no significant impact on the double wall structure.

```
PLAT230_ALERT_2_C Hirshfeld Test Diff for    N2       --C3       .        6.7 s.u.
```

Author Response: This alert is because the relative positions of LA and LA=O in [(Zn2+)4(LA)4(LA=O)4]n are partially replaced. However, these have no significant impact on the double wall structure.

```
PLAT230_ALERT_2_C Hirshfeld Test Diff for    N12      --C56      .        5.9 s.u.
```

Author Response: This alert is because the relative positions of LA and LA=O in [(Zn2+)4(LA)4(LA=O)4]n are partially replaced. However, these have no significant impact on the double wall structure.

```
PLAT230_ALERT_2_C Hirshfeld Test Diff for    C62      --C67      .        5.2 s.u.
```

Author Response: This alert is because the relative positions of LA and LA=O in [(Zn2+)4(LA)4(LA=O)4]n are partially replaced. However, these have no significant impact on the double wall structure.

```
PLAT230_ALERT_2_C Hirshfeld Test Diff for    C89      --C94      .        6.2 s.u.
```

Author Response: This alert is because the relative positions of LA and LA=O in [(Zn2+)4(LA)4(LA=O)4]n are partially replaced. However, these have no significant impact on the double wall structure.

```
PLAT230_ALERT_2_C Hirshfeld Test Diff for    C91      --C92      .        5.5 s.u.
```

Author Response: This alert is because the relative positions of LA and LA=O in [(Zn2+)4(LA)4(LA=O)4]n are partially replaced. However, these have no significant impact on the double wall structure.

```
PLAT231_ALERT_4_C Hirshfeld Test (Solvent)   S1       --O5       .        7.6 s.u.
PLAT231_ALERT_4_C Hirshfeld Test (Solvent)   S2       --O7       .        8.1 s.u.
PLAT234_ALERT_4_C Large Hirshfeld Difference Zn2A     --N19_a    .       0.23 Ang.
PLAT234_ALERT_4_C Large Hirshfeld Difference N1       --C5       .       0.18 Ang.
PLAT234_ALERT_4_C Large Hirshfeld Difference N3       --C11      .       0.19 Ang.
PLAT234_ALERT_4_C Large Hirshfeld Difference N5       --C26      .       0.25 Ang.
PLAT234_ALERT_4_C Large Hirshfeld Difference N6       --C32      .       0.19 Ang.
PLAT234_ALERT_4_C Large Hirshfeld Difference N8       --C38      .       0.19 Ang.
PLAT234_ALERT_4_C Large Hirshfeld Difference N10      --C50      .       0.23 Ang.
PLAT234_ALERT_4_C Large Hirshfeld Difference N11      --C58      .       0.22 Ang.
PLAT234_ALERT_4_C Large Hirshfeld Difference N12      --C59      .       0.16 Ang.
PLAT234_ALERT_4_C Large Hirshfeld Difference N16      --C86      .       0.19 Ang.
PLAT234_ALERT_4_C Large Hirshfeld Difference N17      --C83      .       0.22 Ang.
PLAT234_ALERT_4_C Large Hirshfeld Difference N20      --C104     .       0.20 Ang.
PLAT234_ALERT_4_C Large Hirshfeld Difference N20      --C106     .       0.24 Ang.
PLAT234_ALERT_4_C Large Hirshfeld Difference C9       --C10      .       0.19 Ang.
PLAT234_ALERT_4_C Large Hirshfeld Difference C10      --C11      .       0.20 Ang.
PLAT234_ALERT_4_C Large Hirshfeld Difference C11      --C12      .       0.23 Ang.
PLAT234_ALERT_4_C Large Hirshfeld Difference C12      --C13      .       0.20 Ang.
PLAT234_ALERT_4_C Large Hirshfeld Difference C12      --C14      .       0.22 Ang.
PLAT234_ALERT_4_C Large Hirshfeld Difference C16      --C17      .       0.16 Ang.
PLAT234_ALERT_4_C Large Hirshfeld Difference C17      --C18      .       0.19 Ang.
PLAT234_ALERT_4_C Large Hirshfeld Difference C19      --C21      .       0.19 Ang.
PLAT234_ALERT_4_C Large Hirshfeld Difference C34      --C35      .       0.25 Ang.
PLAT234_ALERT_4_C Large Hirshfeld Difference C35      --C36      .       0.17 Ang.
PLAT234_ALERT_4_C Large Hirshfeld Difference C35      --C40      .       0.21 Ang.
PLAT234_ALERT_4_C Large Hirshfeld Difference C39      --C41      .       0.23 Ang.
PLAT234_ALERT_4_C Large Hirshfeld Difference C44      --C45      .       0.22 Ang.
PLAT234_ALERT_4_C Large Hirshfeld Difference C45      --C48      .       0.20 Ang.
PLAT234_ALERT_4_C Large Hirshfeld Difference C48      --C49      .       0.23 Ang.
PLAT234_ALERT_4_C Large Hirshfeld Difference C51      --C52      .       0.22 Ang.
PLAT234_ALERT_4_C Large Hirshfeld Difference C61      --C62      .       0.18 Ang.
PLAT234_ALERT_4_C Large Hirshfeld Difference C68      --C69      .       0.19 Ang.
PLAT234_ALERT_4_C Large Hirshfeld Difference C70      --C71      .       0.23 Ang.
PLAT234_ALERT_4_C Large Hirshfeld Difference C71      --C72      .       0.16 Ang.
PLAT234_ALERT_4_C Large Hirshfeld Difference C71      --C75      .       0.16 Ang.
PLAT234_ALERT_4_C Large Hirshfeld Difference C76      --C77      .       0.22 Ang.
PLAT234_ALERT_4_C Large Hirshfeld Difference C78      --C79      .       0.17 Ang.
PLAT234_ALERT_4_C Large Hirshfeld Difference C82      --C83      .       0.20 Ang.
PLAT234_ALERT_4_C Large Hirshfeld Difference C84      --C85      .       0.19 Ang.
PLAT234_ALERT_4_C Large Hirshfeld Difference C93      --C94      .       0.19 Ang.
PLAT234_ALERT_4_C Large Hirshfeld Difference C97      --C98      .       0.20 Ang.
PLAT234_ALERT_4_C Large Hirshfeld Difference C98      --C102     .       0.21 Ang.
PLAT234_ALERT_4_C Large Hirshfeld Difference C102     --C103     .       0.25 Ang.
PLAT234_ALERT_4_C Large Hirshfeld Difference F1       --C1C      .       0.23 Ang.
PLAT241_ALERT_2_C High   'MainMol' Ueq as Compared to Neighbors of         N3 Check
PLAT241_ALERT_2_C High   'MainMol' Ueq as Compared to Neighbors of         N8 Check
PLAT241_ALERT_2_C High   'MainMol' Ueq as Compared to Neighbors of        N18 Check
PLAT241_ALERT_2_C High   'MainMol' Ueq as Compared to Neighbors of         C2 Check
PLAT241_ALERT_2_C High   'MainMol' Ueq as Compared to Neighbors of         C3 Check
PLAT241_ALERT_2_C High   'MainMol' Ueq as Compared to Neighbors of        C14 Check
PLAT241_ALERT_2_C High   'MainMol' Ueq as Compared to Neighbors of        C25 Check
PLAT241_ALERT_2_C High   'MainMol' Ueq as Compared to Neighbors of        C29 Check
PLAT241_ALERT_2_C High   'MainMol' Ueq as Compared to Neighbors of        C30 Check
PLAT241_ALERT_2_C High   'MainMol' Ueq as Compared to Neighbors of        C33 Check
PLAT241_ALERT_2_C High   'MainMol' Ueq as Compared to Neighbors of        C37 Check
PLAT241_ALERT_2_C High   'MainMol' Ueq as Compared to Neighbors of        C40 Check
```

```
PLAT241_ALERT_2_C High   'MainMol' Ueq as Compared to Neighbors of        C41 Check
PLAT241_ALERT_2_C High   'MainMol' Ueq as Compared to Neighbors of        C43 Check
PLAT241_ALERT_2_C High   'MainMol' Ueq as Compared to Neighbors of        C51 Check
PLAT241_ALERT_2_C High   'MainMol' Ueq as Compared to Neighbors of        C57 Check
PLAT241_ALERT_2_C High   'MainMol' Ueq as Compared to Neighbors of        C73 Check
PLAT241_ALERT_2_C High   'MainMol' Ueq as Compared to Neighbors of        C75 Check
PLAT241_ALERT_2_C High   'MainMol' Ueq as Compared to Neighbors of        C78 Check
PLAT241_ALERT_2_C High   'MainMol' Ueq as Compared to Neighbors of        C84 Check
PLAT241_ALERT_2_C High   'MainMol' Ueq as Compared to Neighbors of        C99 Check
PLAT241_ALERT_2_C High   'MainMol' Ueq as Compared to Neighbors of       C100 Check
PLAT241_ALERT_2_C High   'MainMol' Ueq as Compared to Neighbors of       C103 Check
PLAT241_ALERT_2_C High   'MainMol' Ueq as Compared to Neighbors of       C106 Check
PLAT242_ALERT_2_C Low    'MainMol' Ueq as Compared to Neighbors of         N2 Check
```

Author Response: This alert is because ethyl groups of the ligands are partially disordered by thermal vibration or the relative positions of LA and LA=O in [(Zn2+)4(LA)4(LA=O)4]n are partially replaced. However, these have no significant impact on the double wall structure.

```
PLAT242_ALERT_2_C Low    'MainMol' Ueq as Compared to Neighbors of         N7 Check
```

Author Response: This alert is because ethyl groups of the ligands are partially disordered by thermal vibration or the relative positions of LA and LA=O in [(Zn2+)4(LA)4(LA=O)4]n are partially replaced. However, these have no significant impact on the double wall structure.

```
PLAT242_ALERT_2_C Low    'MainMol' Ueq as Compared to Neighbors of         N9 Check
```

Author Response: This alert is because ethyl groups of the ligands are partially disordered by thermal vibration or the relative positions of LA and LA=O in [(Zn2+)4(LA)4(LA=O)4]n are partially replaced. However, these have no significant impact on the double wall structure.

```
PLAT242_ALERT_2_C Low    'MainMol' Ueq as Compared to Neighbors of        N10 Check
```

Author Response: This alert is because ethyl groups of the ligands are partially disordered by thermal vibration or the relative positions of LA and LA=O in [(Zn2+)4(LA)4(LA=O)4]n are partially replaced. However, these have no significant impact on the double wall structure.

```
PLAT242_ALERT_2_C Low    'MainMol' Ueq as Compared to Neighbors of        N15 Check
```

Author Response: This alert is because ethyl groups of the ligands are partially disordered by thermal vibration or the relative positions of LA and LA=O in [(Zn2+)4(LA)4(LA=O)4]n are partially replaced. However, these have no significant impact on the double wall structure.

PLAT242_ALERT_2_C Low   'MainMol' Ueq as Compared to Neighbors of   N19 Check

Author Response: This alert is because ethyl groups of the ligands are partially disordered by thermal vibration or the relative positions of LA and LA=O in [(Zn2+)4(LA)4(LA=O)4]n are partially replaced. However, these have no significant impact on the double wall structure.

PLAT242_ALERT_2_C Low   'MainMol' Ueq as Compared to Neighbors of   N20 Check

Author Response: This alert is because ethyl groups of the ligands are partially disordered by thermal vibration or the relative positions of LA and LA=O in [(Zn2+)4(LA)4(LA=O)4]n are partially replaced. However, these have no significant impact on the double wall structure.

PLAT242_ALERT_2_C Low   'MainMol' Ueq as Compared to Neighbors of   C26 Check

Author Response: This alert is because ethyl groups of the ligands are partially disordered by thermal vibration or the relative positions of LA and LA=O in [(Zn2+)4(LA)4(LA=O)4]n are partially replaced. However, these have no significant impact on the double wall structure.

PLAT242_ALERT_2_C Low   'MainMol' Ueq as Compared to Neighbors of   C35 Check

Author Response: This alert is because ethyl groups of the ligands are partially disordered by thermal vibration or the relative positions of LA and LA=O in [(Zn2+)4(LA)4(LA=O)4]n are partially replaced. However, these have no significant impact on the double wall structure.

PLAT242_ALERT_2_C Low   'MainMol' Ueq as Compared to Neighbors of   C39 Check

Author Response: This alert is because ethyl groups of the ligands are partially disordered by thermal vibration or the relative positions of LA and LA=O in [(Zn2+)4(LA)4(LA=O)4]n are partially replaced. However, these have no significant impact on the double wall structure.

PLAT242_ALERT_2_C Low   'MainMol' Ueq as Compared to Neighbors of   C42 Check

Author Response: This alert is because ethyl groups of the ligands are partially disordered by thermal vibration or the relative positions of LA and LA=O in [(Zn2+)4(LA)4(LA=O)4]n are partially replaced. However, these have no significant impact on the double wall structure.

```
PLAT242_ALERT_2_C Low    'MainMol' Ueq as Compared to Neighbors of        C53 Check
```

Author Response: This alert is because ethyl groups of the ligands are partially disordered by thermal vibration or the relative positions of LA and LA=O in [(Zn2+)4(LA)4(LA=O)4]n are partially replaced. However, these have no significant impact on the double wall structure.

```
PLAT242_ALERT_2_C Low    'MainMol' Ueq as Compared to Neighbors of        C80 Check
```

Author Response: This alert is because ethyl groups of the ligands are partially disordered by thermal vibration or the relative positions of LA and LA=O in [(Zn2+)4(LA)4(LA=O)4]n are partially replaced. However, these have no significant impact on the double wall structure.

```
PLAT242_ALERT_2_C Low    'MainMol' Ueq as Compared to Neighbors of        C91 Check
```

Author Response: This alert is because ethyl groups of the ligands are partially disordered by thermal vibration or the relative positions of LA and LA=O in [(Zn2+)4(LA)4(LA=O)4]n are partially replaced. However, these have no significant impact on the double wall structure.

```
PLAT242_ALERT_2_C Low    'MainMol' Ueq as Compared to Neighbors of        C97 Check
```

Author Response: This alert is because ethyl groups of the ligands are partially disordered by thermal vibration or the relative positions of LA and LA=O in [(Zn2+)4(LA)4(LA=O)4]n are partially replaced. However, these have no significant impact on the double wall structure.

```
PLAT242_ALERT_2_C Low    'MainMol' Ueq as Compared to Neighbors of       C101 Check
```

Author Response: This alert is because ethyl groups of the ligands are partially disordered by thermal vibration or the relative positions of LA and LA=O in [(Zn2+)4(LA)4(LA=O)4]n are partially replaced. However, these have no significant impact on the double wall structure.

```
PLAT244_ALERT_4_C Low    'Solvent' Ueq as Compared to Neighbors of         S1 Check
PLAT244_ALERT_4_C Low    'Solvent' Ueq as Compared to Neighbors of         S2 Check
PLAT250_ALERT_2_C Large U3/U1 Ratio for Average U(i,j) Tensor ....        2.3 Note
PLAT260_ALERT_2_C Large Average Ueq of Residue Including       Zn1      0.144 Check
PLAT260_ALERT_2_C Large Average Ueq of Residue Including       Fe1      0.282 Check
PLAT260_ALERT_2_C Large Average Ueq of Residue Including        S1      0.181 Check
PLAT260_ALERT_2_C Large Average Ueq of Residue Including        S2      0.183 Check
PLAT334_ALERT_2_C Small <C-C> Benzene Dist.   C96      -C101     .       1.37 Ang.
PLAT360_ALERT_2_C Short  C(sp3)-C(sp3) Bond  C28      - C29      .       1.39 Ang.
PLAT360_ALERT_2_C Short  C(sp3)-C(sp3) Bond  C55      - C56      .       1.41 Ang.
PLAT420_ALERT_2_C D-H Bond Without Acceptor  N13      --H13A     .     Please Check
PLAT420_ALERT_2_C D-H Bond Without Acceptor  N18      --H18A     .     Please Check
```

```
PLAT767_ALERT_4_C INS Embedded LIST 6 Instruction Should be LIST 4     Please Check
PLAT906_ALERT_3_C Large K Value in the Analysis of Variance ......     21.385 Check
PLAT906_ALERT_3_C Large K Value in the Analysis of Variance ......      4.460 Check
PLAT906_ALERT_3_C Large K Value in the Analysis of Variance ......      2.108 Check
PLAT910_ALERT_3_C Missing # of FCF Reflection(s) Below Theta(Min).          8 Note
PLAT911_ALERT_3_C Missing FCF Refl Between Thmin & STh/L=    0.519         43 Report
PLAT918_ALERT_3_C Reflection(s) with I(obs) much Smaller I(calc) .          8 Check
PLAT922_ALERT_1_C wR2 in the CIF and FCF Differ by ..............      0.0014 Check
```

Alert level G

```
PLAT002_ALERT_2_G Number of Distance or Angle Restraints on AtSite         39 Note
PLAT003_ALERT_2_G Number of Uiso or Uij Restrained non-H Atoms ...         42 Report
PLAT007_ALERT_5_G Number of Unrefined Donor-H Atoms .............          2 Report
PLAT128_ALERT_4_G Alternate Setting for Input Space Group     C2/c       I2/a Note
PLAT172_ALERT_4_G The CIF-Embedded .res File Contains DFIX Records         11 Report
PLAT173_ALERT_4_G The CIF-Embedded .res File Contains DANG Records          9 Report
PLAT178_ALERT_4_G The CIF-Embedded .res File Contains SIMU Records         10 Report
PLAT186_ALERT_4_G The CIF-Embedded .res File Contains ISOR Records          2 Report
PLAT187_ALERT_4_G The CIF-Embedded .res File Contains RIGU Records          9 Report
PLAT188_ALERT_3_G A Non-default SIMU Restraint Value has been used     0.0200 Report
PLAT188_ALERT_3_G A Non-default SIMU Restraint Value has been used     0.0200 Report
PLAT188_ALERT_3_G A Non-default SIMU Restraint Value has been used     0.0200 Report
PLAT188_ALERT_3_G A Non-default SIMU Restraint Value has been used     0.0200 Report
PLAT232_ALERT_2_G Hirshfeld Test Diff (M-X)  Zn2      --N1       .        9.0 s.u.
PLAT232_ALERT_2_G Hirshfeld Test Diff (M-X)  Zn2      --N6       .        7.4 s.u.
PLAT232_ALERT_2_G Hirshfeld Test Diff (M-X)  Zn2      --N19_a    .       15.8 s.u.
PLAT244_ALERT_4_G Low    'Solvent' Ueq as Compared to Neighbors of        C1C Check
PLAT244_ALERT_4_G Low    'Solvent' Ueq as Compared to Neighbors of        C2C Check
PLAT301_ALERT_3_G Main Residue  Disorder .............(Resd  1  )          1% Note
PLAT302_ALERT_4_G Anion/Solvent/Minor-Residue Disorder (Resd  2  )       100% Note
PLAT304_ALERT_4_G Non-Integer Number of Atoms in ..... (Resd  2  )       8.82 Check
PLAT335_ALERT_2_G Check Large C6 Ring C-C Range C15      -C20            0.18 Ang.
PLAT335_ALERT_2_G Check Large C6 Ring C-C Range C89      -C94            0.16 Ang.
PLAT343_ALERT_2_G Unusual sp3      Angle Range in Main Residue for        C29 Check
PLAT371_ALERT_2_G Long   C(sp2)-C(sp1) Bond  C5       - C6       .       1.45 Ang.
PLAT371_ALERT_2_G Long   C(sp2)-C(sp1) Bond  C45      - C48      .       1.52 Ang.
PLAT371_ALERT_2_G Long   C(sp2)-C(sp1) Bond  C61      - C62      .       1.45 Ang.
PLAT371_ALERT_2_G Long   C(sp2)-C(sp1) Bond  C76      - C77      .       1.41 Ang.
PLAT371_ALERT_2_G Long   C(sp2)-C(sp1) Bond  C86      - C87      .       1.44 Ang.
PLAT371_ALERT_2_G Long   C(sp2)-C(sp1) Bond  C88      - C89      .       1.45 Ang.
PLAT371_ALERT_2_G Long   C(sp2)-C(sp1) Bond  C98      - C102     .       1.53 Ang.
PLAT606_ALERT_4_G Solvent Accessible VOID(S) in Structure ........          ! Info
PLAT779_ALERT_4_G Suspect or Irrelevant (Bond) Angle(s) in CIF ...       2.90 Deg.
              ZN2  -N19  -ZN2A    2_655   1_555   2_655 ......     #  268 Check
PLAT794_ALERT_5_G Tentative Bond Valency for Zn1       (II)      .       1.90 Info
PLAT802_ALERT_4_G CIF Input Record(s) with more than 80 Characters          2 Info
PLAT860_ALERT_3_G Number of Least-Squares Restraints ............         391 Note
PLAT869_ALERT_4_G ALERTS Related to the Use of SQUEEZE  Suppressed          ! Info
PLAT883_ALERT_1_G No Info/Value for _atom_sites_solution_primary .     Please Do !
PLAT913_ALERT_3_G Missing # of Very Strong Reflections in FCF ....          1 Note
PLAT961_ALERT_5_G Dataset Contains no Negative Intensities .......     Please Check
PLAT978_ALERT_2_G Number C-C Bonds with Positive Residual Density.          0 Info
```

---

1 **ALERT level A** = Most likely a serious problem - resolve or explain
6 **ALERT level B** = A potentially serious problem, consider carefully

```
117 ALERT level C = Check. Ensure it is not caused by an omission or oversight
 41 ALERT level G = General information/check it is not something unexpected

  4 ALERT type 1 CIF construction/syntax error, inconsistent or missing data
 77 ALERT type 2 Indicator that the structure model may be wrong or deficient
 18 ALERT type 3 Indicator that the structure quality may be low
 63 ALERT type 4 Improvement, methodology, query or suggestion
  3 ALERT type 5 Informative message, check
```

It is advisable to attempt to resolve as many as possible of the alerts in all categories. Often the minor alerts point to easily fixed oversights, errors and omissions in your CIF or refinement strategy, so attention to these fine details can be worthwhile. In order to resolve some of the more serious problems it may be necessary to carry out additional measurements or structure refinements. However, the purpose of your study may justify the reported deviations and the more serious of these should normally be commented upon in the discussion or experimental section of a paper or in the "special_details" fields of the CIF. checkCIF was carefully designed to identify outliers and unusual parameters, but every test has its limitations and alerts that are not important in a particular case may appear. Conversely, the absence of alerts does not guarantee there are no aspects of the results needing attention. It is up to the individual to critically assess their own results and, if necessary, seek expert advice.

Publication of your CIF in IUCr journals

A basic structural check has been run on your CIF. These basic checks will be run on all CIFs submitted for publication in IUCr journals (*Acta Crystallographica*, *Journal of Applied Crystallography*, *Journal of Synchrotron Radiation*); however, if you intend to submit to *Acta Crystallographica Section C* or *E* or *IUCrData*, you should make sure that full publication checks are run on the final version of your CIF prior to submission.

Publication of your CIF in other journals

Please refer to the *Notes for Authors* of the relevant journal for any special instructions relating to CIF submission.

PLATON version of 06/07/2023; check.def file version of 30/06/2023

Datablock 220921_Zn4L8_Fer_0m_sqd_sqd - ellipsoid plot

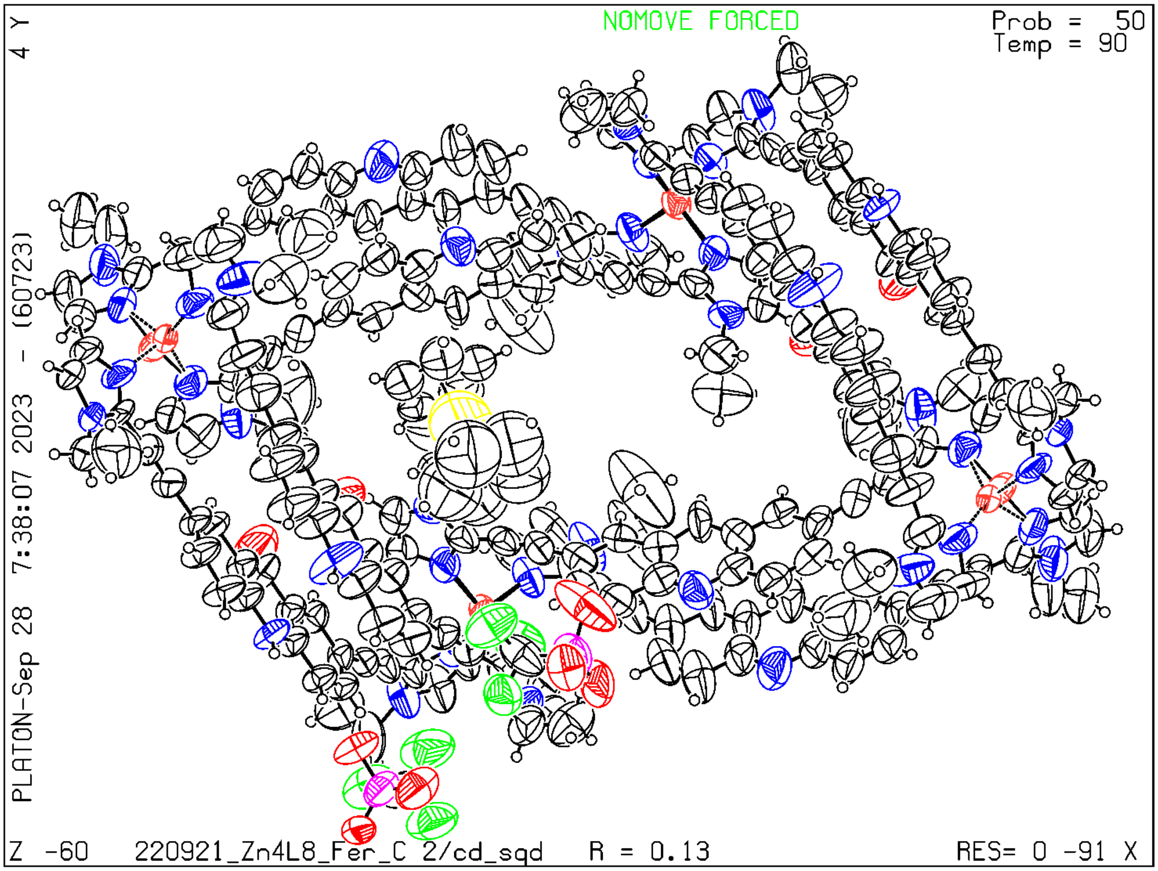

Supplement: Supplementary file 21 — Supplementary data file 18 [file 41467_2024_48599_MOESM21_ESM.pdf]
