# Supplementary material for: Direct observation of electron transfer in solids through X-ray crystallography
Source: Nat Commun. 2024 May 23;15:4412. doi: 10.1038/s41467-024-48599-1 (PMC11116525; doi:10.1038/s41467-024-48599-1)

checkCIF/PLATON report

Structure factors have been supplied for datablock(s) 230707_TTFZn4L8_FeClO4_oxisoli_sqd_sqd

THIS REPORT IS FOR GUIDANCE ONLY. IF USED AS PART OF A REVIEW PROCEDURE FOR PUBLICATION, IT SHOULD NOT REPLACE THE EXPERTISE OF AN EXPERIENCED CRYSTALLOGRAPHIC REFEREE.

No syntax errors found. CIF dictionary Interpreting this report

Datablock: 230707_TTFZn4L8_FeClO4_oxisoli_sqd_sqd

```
Bond precision:   C-C = 0.0263 A          Wavelength=0.71073

Cell:             a=56.592(3)    b=13.8354(8)       c=38.8581(18)
                  alpha=90       beta=117.121(2)    gamma=90
Temperature:      90 K
```

	Calculated	Reported
Volume	27080(3)	27080(2)
Space group	C 2/c	C 2/c
Hall group	-C 2yc	-C 2yc
Moiety formula	C216 H168 N40 O8 Zn4, 1.258(C6 H4 S4), 4(C F3 O3 S), 2(Cl O4) [	?
Sum formula	C227.55 H173.03 Cl2 F12 N40 O28 S9.03 Zn4 [+ solvent]	C227.56 H173.03 Cl2 F12 N40 O28 S9.03 Zn4
Mr	4765.71	4765.79
Dx,g cm-3	1.169	1.169
Z	4	4
Mu (mm-1)	0.512	0.512
F000	9795.3	9796.0
F000'	9809.64	
h,k,lmax	52,12,36	52,12,36
Nref	11490	11403
Tmin,Tmax	0.853,0.934	0.600,0.744
Tmin'	0.783	

```
Correction method= # Reported T Limits: Tmin=0.600 Tmax=0.744
AbsCorr = MULTI-SCAN

Data completeness= 0.992            Theta(max)= 19.351
```

R(reflections)= 0.1418(6347)

wR2(reflections)= 0.4004(11403)

S = 1.385 Npar= 1497

The following ALERTS were generated. Each ALERT has the format
test-name_ALERT_alert-type_alert-level.
Click on the hyperlinks for more details of the test.

Alert level A

THETM01_ALERT_3_A The value of sine(theta_max)/wavelength is less than 0.550
Calculated sin(theta_max)/wavelength = 0.4662

Author Response: Despite long exposure times and rapid sample handling, few reflections at greater than 1.08 angstroms resolution were observed.

Alert level B

PLAT084_ALERT_3_B High wR2 Value (i.e. > 0.25) .... 0.40 Report

Author Response: The diffraction data of the crystals are not well observed due to the reduced quality of the crystals, giving the large thermal ellipsoids. As a result, the R1 and wR2 values are high.

PLAT088_ALERT_3_B Poor Data / Parameter Ratio .... 7.62 Note

Author Response: The low data to parameter ratio results from the poor diffraction of the crystal.

PLAT242_ALERT_2_B Low 'MainMol' Ueq as Compared to Neighbors of C81 Check

Author Response: This alert is because ethyl groups of the ligands are partially disordered by thermal vibration.

PLAT242_ALERT_2_B Low 'MainMol' Ueq as Compared to Neighbors of C107 Check

Author Response: This alert is because ethyl groups of the ligands are partially disordered by thermal vibration.

PLAT260_ALERT_2_B Large Average Ueq of Residue Including S1A 0.545 Check

Author Response: The diffraction data of the crystals are not well observed due to the reduced quality of the crystals.

```
PLAT260_ALERT_2_B Large Average Ueq of Residue Including       Cl1      0.819 Check
```

Author Response: The diffraction data of the crystals are not well observed due to the reduced quality of the crystals.

```
PLAT341_ALERT_3_B Low Bond Precision on  C-C Bonds ..............    0.02628 Ang.
```

Author Response: The low bond precision arises from the limited resolution of the data.

```
PLAT430_ALERT_2_B Short Inter D...A Contact  O1A       ..O2       .       2.78 Ang.
                                                  x,-1+y,z  =      1_545 Check
```

Author Response: The ClO4 anions show substantial thermal vibration which could not be refined with precise position.

```
PLAT990_ALERT_1_B Deprecated .res/.hkl Input Style SQUEEZE Job ...          ! Note
```

Alert level C

```
ABSTY02_ALERT_1_C  An _exptl_absorpt_correction_type has been given without
            a literature citation. This should be contained in the
            _exptl_absorpt_process_details field.
            Absorption correction given as Multi-Scan
PLAT082_ALERT_2_C High R1 Value ................................       0.14 Report
PLAT213_ALERT_2_C Atom N19             has ADP max/min Ratio .....       3.2 prolat
PLAT213_ALERT_2_C Atom C21             has ADP max/min Ratio .....       3.1 prolat
PLAT213_ALERT_2_C Atom C32             has ADP max/min Ratio .....       3.2 prolat
PLAT213_ALERT_2_C Atom C87             has ADP max/min Ratio .....       3.8 oblate
PLAT213_ALERT_2_C Atom C104            has ADP max/min Ratio .....       3.6 oblate
PLAT220_ALERT_2_C NonSolvent   Resd 1  C   Ueq(max)/Ueq(min) Range       6.0 Ratio
PLAT222_ALERT_3_C NonSolvent Resd 1  H   Uiso(max)/Uiso(min) Range       7.0 Ratio
PLAT230_ALERT_2_C Hirshfeld Test Diff for    O1       --C14      .       6.0 s.u.
PLAT230_ALERT_2_C Hirshfeld Test Diff for    N5       --C26      .       5.5 s.u.
PLAT230_ALERT_2_C Hirshfeld Test Diff for    C84      --C85      .       6.4 s.u.
PLAT230_ALERT_2_C Hirshfeld Test Diff for    C93      --C94      .       5.5 s.u.
PLAT231_ALERT_4_C Hirshfeld Test (Solvent)   S3       --O11      .       8.7 s.u.
PLAT232_ALERT_2_C Hirshfeld Test Diff (M-X)  Zn2      --N14_a    .       5.5 s.u.
PLAT234_ALERT_4_C Large Hirshfeld Difference Zn1      --N4       .      0.16 Ang.
PLAT234_ALERT_4_C Large Hirshfeld Difference Zn2      --N2       .      0.18 Ang.
PLAT234_ALERT_4_C Large Hirshfeld Difference Zn2      --N6       .      0.18 Ang.
PLAT234_ALERT_4_C Large Hirshfeld Difference O3       --C68      .      0.16 Ang.
PLAT234_ALERT_4_C Large Hirshfeld Difference O4       --C95      .      0.20 Ang.
PLAT234_ALERT_4_C Large Hirshfeld Difference N1       --C3       .      0.25 Ang.
PLAT234_ALERT_4_C Large Hirshfeld Difference N10      --C50      .      0.22 Ang.
PLAT234_ALERT_4_C Large Hirshfeld Difference N14      --C78      .      0.22 Ang.
PLAT234_ALERT_4_C Large Hirshfeld Difference N17      --C83      .      0.24 Ang.
PLAT234_ALERT_4_C Large Hirshfeld Difference N18      --C97      .      0.19 Ang.
PLAT234_ALERT_4_C Large Hirshfeld Difference N19      --C104     .      0.25 Ang.
PLAT234_ALERT_4_C Large Hirshfeld Difference N20      --C104     .      0.20 Ang.
PLAT234_ALERT_4_C Large Hirshfeld Difference N20      --C107     .      0.22 Ang.
PLAT234_ALERT_4_C Large Hirshfeld Difference C7       --C8       .      0.22 Ang.
PLAT234_ALERT_4_C Large Hirshfeld Difference C10      --C11      .      0.19 Ang.
```

```
PLAT234_ALERT_4_C Large Hirshfeld Difference C14      --C15      .       0.20 Ang.
PLAT234_ALERT_4_C Large Hirshfeld Difference C19      --C21      .       0.20 Ang.
PLAT234_ALERT_4_C Large Hirshfeld Difference C22      --C23      .       0.22 Ang.
PLAT234_ALERT_4_C Large Hirshfeld Difference C39      --C40      .       0.24 Ang.
PLAT234_ALERT_4_C Large Hirshfeld Difference C39      --C41      .       0.25 Ang.
PLAT234_ALERT_4_C Large Hirshfeld Difference C42      --C43      .       0.22 Ang.
PLAT234_ALERT_4_C Large Hirshfeld Difference C42      --C47      .       0.22 Ang.
PLAT234_ALERT_4_C Large Hirshfeld Difference C43      --C44      .       0.22 Ang.
PLAT234_ALERT_4_C Large Hirshfeld Difference C62      --C63      .       0.23 Ang.
PLAT234_ALERT_4_C Large Hirshfeld Difference C63      --C64      .       0.22 Ang.
PLAT234_ALERT_4_C Large Hirshfeld Difference C64      --C65      .       0.24 Ang.
PLAT234_ALERT_4_C Large Hirshfeld Difference C65      --C66      .       0.23 Ang.
PLAT234_ALERT_4_C Large Hirshfeld Difference C66      --C67      .       0.19 Ang.
PLAT234_ALERT_4_C Large Hirshfeld Difference C69      --C70      .       0.17 Ang.
PLAT234_ALERT_4_C Large Hirshfeld Difference C72      --C73      .       0.24 Ang.
PLAT234_ALERT_4_C Large Hirshfeld Difference C82      --C83      .       0.24 Ang.
PLAT234_ALERT_4_C Large Hirshfeld Difference C86      --C87      .       0.24 Ang.
PLAT234_ALERT_4_C Large Hirshfeld Difference C88      --C89      .       0.19 Ang.
PLAT234_ALERT_4_C Large Hirshfeld Difference C97      --C98      .       0.19 Ang.
PLAT234_ALERT_4_C Large Hirshfeld Difference S2       --O8       .       0.19 Ang.
PLAT234_ALERT_4_C Large Hirshfeld Difference S2       --O9       .       0.22 Ang.
PLAT234_ALERT_4_C Large Hirshfeld Difference S2       --O10      .       0.23 Ang.
PLAT234_ALERT_4_C Large Hirshfeld Difference F4       --C2B      .       0.24 Ang.
PLAT234_ALERT_4_C Large Hirshfeld Difference F6       --C2B      .       0.22 Ang.
PLAT234_ALERT_4_C Large Hirshfeld Difference S3       --O12      .       0.22 Ang.
PLAT234_ALERT_4_C Large Hirshfeld Difference S3       --C3B      .       0.21 Ang.
PLAT241_ALERT_2_C High   'MainMol' Ueq as Compared to Neighbors of        N3 Check
PLAT241_ALERT_2_C High   'MainMol' Ueq as Compared to Neighbors of       N13 Check
PLAT241_ALERT_2_C High   'MainMol' Ueq as Compared to Neighbors of       C12 Check
PLAT241_ALERT_2_C High   'MainMol' Ueq as Compared to Neighbors of       C17 Check
PLAT241_ALERT_2_C High   'MainMol' Ueq as Compared to Neighbors of       C25 Check
PLAT241_ALERT_2_C High   'MainMol' Ueq as Compared to Neighbors of       C34 Check
PLAT241_ALERT_2_C High   'MainMol' Ueq as Compared to Neighbors of       C64 Check
PLAT241_ALERT_2_C High   'MainMol' Ueq as Compared to Neighbors of       C71 Check
PLAT241_ALERT_2_C High   'MainMol' Ueq as Compared to Neighbors of       C72 Check
PLAT241_ALERT_2_C High   'MainMol' Ueq as Compared to Neighbors of       C74 Check
PLAT241_ALERT_2_C High   'MainMol' Ueq as Compared to Neighbors of       C79 Check
PLAT241_ALERT_2_C High   'MainMol' Ueq as Compared to Neighbors of       C84 Check
PLAT241_ALERT_2_C High   'MainMol' Ueq as Compared to Neighbors of      C105 Check
PLAT241_ALERT_2_C High   'MainMol' Ueq as Compared to Neighbors of      C106 Check
PLAT242_ALERT_2_C Low    'MainMol' Ueq as Compared to Neighbors of        N2 Check
```

Author Response: This alert is because ethyl groups of the ligands are partially disordered by thermal vibration.

```
PLAT242_ALERT_2_C Low    'MainMol' Ueq as Compared to Neighbors of        N5 Check
```

Author Response: This alert is because ethyl groups of the ligands are partially disordered by thermal vibration.

```
PLAT242_ALERT_2_C Low    'MainMol' Ueq as Compared to Neighbors of       N14 Check
```

Author Response: This alert is because ethyl groups of the ligands are partially disordered by thermal vibration.

```
PLAT242_ALERT_2_C Low    'MainMol' Ueq as Compared to Neighbors of        N20 Check
```

Author Response: This alert is because ethyl groups of the ligands are partially disordered by thermal vibration.

```
PLAT242_ALERT_2_C Low    'MainMol' Ueq as Compared to Neighbors of         C2 Check
```

Author Response: This alert is because ethyl groups of the ligands are partially disordered by thermal vibration.

```
PLAT242_ALERT_2_C Low    'MainMol' Ueq as Compared to Neighbors of        C10 Check
```

Author Response: This alert is because ethyl groups of the ligands are partially disordered by thermal vibration.

```
PLAT242_ALERT_2_C Low    'MainMol' Ueq as Compared to Neighbors of        C11 Check
```

Author Response: This alert is because ethyl groups of the ligands are partially disordered by thermal vibration.

```
PLAT242_ALERT_2_C Low    'MainMol' Ueq as Compared to Neighbors of        C14 Check
```

Author Response: This alert is because ethyl groups of the ligands are partially disordered by thermal vibration.

```
PLAT242_ALERT_2_C Low    'MainMol' Ueq as Compared to Neighbors of        C26 Check
```

Author Response: This alert is because ethyl groups of the ligands are partially disordered by thermal vibration.

```
PLAT242_ALERT_2_C Low    'MainMol' Ueq as Compared to Neighbors of        C29 Check
```

Author Response: This alert is because ethyl groups of the ligands are partially disordered by thermal vibration.

```
PLAT242_ALERT_2_C Low    'MainMol' Ueq as Compared to Neighbors of        C35 Check
```

Author Response: This alert is because ethyl groups of the ligands are partially disordered by thermal vibration.

PLAT242_ALERT_2_C Low 'MainMol' Ueq as Compared to Neighbors of C41 Check

Author Response: This alert is because ethyl groups of the ligands are partially disordered by thermal vibration.

PLAT242_ALERT_2_C Low 'MainMol' Ueq as Compared to Neighbors of C42 Check

Author Response: This alert is because ethyl groups of the ligands are partially disordered by thermal vibration.

PLAT242_ALERT_2_C Low 'MainMol' Ueq as Compared to Neighbors of C53 Check

Author Response: This alert is because ethyl groups of the ligands are partially disordered by thermal vibration.

PLAT242_ALERT_2_C Low 'MainMol' Ueq as Compared to Neighbors of C70 Check

Author Response: This alert is because ethyl groups of the ligands are partially disordered by thermal vibration.

PLAT242_ALERT_2_C Low 'MainMol' Ueq as Compared to Neighbors of C73 Check

Author Response: This alert is because ethyl groups of the ligands are partially disordered by thermal vibration.

PLAT242_ALERT_2_C Low 'MainMol' Ueq as Compared to Neighbors of C83 Check

Author Response: This alert is because ethyl groups of the ligands are partially disordered by thermal vibration.

PLAT242_ALERT_2_C Low 'MainMol' Ueq as Compared to Neighbors of C85 Check

Author Response: This alert is because ethyl groups of the ligands are partially disordered by thermal vibration.

PLAT242_ALERT_2_C Low 'MainMol' Ueq as Compared to Neighbors of C95 Check

Author Response: This alert is because ethyl groups of the ligands are partially disordered by thermal vibration.

```
PLAT243_ALERT_4_C High   'Solvent' Ueq as Compared to Neighbors of        Cl1 Check
PLAT244_ALERT_4_C Low    'Solvent' Ueq as Compared to Neighbors of         S2 Check
PLAT244_ALERT_4_C Low    'Solvent' Ueq as Compared to Neighbors of         S3 Check
PLAT250_ALERT_2_C Large U3/U1 Ratio for Average U(i,j) Tensor ....        2.5 Note
PLAT250_ALERT_2_C Large U3/U1 Ratio for Average U(i,j) Tensor ....        2.4 Note
PLAT260_ALERT_2_C Large Average Ueq of Residue Including       Zn1      0.164 Check
```

Author Response: The diffraction data of the crystals are not well observed due to the reduced quality of the crystals.

```
PLAT260_ALERT_2_C Large Average Ueq of Residue Including        S2      0.196 Check
```

Author Response: The diffraction data of the crystals are not well observed due to the reduced quality of the crystals.

```
PLAT260_ALERT_2_C Large Average Ueq of Residue Including        S3      0.216 Check
```

Author Response: The diffraction data of the crystals are not well observed due to the reduced quality of the crystals.

```
PLAT334_ALERT_2_C Small <C-C> Benzene Dist.   C42      -C47      .       1.37 Ang.
PLAT334_ALERT_2_C Small <C-C> Benzene Dist.   C69      -C74      .       1.37 Ang.
PLAT372_ALERT_2_C Short  C(sp)-C(sp)   Bond  C21      - C22      .       1.10 Ang.
PLAT372_ALERT_2_C Short  C(sp)-C(sp)   Bond  C87      - C88      .       1.09 Ang.
PLAT420_ALERT_2_C D-H Bond Without Acceptor  N3       --H3Z      .     Please Check
PLAT420_ALERT_2_C D-H Bond Without Acceptor  N13      --H13Z     .     Please Check
PLAT420_ALERT_2_C D-H Bond Without Acceptor  N18      --H18Z     .     Please Check
PLAT767_ALERT_4_C INS Embedded LIST 6 Instruction Should be LIST 4     Please Check
PLAT906_ALERT_3_C Large K Value in the Analysis of Variance ......     33.671 Check
PLAT906_ALERT_3_C Large K Value in the Analysis of Variance ......      5.939 Check
PLAT906_ALERT_3_C Large K Value in the Analysis of Variance ......      2.472 Check
PLAT910_ALERT_3_C Missing # of FCF Reflection(s) Below Theta(Min).          5 Note
PLAT911_ALERT_3_C Missing FCF Refl Between Thmin & STh/L=    0.466         83 Report
PLAT918_ALERT_3_C Reflection(s) with I(obs) much Smaller I(calc) .          4 Check
PLAT934_ALERT_3_C Number of (Iobs-Icalc)/Sigma(W) > 10 Outliers ..          1 Check
```

Alert level G

```
FORMU01_ALERT_2_G  There is a discrepancy between the atom counts in the
            _chemical_formula_sum and the formula from the _atom_site* data.
            Atom count from _chemical_formula_sum:C227.56 H173.03 Cl2 F12 N40 O28
            Atom count from the _atom_site data:  C227.5480 H173.0319 Cl2 F12 N40
CELLZ01_ALERT_1_G Difference between formula and atom_site contents detected.
CELLZ01_ALERT_1_G ALERT: check formula stoichiometry or atom site occupancies.
           From the CIF: _cell_formula_units_Z    4
           From the CIF: _chemical_formula_sum  C227.56 H173.03 Cl2 F12 N40 O28 S9
           TEST: Compare cell contents of formula and atom_site data

           atom    Z*formula  cif sites diff
           C        910.24    910.19    0.05
           H        692.12    692.13   -0.01
           Cl         8.00      8.00    0.00
```

```
           F         48.00     48.00    0.00
           N        160.00    160.00    0.00
           O        112.00    112.00    0.00
           S         36.12     36.13   -0.01
           Zn        16.00     16.00    0.00
PLAT002_ALERT_2_G Number of Distance or Angle Restraints on AtSite        101 Note
PLAT003_ALERT_2_G Number of Uiso or Uij Restrained non-H Atoms ...        126 Report
PLAT007_ALERT_5_G Number of Unrefined Donor-H Atoms .............           4 Report
PLAT041_ALERT_1_G Calc. and Reported SumFormula    Strings  Differ     Please Check
PLAT128_ALERT_4_G Alternate Setting for Input Space Group     C2/c       I2/a Note
PLAT172_ALERT_4_G The CIF-Embedded .res File Contains DFIX Records         39 Report
PLAT173_ALERT_4_G The CIF-Embedded .res File Contains DANG Records         37 Report
PLAT174_ALERT_4_G The CIF-Embedded .res File Contains FLAT Records          1 Report
PLAT178_ALERT_4_G The CIF-Embedded .res File Contains SIMU Records         13 Report
PLAT186_ALERT_4_G The CIF-Embedded .res File Contains ISOR Records          5 Report
PLAT187_ALERT_4_G The CIF-Embedded .res File Contains RIGU Records         12 Report
PLAT188_ALERT_3_G A Non-default SIMU Restraint Value has been used     0.0200 Report
PLAT244_ALERT_4_G Low    'Solvent' Ueq as Compared to Neighbors of        C2B Check
PLAT244_ALERT_4_G Low    'Solvent' Ueq as Compared to Neighbors of        C3B Check
PLAT301_ALERT_3_G Main Residue  Disorder .............(Resd  1  )          1% Note
PLAT302_ALERT_4_G Anion/Solvent/Minor-Residue Disorder (Resd  2  )       100% Note
PLAT304_ALERT_4_G Non-Integer Number of Atoms in ..... (Resd  2  )       8.81 Check
PLAT335_ALERT_2_G Check Large C6 Ring C-C Range C62      -C67            0.17 Ang.
PLAT371_ALERT_2_G Long   C(sp2)-C(sp1) Bond  C5       - C6       .       1.44 Ang.
PLAT371_ALERT_2_G Long   C(sp2)-C(sp1) Bond  C19      - C21      .       1.46 Ang.
PLAT371_ALERT_2_G Long   C(sp2)-C(sp1) Bond  C22      - C23      .       1.41 Ang.
PLAT371_ALERT_2_G Long   C(sp2)-C(sp1) Bond  C34      - C35      .       1.46 Ang.
PLAT371_ALERT_2_G Long   C(sp2)-C(sp1) Bond  C49      - C50      .       1.46 Ang.
PLAT371_ALERT_2_G Long   C(sp2)-C(sp1) Bond  C59      - C60      .       1.52 Ang.
PLAT371_ALERT_2_G Long   C(sp2)-C(sp1) Bond  C73      - C75      .       1.51 Ang.
PLAT371_ALERT_2_G Long   C(sp2)-C(sp1) Bond  C88      - C89      .       1.46 Ang.
PLAT371_ALERT_2_G Long   C(sp2)-C(sp1) Bond  C100     - C102     .       1.46 Ang.
PLAT412_ALERT_2_G Short Intra XH3 .. XHn     H1L      ..H2B      .       1.93 Ang.
                                                      x,y,z  =      1_555 Check
PLAT412_ALERT_2_G Short Intra XH3 .. XHn     H1M      ..H2B      .       1.56 Ang.
                                                      x,y,z  =      1_555 Check
PLAT412_ALERT_2_G Short Intra XH3 .. XHn     H1N      ..H2B      .       1.22 Ang.
                                                      x,y,z  =      1_555 Check
PLAT432_ALERT_2_G Short Inter X...Y Contact  O2A      ..C81      .       2.99 Ang.
                                                1-x,y,3/2-z  =      2_656 Check
PLAT432_ALERT_2_G Short Inter X...Y Contact  O3A      ..C6A      .       2.59 Ang.
                                                1-x,y,3/2-z  =      2_656 Check
PLAT432_ALERT_2_G Short Inter X...Y Contact  O3A      ..C5A      .       2.88 Ang.
                                                1-x,y,3/2-z  =      2_656 Check
PLAT432_ALERT_2_G Short Inter X...Y Contact  N3       ..C5A      .       2.97 Ang.
                                                1-x,y,3/2-z  =      2_656 Check
PLAT432_ALERT_2_G Short Inter X...Y Contact  C6A      ..C12      .       3.07 Ang.
                                                1-x,y,3/2-z  =      2_656 Check
PLAT606_ALERT_4_G Solvent Accessible VOID(S) in Structure ........          ! Info
PLAT790_ALERT_4_G Centre of Gravity not Within Unit Cell: Resd.  #          5 Note
              Cl O4
PLAT794_ALERT_5_G Tentative Bond Valency for Zn1       (II)      .       1.94 Info
PLAT794_ALERT_5_G Tentative Bond Valency for Zn2       (II)      .       1.98 Info
PLAT802_ALERT_4_G CIF Input Record(s) with more than 80 Characters          3 Info
PLAT860_ALERT_3_G Number of Least-Squares Restraints ............        1165 Note
PLAT869_ALERT_4_G ALERTS Related to the Use of SQUEEZE  Suppressed          ! Info
PLAT883_ALERT_1_G No Info/Value for _atom_sites_solution_primary .     Please Do !
```

```
PLAT933_ALERT_2_G Number of HKL-OMIT Records in Embedded .res File          1 Note
PLAT961_ALERT_5_G Dataset Contains no Negative Intensities .......     Please Check
PLAT978_ALERT_2_G Number C-C Bonds with Positive Residual Density.          0 Info
```

```
  1 ALERT level A = Most likely a serious problem - resolve or explain
  9 ALERT level B = A potentially serious problem, consider carefully
112 ALERT level C = Check. Ensure it is not caused by an omission or oversight
 49 ALERT level G = General information/check it is not something unexpected

  6 ALERT type 1 CIF construction/syntax error, inconsistent or missing data
 85 ALERT type 2 Indicator that the structure model may be wrong or deficient
 15 ALERT type 3 Indicator that the structure quality may be low
 61 ALERT type 4 Improvement, methodology, query or suggestion
  4 ALERT type 5 Informative message, check
```

It is advisable to attempt to resolve as many as possible of the alerts in all categories. Often the minor alerts point to easily fixed oversights, errors and omissions in your CIF or refinement strategy, so attention to these fine details can be worthwhile. In order to resolve some of the more serious problems it may be necessary to carry out additional measurements or structure refinements. However, the purpose of your study may justify the reported deviations and the more serious of these should normally be commented upon in the discussion or experimental section of a paper or in the "special_details" fields of the CIF. checkCIF was carefully designed to identify outliers and unusual parameters, but every test has its limitations and alerts that are not important in a particular case may appear. Conversely, the absence of alerts does not guarantee there are no aspects of the results needing attention. It is up to the individual to critically assess their own results and, if necessary, seek expert advice.

Publication of your CIF in IUCr journals

A basic structural check has been run on your CIF. These basic checks will be run on all CIFs submitted for publication in IUCr journals (*Acta Crystallographica*, *Journal of Applied Crystallography*, *Journal of Synchrotron Radiation*); however, if you intend to submit to *Acta Crystallographica Section C* or *E* or *IUCrData*, you should make sure that full publication checks are run on the final version of your CIF prior to submission.

Publication of your CIF in other journals

Please refer to the *Notes for Authors* of the relevant journal for any special instructions relating to CIF submission.

PLATON version of 06/07/2023; check.def file version of 30/06/2023

Datablock 230707_TTFZn4L8_FeClO4_oxisoli_sqd_sqd - ellipsoid plot

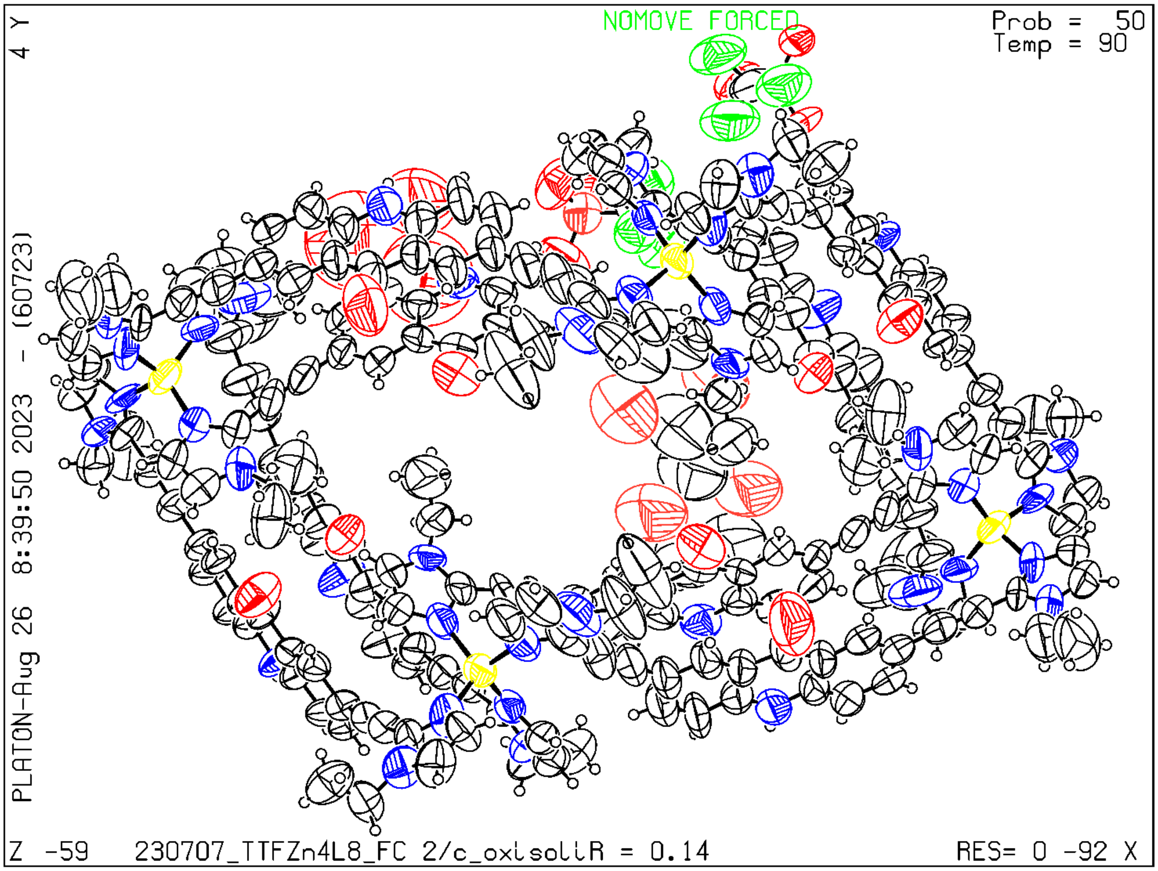

Supplement: Supplementary file 25 — Supplementary data file 24 [file 41467_2024_48599_MOESM25_ESM.pdf]
